# Aberrant somatic calcium channel function in cNurr1 and LRRK2-G2019S mice

**DOI:** 10.1038/s41531-023-00500-5

**Published:** 2023-04-07

**Authors:** Olga Skiteva, Ning Yao, Ioannis Mantas, Xiaoqun Zhang, Thomas Perlmann, Per Svenningsson, Karima Chergui

**Affiliations:** 1 Department of Physiology and Pharmacology, Karolinska Institutet, Stockholm, Sweden; 2 Department of Clinical Neuroscience, Karolinska Institutet, Stockholm, Sweden; 3 Department of Cell and Molecular Biology, Karolinska Institutet, Stockholm, Sweden

**Keywords:** Parkinson's disease, Ion channels in the nervous system

## Abstract

In Parkinson’s disease (PD), axons of dopaminergic (DA) neurons in the substantia nigra pars compacta (SNc) degenerate before their cell bodies. Calcium influx during pacemaker firing might contribute to neuronal loss, but it is not known if dysfunctions of voltage-gated calcium channels (VGCCs) occur in DA neurons somata and axon terminals. We investigated T-type and L-type VGCCs in SNc-DA neurons of two mouse models of PD: mice with a deletion of the *Nurr1* gene in DA neurons from an adult age (cNurr1 mice), and mice bearing the G2019S mutation in the gene coding for LRRK2 (G2019S mice). Adult cNurr1 mice displayed motor and DA deficits, while middle-aged G2019S mice did not. The number and morphology of SNc-DA neurons, most of their intrinsic membrane properties and pacemaker firing were unaltered in cNurr1 and G2019S mice compared to their control and wild-type littermates. L-type VGCCs contributed to the pacemaker firing of SNc-DA neurons in G2019S mice, but not in control, wild-type, and cNurr1 mice. In cNurr1 mice, but not G2019S mice, the contribution of T-type VGCCs to the pacemaker firing of SNc-DA neurons was reduced, and somatic dopamine-D2 autoreceptors desensitized more. Altered contribution of L-type and T-type VGCCs to the pacemaker firing was not observed in the presence of a LRRK2 kinase inhibitor in G2019S mice, and in the presence of a flavonoid with antioxidant activity in G2019S and cNurr1 mice. The role of L-type and T-type VGCCs in controlling dopamine release from axon terminals in the striatum was unaltered in cNurr1 and G2019S mice. Our findings uncover opposite changes, linked to oxidative stress, in the function of two VGCCs in DA neurons somata, but not axon terminals, in two different experimental PD models.

## Introduction

Dopaminergic (DA) neurons in the substantia nigra compacta (SNc) degenerate progressively in patients with Parkinson’s disease (PD). A number of molecular mechanisms were shown to contribute to this neurodegeneration, in particular metabolic and oxidative stress, mitochondrial defects, high intracellular calcium (Ca^2+^) and gene mutations. Several features of SNc-DA neurons predispose them to these degenerative stressors, increasing their vulnerability in PD^1–3^. SNc-DA neurons have an autonomous pacemaking activity, i.e., a regular action potential firing at a frequency of around 1–6 Hz when measured in brain slices, in the absence of synaptic inputs^4^. Ca^2+^ influx through voltage-gated Ca^2+^ channels (VGCCs) during pacemaker firing might play a role in the loss of SNc-DA neurons due to mitochondrial oxidative stress, which contributes to selective vulnerability of these neurons^3,5^. mRNAs for different types of VGCCs (L-type (Cav1.2 and Cav1.3), P/Q-type (Cav2.1), N-type (Cav2.2), R-type (Cav2.3) and T-type (Cav3.1-3.3)) have been detected in SNc-DA neurons^6^. Interestingly, the Cav1.3 pore-forming subunit of L-type VGCCs is not expressed in DA neurons in the ventral tegmental area^7^, which are less affected in PD than SNc-DA neurons. Inhibition of L-type VGCCs with the dihydropyridine isradipine was shown to reduce mitochondrial oxidative stress and to provide neuroprotection in toxin-based PD mouse models^1,8–10^. However, in another study, therapeutically relevant isradipine levels did not provide neuroprotection^11^. Furthermore, other VGCCs might also contribute to increased vulnerability of SNc-DA neurons to degenerative stressors^6,12^. In particular, inhibition of T-type VGCCs was shown to suppress apoptosis of DA neurons derived from PD patients induced by a neurotoxic agent^12^. Our understanding of the role of different VGCCs in the processes that lead to SNc-DA neurons death in PD is incomplete, and several gaps remain unresolved.

PD is a slow progressing neurodegenerative disease and the main risk factor for developing the disease is age. The possibility that an altered role of VGCCs, and of other ion channels, in the pacemaking of SNc-DA neurons in models that recapitulate the slow disease progression and the symptoms of PD, in particular late-onset PD, has not been addressed. Indeed, such electrophysiological studies in middle-aged or aged mice with Parkinsonism are rare. DA neuronal loss causes a dramatic reduction in the content of dopamine in the caudate putamen/striatum, which receive a dense innervation from SNc-DA neurons. It is suggested that the axons of these neurons degenerate first, and that their cell bodies degenerate later, hence the “dying back” process^13^. Indeed, clinical motor signs appear when around 30–50% of SNc neurons and 80% of striatal dopamine are lost, and motor disturbances progressively worsen^14,15^. The questions of whether changes in the functions of VGCCs occur in the axon terminals of SNc-DA neurons of PD models could contribute to the early axonal loss in the striatum, have not been addressed. Differences between somata and axon terminals in the functions and dysfunctions of VGCCs might underlie early vulnerability of SNc-DA axons and the maintenance of their somata.

The use of animal models of PD that replicate the slow developments of the pathology in mice, with a progressive loss of the striatal DA innervation, would allow investigation of the gaps that hamper our understanding of the mechanisms that lead to degeneration. Most cases of PD are sporadic, but mutations of specific genes occur in around 5% of PD patients causing early- or late-onset PD^16^. Thus, mutations of leucine rich repeat kinase 2 (LRRK2, *PARK8*, encoding dardarin protein) are among the most common causes of familial PD, and produce autosomal dominant late-onset PD that is similar to idiopathic PD. The G2019S point mutation in the *LRRK2* gene is a common, and most studied, pathogenic mutation and might increase susceptibility of DA neurons to degeneration^17–19^. A less studied gene which contributes to the pathogenesis of PD is the *NR4A2* gene, which codes for the nuclear receptor-related factor 1 (Nurr1)^20^. Nurr1 is implicated in the acquisition and maintenance of DA phenotype, and its expression is decreased in DA neurons with α-synuclein inclusions in the SNc of patients with PD^21,22^, indicating a role for Nurr1 in the physiopathology of late-onset PD. Mice that carry a deletion of the *Nurr1* gene in midbrain DA neurons from an adult age, cNurr1^DATCreER^ knockout (cNurr1 mice), display robust Parkinsonism with progressive motor impairments and loss of DA innervation of the striatum^23^. The fact that loss-of-function mutations in Nurr1 cause dopa-responsive dystonia and parkinsonism in humans demonstrates a critical role for Nurr1 in maintaining DA functions not only in mice but also in humans^24,25^. On the other hand, mice bearing the G2019S mutation in the *LRRK2* gene (G2019S mice) show Parkinsonism with behavioral and neurochemical DA alterations only at an old age^26–30^. Using cNurr1 and G2019S mice, we sought to determine whether neurophysiological alterations occur in SNc-DA neurons in relation to the age-dependent onset of behavioral and neurochemical alterations. In particular, we tested the hypothesis that ion channels that underlie, or support, the generation of the pacemaker firing of SNc-DA neurons, specifically hyperpolarization-activated and cyclic-nucleotide-modulated (HCN) channels, and L-type and T-type VGCCs, are dysfunctional in PD mice. Identifying neurophysiological changes that occur in different models of PD will provide a better understanding of the mechanisms that might contribute to the loss of SNc-DA neurons somata and axons.

## Results

### cNurr1, but not G2019S, mice display motor deficits and DA biochemical alterations

**Fig. 1:**
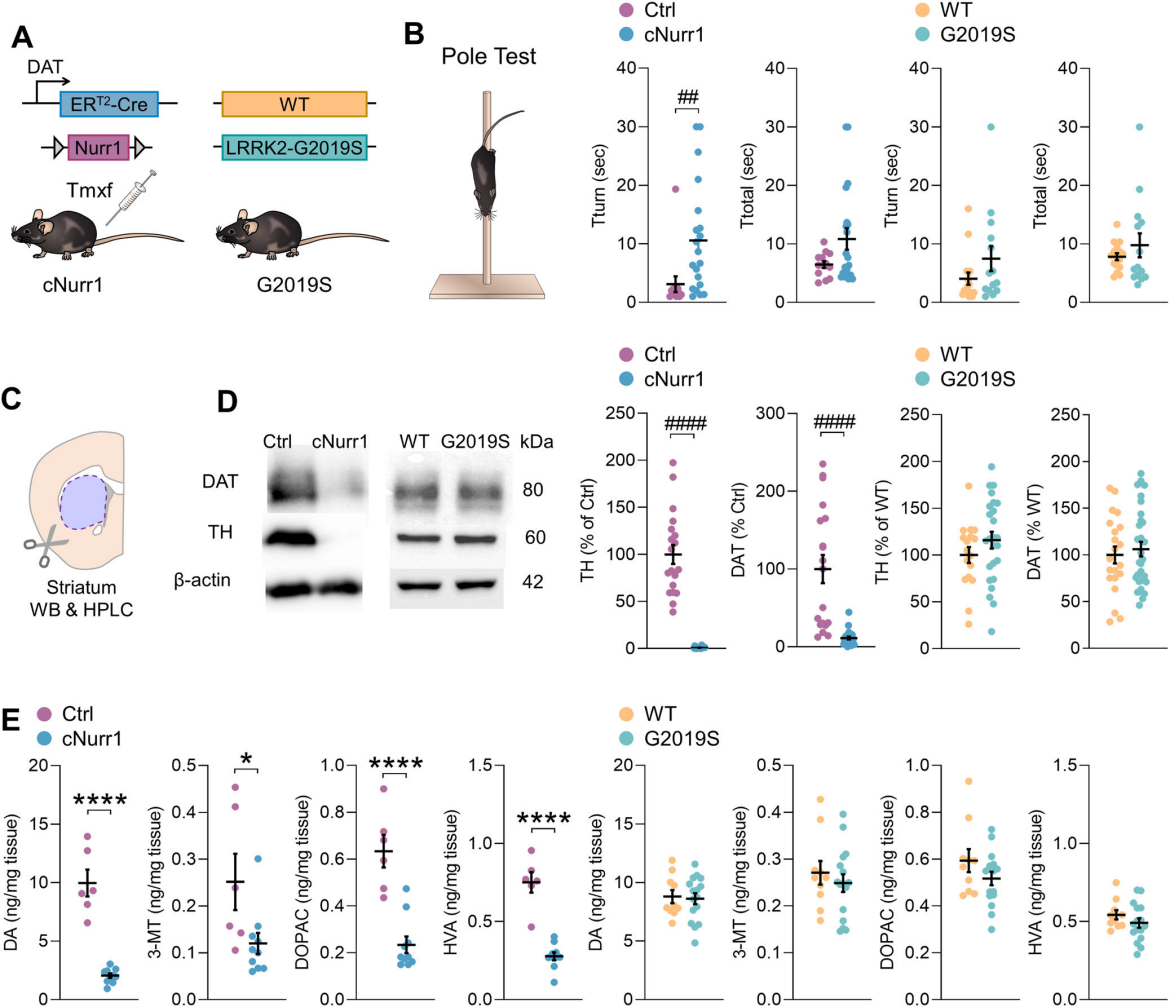
cNurr1, but not G2019S, mice display motor deficits and DA biochemical alterations. **A** Scheme illustrating the cNurr1 and G2019S mouse lines. Tamoxifen (Tmxf) injections produced Cre-mediated ablation of *Nurr1* in mature DA neurons in cNurr1 mice. **B** Fine motor coordination was assessed with the pole test. Tturn: time taken by the mice to turn downward from the top of a vertical pole; Ttotal: total time to descend the pole. *N* = 13 Ctrl, 20 cNurr1, 16 WT and 14 G2019S mice. ^##^*P* < 0.01; Mann–Whitney *U*-test. **C** Schematic representation depicting the striatum, which was dissected for Western blotting (WB) and HPLC experiments. **D** Western blotting of TH in the striatum of *N* = 19 Ctrl, 26 cNurr1, 17 WT and 25 G2019S mice, and DAT in the striatum of *N* = 19 Ctrl, 26 cNurr1, 21 WT and 30 G2019S mice. ^####^*P* < 0.0001; Mann–Whitney *U*-test. **E** Amounts of dopamine and its metabolites 3-MT, DOPAC and HVA measured with HPLC in the striatum of *N* = 6 Ctrl, 10 cNurr1, 10 WT and 16 G2019S mice. ^*^P < 0.05; ^****^P < 0.0001; Unpaired Student’s *t*-test.

Mice with a deletion of the *Nurr1* gene in DA neurons from an adult age (cNurr1^DATCreER^ knockout or cNurr1 mice) display robust Parkinson-like, age-dependent, behavioral, and neurochemical impairments^23^. We used these mice at an age (adult, 6–8-month-old) where motor and DA deficits occur^23^. We also used mice which express the G2019S mutation in the human *LRRK2* gene (G2019S mice), generated via bacterial artificial chromosome (BAC) transgenesis. Middle-aged (10–12 months) G2019S mice do not display motor or neurochemical impairments^30–32^, but previous studies found that Parkinson-like motor deficits occur in aged mice^26–29^. We first assessed motor and DA dysfunctions in our cNurr1 and G2019S cohorts by performing behavioral, neurochemical, and biochemical experiments. As demonstrated previously^23,30,32^, 6–8-month-old cNurr1 mice showed increased time to turn the pole as compared with their control (Ctrl) littermates in the pole test, while 10–12-month-old G2019S mice performed comparatively to their non-transgenic wildtype (WT) littermates (Fig. 1B). Thus, fine motor coordination is impaired in adult cNurr1 mice and is unaffected in middle-aged G2019S mice. Other behavioral tests previously confirmed motor impairments in adult cNurr1 mice and lack of such deficits in middle-aged G2019S mice^23,30,32^. We also examined if motor deficits worsened in older cNurr1 mice. In the pole test, 10–12-month-old cNurr1 mice did not perform worse than 6–8-month-old mice, compared to age-matched Ctrl mice (Supplemental Fig. 1B). In addition, we recently demonstrated that 20–21-month-old G2019S mice performed significantly worse comparatively to their age-matched WT littermates^30^. These results show that motor deficits in cNurr1 mice are stable in older mice and that aged G2019S mice display motor impairments, confirming the pre-symptomatic stage of the 10–12-month-old G2019S group.

Using Western blotting, we found that 6–8-month-old cNurr1 mice had reduced striatal amounts of tyrosine hydroxylase (TH, the rate limiting enzyme in the synthesis of dopamine), and of the dopamine transporter (DAT) (Fig. 1D). These proteins were unaltered in 10–12-month-old G2019S mice (Fig. 1D). To further investigate the DA deficits in these two mouse lines, we measured the amounts of dopamine and its metabolites (3-MT, DOPAC and HVA) in the striatum using HPLC. These amounts were significantly reduced in cNurr1 mice but were unaltered in G2019S mice, compared with ctrl and WT mice, respectively (Fig. 1E), further confirming the occurrence of DA deficits in cNurr1 mice, and the absence of such deficits in G2019S mice.

### SNc-DA neurons in cNurr1 and G2019S mice have intact cell count and morphology but display reduced TH mRNA

**Fig. 2:**
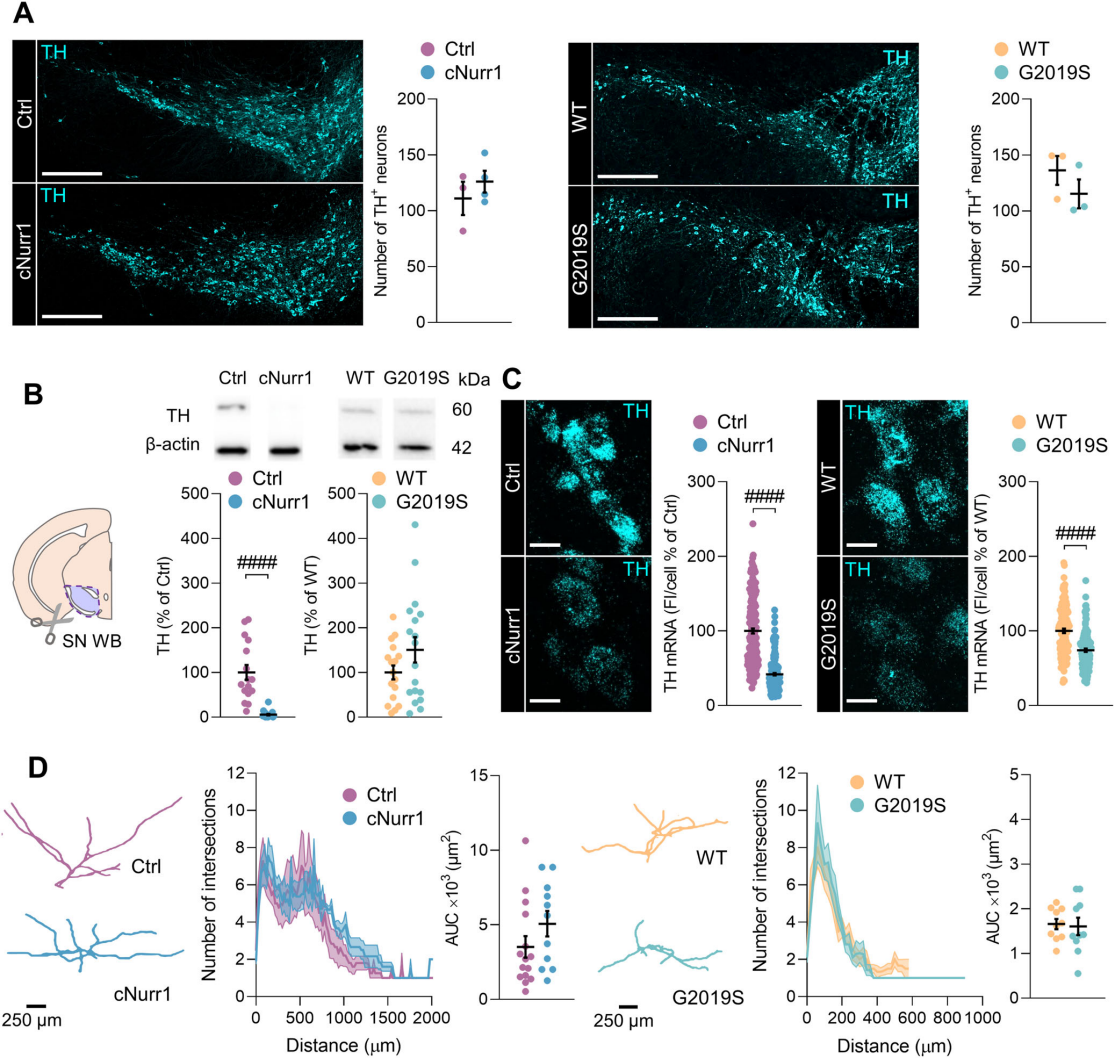
SNc-DA neurons in cNurr1 and G2019S mice have intact cell count and morphology but display reduced TH mRNA. **A** TH immunofluorescence in midbrain sections containing the SNc; scale bars, 500 µm. Number of TH-positive neurons in the SNc of *N* = 3 Ctrl, 4 cNurr1, 3 WT and 3 G2019S mice. **B** Western blotting of TH in the ventral midbrain region containing the SNc of *N* = 16 Ctrl, 19 cNurr1, 17 WT and 18 G2019S mice. ^####^*P* < 0.0001; Mann–Whitney *U*-test. **C** Representative images of neurons in the SNc from FISH experiments of TH mRNA; scale bars, 15 µm. Quantification of fluorescence in individual TH-containing cells in the SNc. *n* = 186, 185, 135, 126 TH-positive neurons from *N* = 3 Ctrl, 3 cNurr1, 3 WT and 3 G2019S mice. ^####^*P* < 0.0001; Mann–Whitney *U*-test. **D** Representative drawings illustrating the dendritic arborization of neurobiotin-injected neurons in the SNc of Ctrl, cNurr1, WT and G2019S mice. Sholl analysis shows the number of intersections and area under the curve (AUC) measured in *n* = 15, 11, 10, 10 neurobiotin-injected neurons from *N* = 6 Ctrl, 8 cNurr1, 4 WT and 4 G2019S mice.

We asked if degeneration of SNc-DA neurons cell bodies occurred in cNurr1 and G2019S mice. Using immunohistochemistry and FISH, we found that the numbers of TH-positive neurons in the SNc were similar in adult cNurr1 and Ctrl mice (Fig. 2A; Supplemental Fig. 1C), and that older (10–12 months) cNurr1 mice did not show SNc-DA neuron loss either (Supplemental Fig. 1D). Middle-aged G2019S mice did not show loss of SNc-DA neurons compared with their WT littermates (Fig. 2A; Supplemental Fig. 1C). The amounts of TH were reduced in the midbrain of adult cNurr1 mice but not middle-aged G2019S mice (Fig. 2B). Interestingly, the mRNA expression of TH in individual SNc neurons was decreased in both cNurr1 and G2019S mice (Fig. 2C). Although we did not analyze the amounts of TH in SNc neurons from our immunohistochemical experiments, due to technical limitations, our results obtained with Western blotting and FISH show that DA neurochemical alterations, but not somatic degeneration, occur in SNc-DA neurons of cNurr1 and G2019S mice. We then examined if SNc-DA neurons in cNurr1 and G2019S mice displayed an altered dendritic morphology given that this characteristic is associated with neurodegenerative diseases^33^. We performed morphometric analysis of the dendritic arborization of SNc-DA neurons previously recorded with a patch pipette filled with neurobiotin. Sholl analysis of these neurobiotin-labeled SNc-DA neurons showed an unaltered number of intersections and area under the curve in cNurr1 and G2019S mice, compared with Ctrl and WT mice (Fig. 2D). The dendritic arborization of WT and G2019S was smaller than that of Ctrl and cNurr1 mice. This was not due to the age difference between the two mouse lines because the dendritic arborization of adult (8-months-old) LRRK2 mice was similar to that of 10–12 months old mice (Supplemental Fig. 1E).

### SNc-DA neurons have unaltered Ih currents in cNurr1 and G2019S mice but display an increased slow AHP in cNurr1 mice

**Fig. 3:**
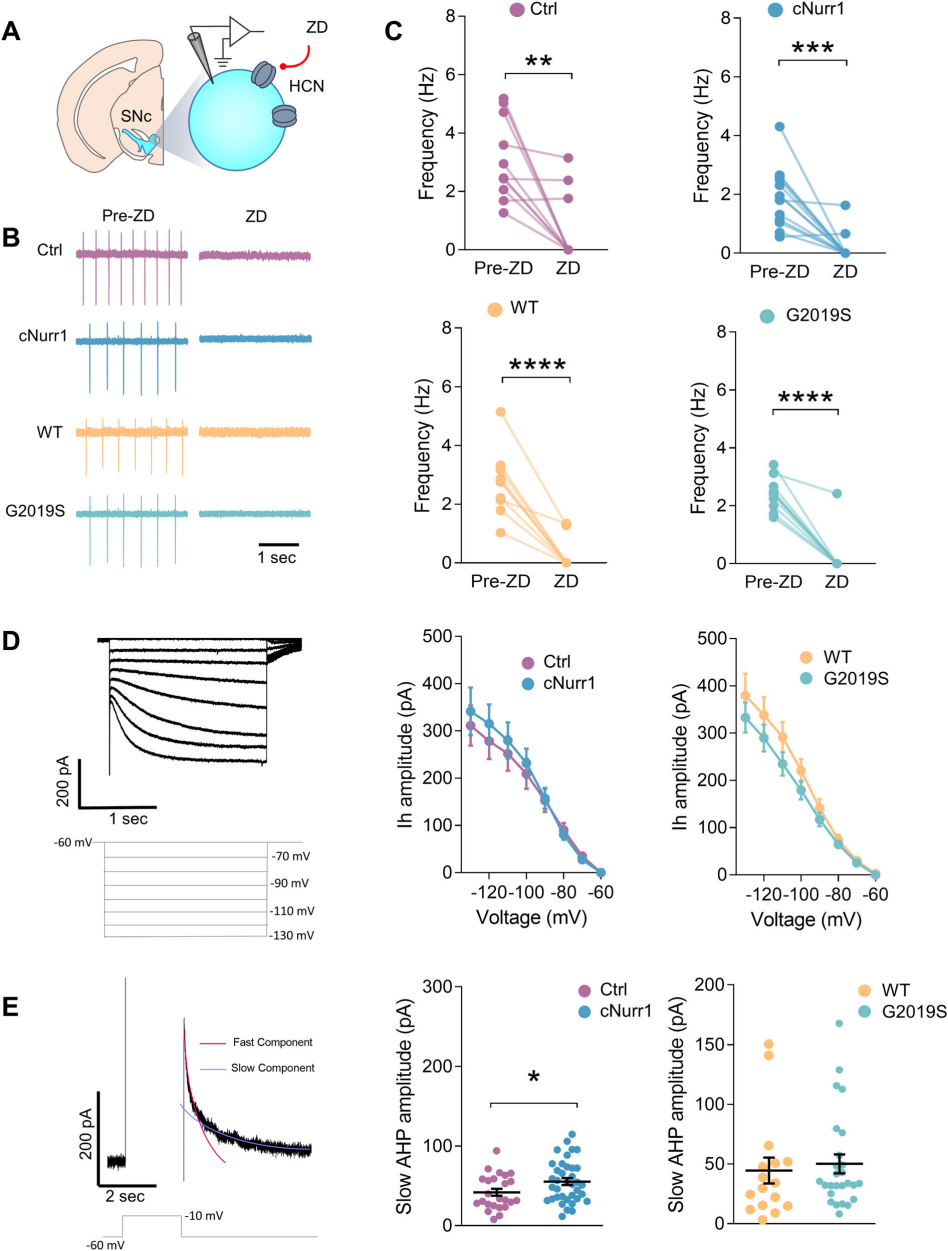
SNc-DA neurons have unaltered Ih currents in cNurr1 and G2019S mice but display an increased slow AHP in cNurr1 mice. **A** Schematic representation depicting the SNc and a DA neuron with a patch clamp recording electrode. ZD 7288 (ZD) was used to block HCN channels. **B** Representative traces of pacemaker firing recorded in the cell-attached mode in four SNc-DA neurons from Ctrl, cNurr1, WT and G2019S mice before (Pre-ZD) and during (ZD) the perfusion with ZD 7288 (50 μM). **C** Firing frequency of individual SNc-DA neurons before and during perfusion with ZD 7288. *n* = 11, 13, 10, 11 neurons from *N* = 5 Ctrl, 6 cNurr1, 3 WT and 4 G2019S mice. ^**^P < 0.01, ^***^P < 0.001, ^****^P < 0.0001; Paired Student’s *t*-test. **D** Representative traces of Ih currents measured in whole-cell voltage-clamp mode at different hyperpolarizing voltage steps (bottom traces, from a holding potential of −60 mV to −130 mV with 10 mV increments, 2 s duration). Graphs show Ih amplitude at varying voltage steps measured in *n* = 18, 11, 34, 21 SNc-DA neurons from *N* = 4 Ctrl, 2 cNurr1, 8 WT and 4 G2019S mice. **E** Representative trace of an AHP current measured in voltage-clamp mode and evoked by a depolarizing voltage step (bottom trace, from a holding potential of −60 mV to −10 mV, 1 s duration). Red and blue lines illustrate fast and slow components of the AHP current. Graphs show slow AHP current amplitude measured in *n* = 24, 37, 16, 26 SNc-DA neurons from *N* = 9 Ctrl, 11 cNurr1, 5 WT and 8 G2019S mice. ^*^P < 0.05; Unpaired Student’s *t*-test.

The pacemaker firing of SNc-DA neurons, as well as its regularity and pauses in firing, are dependent upon a proper function of several ion channels^34^. Using somatic cell-attached and whole-cell patch clamp recordings in brain slices, we found that the membrane properties, pacemaker firing frequency and regularity of firing (measured with coefficient of variation of interspike intervals) are similar in cNurr1 mice and G2019S mice compared with their Ctrl and WT littermates (Supplemental Table 1). The presence of Ih currents characterizes DA neurons in the SNc and allows for their electrophysiological identification and distinction from other neurons in this brain region^35^. HCN channels are Na^+^- and K^+^-permeable, underlie Ih currents, and were shown in some studies to contribute to the pacemaker firing in a population of SNc-DA neurons^35,36^. In the presence of the HCN channel blocker ZD 7288 (50 μM), the pacemaker firing was reduced in most SNc-DA neurons from Ctrl and WT mice as well as cNurr1 and G2019S mice (Fig. 3B, C). Moreover, we found no differences between Ctrl and cNurr1 mice, and between WT and G2019S mice in the amplitude of Ih currents evoked by negative voltage steps of increasing magnitude (Fig. 3D). These results demonstrate the involvement of HCN channels in the pacemaker firing of SNc-DA neurons of control mice, and an unaltered function of these channels in cNurr1 and G2019S mice. We then examined if other neurophysiological properties of SNc-DA neurons were altered in cNurr1 and G2019S mice. We found that action potential properties (Supplemental Fig. 2) and excitability (Supplemental Fig. 3) were unaltered. We measured afterhyperpolarization (AHP) currents in voltage-clamp mode after 2 sec-long positive voltage steps of varying amplitudes and found that the total amplitude of these currents was similar in cNurr1 and G2019S mice compared to Ctrl and WT mice, respectively, across the voltage range used (Supplemental Fig. 4A, B). When we extracted the fast and slow components of AHP currents measured after a -60 to -10 mV voltage step (2 sec), we found that the amplitude of the slow component (Fig. 3E), but not the fast component (Supplemental Fig. 4C), was significantly increased in cNurr1 mice compared with Ctrl mice. No changes were observed in G2019S mice (Fig. 3E; Supplemental Fig. 4C).

### Increased function of L-type VGCCs in SNc-DA neurons of G2019S mice but not cNurr1 mice

**Fig. 4:**
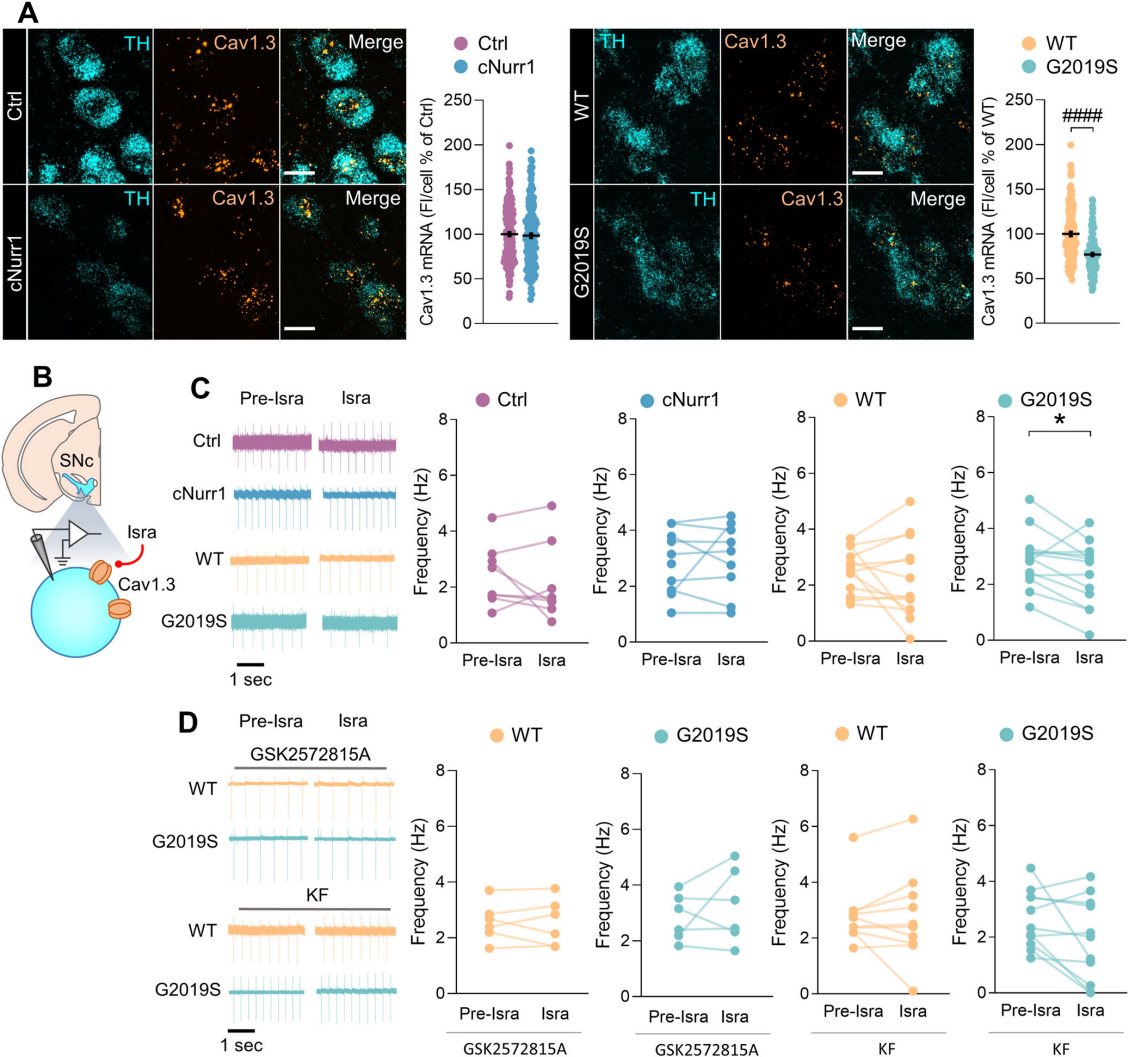
Increased contribution of L-type VGCCs in SNc-DA neurons of G2019S mice but not cNurr1 mice. **A** Representative images of neurons in the SNc from double FISH experiments of CACNA1D mRNA, which encodes the Cav1.3 L-type channel, and TH mRNA; scale bars, 15 µm. Quantification of fluorescence for CACNA1D mRNA in individual TH-containing cells in the SNc. *n* = 186, 185, 135, 126 neurons from *N* = 3 Ctrl, 3 cNurr1, 3 WT and 3 G2019S mice. ^####^*P* < 0.0001; Mann–Whitney *U*-test. **B** Schematic representation depicting the SNc and a DA neuron. Isradipine (Isra) was used to block Cav1.3 L-type channels. **C**, **D** Representative traces of pacemaker firing recorded in the cell-attached mode in SNc-DA neurons from Ctrl, cNurr1, WT and G2019S mice before (Pre-Isra) and during (Isra) the perfusion with isradipine (0.2 μM) in control condition (aCSF, **C**), in slices incubated with the LRRK2 kinase inhibitor GSK2578215A (1 μM, **D**) or with kaempferol (KF, 5 μM, **D**). Graphs show the firing frequency of individual SNc-DA neurons before (Pre-Isra) and during (Isra) the perfusion with Isradipine. Cells recorded in aCSF: *n* = 8, 9, 13, 13 neurons from *N* = 6 Ctrl, 8 cNurr1, 4 WT and 5 G2019S mice (**C**). ^*^P < 0.05; Paired Student’s *t*-test. Cells recorded in GSK2578215A: *n* = 6 and 6 neurons from *N* = 2 WT and 4 G2019S mice. Cells recorded in kaempferol: *n* = 10 and 11 neurons from *N* = 5 WT and 5 G2019S mice (**D**).

Ca^2+^ entry, through VGCCs, into the cytoplasm of SNc-DA neurons during autonomous, pacemaker firing is suggested to contribute to increased vulnerability of these neurons to PD triggers^3,5^. The Cav1.3 L-type VGCC was proposed for neuroprotective therapy in patients with PD^9,10,37^. We examined if the expression and the function of L-type VGCCs are altered in SNc-DA neurons of cNurr1 and G2019S mice. Using FISH, we found that TH-positive cells in the SNc express mRNA for CACNA1D, the gene that encodes Cav1.3 (Fig. 4A). We found no differences in the fluorescence within individual cells in cNurr1 mice, but a decrease was observed in G2019S mice (Fig. 4A). We evaluated the contribution of Cav1.3 in the pacemaker firing of SNc-DA neurons by using cell-attached recordings and isradipine, a compound shown to block Cav1.3 channels in SNc-DA neurons when applied at a low concentration^38^. We found that Cav1.3 L-type VGCCs do not contribute to the pacemaker firing of SNc-DA neurons in cNurr1, Ctrl and WT mice because isradipine (0.2 μM) failed to affect this activity (Fig. 4C). However, isradipine reduced the pacemaker firing of SNc-DA neurons in G2019S mice (Fig. 4C), demonstrating a contribution of the Cav1.3 L-type VGCC in the pacemaker firing in these mice.

The G2019S mutation in the *LRRK2* gene affects the serine/threonine kinase domain of the LRRK2 protein which leads to increased kinase activity^39^. Increased LRRK2 kinase activity and G2019S mutation are also observed in sporadic PD^17,40^, demonstrating the importance of LRRK2 in the pathogenesis of the disease. We examined if the increased L-type VGCC function in G2019S mice could be a consequence of an increased LRRK2 kinase activity. We found that in slices incubated with the LRRK2 kinase inhibitor GSK2578215A (1 μM), isradipine (0.2 μM) did not decrease the pacemaker firing of SNc-DA neurons (Fig. 4D). It is not clear whether Ca^2+^ dysfunction causes Parkinsonism or is secondary to the disease function^41^. To examine the possibility that the change in L-type VGCC function in G2019S mice is linked to oxidative stress, we used kaempferol, a flavonoid with antioxidant activity^42^. We first demonstrated that slices incubated with kaempferol (5 μM) had unaltered striatal DA markers (Supplemental Fig. 5A). We then found that in slices incubated with kaempferol, isradipine had no effect on the pacemaker firing of SNc-DA neurons of G2019S and WT mice (Fig. 4D). In an attempt to determine if oxidative stress occurs in G2019S mice, we examined the amounts of Nuclear factor-erythroid 2-related factor 2 (Nrf2), a transcription factor involved in resistance to oxidative stress^43^. We found no differences in the amounts of Nrf2 in the striatum and substantia nigra between G2019S and WT mice (Supplemental Fig. 5B). Nevertheless, our results with GSK2578215 A and kaempferol suggest that the increased L-type VGCC function in G2019S mice is likely due to an increased LRRK2 kinase activity and might be associated with oxidative stress.

### Reduced function of T-type VGCCs in SNc-DA neurons of cNurr1 mice but not G2019S mice

**Fig. 5:**
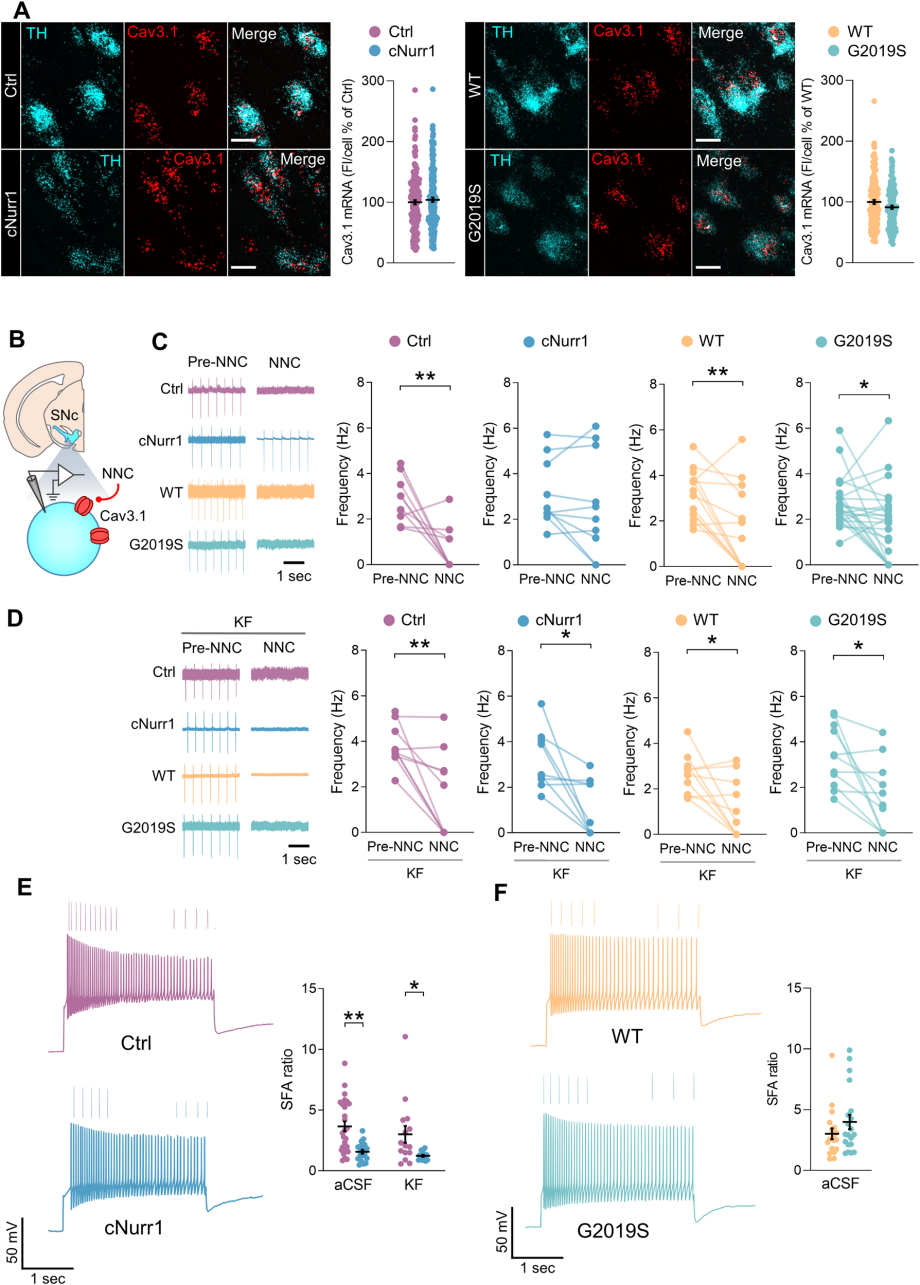
Reduced contribution of T-type VGCCs in SNc-DA neurons of cNurr1 mice but not G2019S mice. **A** Representative images of neurons in the SNc from double FISH experiments of CACNA1G mRNA, which encodes the Cav3.1 T-type channel, and TH mRNA; scale bars, 15 µm. Quantification of fluorescence for CACNA1G mRNA in individual TH-containing cells in the SNc. *n* = 164, 186, 141, 144 neurons from *N* = 3 Ctrl, 3 cNurr1, 3 WT and 3 G2019S mice. **B** Schematic representation depicting the SNc and a DA neuron. NNC 55-0396 (NNC) was used to block Cav3.1 T-type channels. **C**, **D** Representative traces of pacemaker firing recorded in the cell-attached mode in SNc-DA neurons from Ctrl, cNurr1, WT and G2019S mice before (Pre-NNC) and during (NNC) the perfusion with NNC 55-0396 (10 μM) in control condition (aCSF, **C**), and in slices incubated with kaempferol (KF, 5 μM, **D**). Graphs show the firing frequency of individual SNc-DA neurons before (Pre-NNC) and during (NNC) the perfusion with NNC 55-0396. Cells recorded in aCSF: *n* = 10, 10, 14, 25 neurons from *N* = 5 Ctrl, 7 cNurr1, 5 WT and 10 G2019S mice (**C**). Cells recorded in kaempferol: *n* = 10, 10, 10, 11 neurons from *N* = 7 Ctrl, 5 cNurr1, 5 WT and 4 G2019S mice (**D**). ^*^P < 0.05; ^**^P < 0.01, Paired Student’s *t*-test. **E**, **F** Representative current-clamp recordings of the firing of SNc-DA neurons during a hyperpolarization-depolarization step protocol. Insets above the traces are magnified firing at the beginning and at the end of the positive current steps. Graphs show the spike firing adaptation (SFA) ratio calculated by dividing the interval between the last two action potentials with the interval between the first two action potentials. *n* = 28 and 20 neurons from *N* = 10 Ctrl and 8 cNurr1 mice in aCSF; *n* = 15 and 14 neurons from *N* = 7 Ctrl and 4 cNurr1 mice in kaempferol (KF, **E**). ^*^P < 0.05; ^**^P < 0.01, Two-way ANOVA followed by Multiple comparisons (Tukey). *n* = 19 and 20 neurons from *N* = 5 WT and 4 G2019S mice (**F**).

T-type VGCCs (Cav3.1-3.3) might contribute to increased vulnerability of SNc-DA neurons to degenerative stressors^12^. We therefore examined if the function of these channels was altered in SNc-DA neurons of cNurr1 and G2019S mice. mRNAs of *CACNA1G*, the gene which encodes Cav3.1, were expressed in TH-positive cells in the SNc with no differences between cNurr1 and Ctrl mice and between G2019S and WT mice (Fig. 5A). Perfusion with the Cav3.1 T-type VGCC blocker NNC 55-0396 (10 μM) decreased the pacemaker firing frequency of most SNc-DA neurons in Ctrl and WT mice (Fig. 5C), demonstrating a role for T-type VGCCs in the autonomous pacemaker activity of these neurons. However, the inhibitory effect of NNC 55-0396 was absent in cNurr1 mice and was unaltered in G2019S mice (Fig. 5C). In slices incubated with kaempferol, NNC 55-0396 reduced the pacemaker firing of SNc-DA neurons in cNurr1 mice, as well as Ctrl, WT and G2019S mice (Fig. 5D). This result suggests that the decreased contribution of T-type VGCC to autonomous pacemaker firing of SNc-DA neurons in cNurr1 mice might be linked to oxidative stress. Interestingly, we found that the amounts of Nrf2 are increased in the striatum, but not in the substantia nigra, of cNurr1 mice (Supplemental Fig. 5B).

T-type VGCCs are involved in neuronal oscillations and rebound burst firing in various types of neurons^44,45^. We examined rebound firing following a hyperpolarization-depolarization step protocol^45^. Hyperpolarization of the membrane allowed removal of T-type channel inactivation, and thereby activation of a T-type current by a following depolarization^34^. In this protocol, the current used to depolarize the membrane (for 3 sec) had the same amplitude as the current used to hyperpolarize the membrane to −80 mV (for 10 sec). We found that in SNc-DA neurons of cNurr1 mice, but not in G2019S mice, the firing frequency at the beginning of the depolarization was reduced, compared to Ctrl mice, resulting in a lower spike frequency adaptation ratio (Fig. 5E, F). In slices incubated with kaempferol, the spike frequency adaptation ratio was still lower in cNurr1 mice compared to Ctrl mice (Fig. 5E). These results, obtained with two different approaches, i.e., pharmacological, and physiological, demonstrate that SNc-DA neurons in cNurr1 mice have a reduced function of T-type VGCCs.

### Decreased DA-D2 autoreceptor function in SNc-DA neurons of cNurr1 mice

**Fig. 6:**
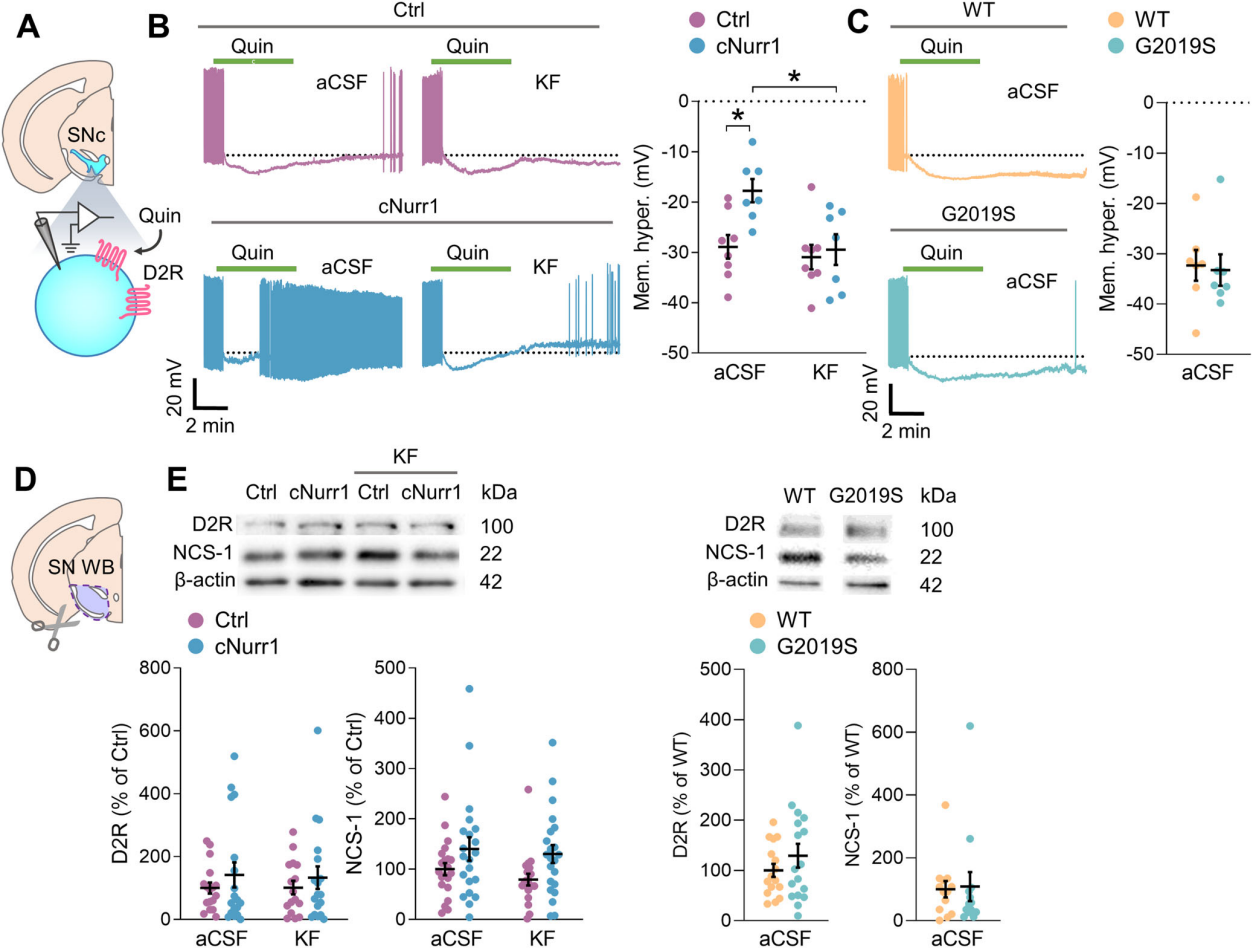
Decreased DA-D2 autoreceptor function in SNc-DA neurons of cNurr1 mice. **A** Schematic representation depicting the SNc and a DA neuron. The DA-D2 receptor (D2R) agonist Quinpirole (Quin) was used in this experiment. **B**, **C** Representative current-clamp recordings of the pacemaker firing measured at resting membrane potential (dotted lines) before, during and after the perfusion with quinpirole (10 μM, 60 sec) in control slices (aCSF) and in slices incubated with kaempferol (KF, 5 μM). Graphs show the amplitude of the quinpirole-induced membrane hyperpolarization. *n* = 8 and 7 neurons in aCSF from *N* = 4 Ctrl and 3 cNurr1 mice; *n* = 8 and 7 neurons in kaempferol from *N* = 3 Ctrl and 6 cNurr1 mice (**B**). ^*^P < 0.05; Two-way ANOVA followed by Multiple comparisons (Tukey). *n* = 7 and 7 neurons in aCSF from *N* = 6 WT and 5 G2019S mice (**C**). **D**, **E** Western blotting of D2R and NCS-1 in the ventral midbrain region containing the SNc of *N* = 17 Ctrl, 18 cNurr1, 16 WT and 17 G2019S mice from control conditions (aCSF) and *N* = 16 Ctrl and 18 cNurr1 mice from slices incubated in kaempferol (KF).

We investigated possible physiological consequences of aberrant VGCC function in SNc-DA neurons of cNurr1 and G2019S mice. L-type and T-type VGCCs regulate DA-D2 autoreceptor sensitization through Ca^2+^-dependent interactions between the neuronal Ca^2+^ sensor NCS-1 and DA-D2 receptors^3,38,46,47^. We examined if sensitization of DA-D2 autoreceptors was altered in SNc-DA neurons of cNurr1 and G2019S mice by using the agonist quinpirole. Perfusion with quinpirole (10 μM) induced a membrane hyperpolarization and inhibited the pacemaker firing of SNc-DA neurons in Ctrl and WT mice (Fig. 6B, C). However, membrane hyperpolarization was significantly reduced in cNurr1 mice compared to Ctrl mice (Fig. 6B). In slices incubated in kaempferol, membrane hyperpolarization induced by quinpirole was similar in cNurr1 and Ctrl mice (Fig. 6B), suggesting that the reduced effect of quinpirole in cNurr1 mice might be linked to oxidative stress. Quinpirole-induced membrane hyperpolarization was similar in WT and G2019S mice (Fig. 6C). To examine if this lack of difference was due to the supramaximal concentration of quinpirole used^48^, which might cause autoreceptor desensitization during the application, we tested the effect of a lower quinpirole concentration. At 100 nM, quinpirole-induced membrane hyperpolarization had a similar amplitude in WT and G2019S mice (Supplemental Fig. 6). We performed Western Blotting experiments to determine if the reduced effect of quinpirole in cNurr1 mice was associated with altered amounts of DA-D2 receptors and NCS-1. We found no differences in these measures between cNurr1 and Ctrl mice and between G2019S and WT mice (Fig. 6E). These results demonstrate that DA-D2 autoreceptors desensitize more in SNc-DA neurons of cNurr1 compared with Ctrl mice, but not in G2019S mice.

### Unaltered role of L-type and T-type channels in the control of dopamine release from axon terminals

**Fig. 7:**
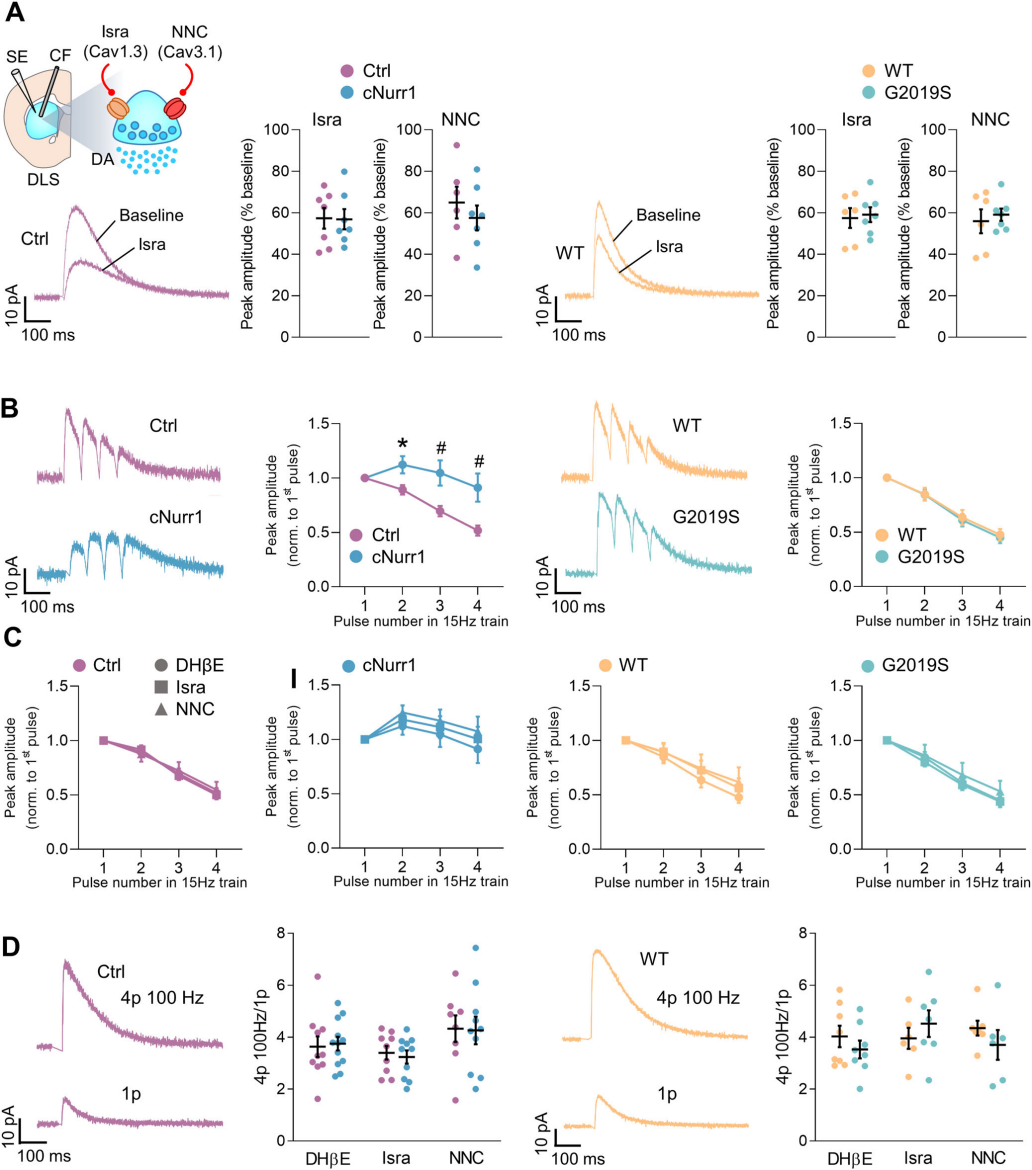
Unaltered role of L-type and T-type channels in the control of dopamine release from axon terminals. **A** Schematic representation of the experiment. A carbon fiber electrode (CF) and a stimulating electrode (SE) were placed in the dorsolateral striatum in coronal brain slices. The experiments were performed in the presence of the nicotinic acetylcholine receptor antagonist DhβE (0.1 μM). Traces are representative amperometric recordings of dopamine release evoked by single stimulation pulses before (Baseline) and during the application of isradipine (Isra). Graphs show the average magnitude of the effect of isradipine (0.2 μM) and NNC 55-0396 (10 μM) on dopamine release evoked by single stimulation pulses. Isradipine: *n* = 7, 7, 6, 7 slices from *N* = 4 Ctrl, 4 cNurr1, 4 WT and 3 G2019S mice. NNC 55-0396: *n* = 6, 7, 6, 7 slices from *N* = 5 Ctrl, 6 cNurr1, 5 WT and 4 G2019S mice. **B** Traces are representative amperometric recordings of dopamine release evoked by trains of four pulses at 15 Hz. Graphs show the average peak amplitude evoked by individual pulses within 15 Hz trains, normalized to the amplitude of the peak evoked by the first pulse in the train. *n* = 10, 15, 6, 8 slices from *N* = 8 Ctrl, 12 cNurr1, 5 WT and 7 G2019S mice. ^*^P < 0.05; Unpaired t Student’s *t*-test. ^#^*P* < 0.05; Mann–Whitney *U*-test. **C** Average peak amplitude evoked by individual pulses within 15 Hz trains, normalized to the amplitude of the peak evoked by the first pulse in the train. In DhβE (0.1 μM): same as in (**B**). In isradipine (0.2 μM): *n* = 9, 11, 5, 8 slices from *N* = 5 Ctrl, 5 cNurr1, 3 WT and 4 G2019S mice. In NNC 55-0396 (10 μM): *n* = 8, 11, 7, 6 slices from *N* = 6 Ctrl, 5 cNurr1, 5 WT and 4 G2019S mice. **D** Traces are representative amperometric recordings of dopamine release evoked by trains of four pulses at 100 Hz and by single stimulation pulses (1p). Graphs show the average peak amplitude evoked by 100 Hz trains, normalized to the amplitude of the peak evoked by single pulses. In DhβE (0.1 μM): *n* = 10, 12, 8, 8 slices from *N* = 8 Ctrl, 10 cNurr1, 7 WT and 7 G2019S mice. In isradipine (0.2 μM): *n* = 9, 10, 6, 7 slices from *N* = 5 Ctrl, 4 cNurr1, 3 WT and 5 G2019S mice. In NNC 55-0396 (10 μM): *n* = 8, 10, 7, 6 slices from *N* = 6 Ctrl, 6 cNurr1, 5 WT and 4 G2019S mice.

In PD patients and in models of PD, axon terminals of SNc-DA neurons degenerate before their somata. This suggests the delayed degenerative processes in cell bodies is due to degenerative or protective mechanisms engaged in the somata or in the axon terminals. Although N- and P/Q-type VGCCs govern neurotransmitter release at most synapses in the CNS, L-type and T-type VGCCs were shown to be present in certain presynaptic terminals where they play a role in release mechanisms^49^. In particular, inhibition of these channels decreases the release of dopamine by axon terminals in the striatum^50^. We therefore examined if the role of L-type and T-type channels in the modulation of dopamine release in the striatum was altered in cNurr1 and G2019S mice. We employed amperometry in brain slices to measure stimulation-evoked dopamine release in the presence of the nicotinic receptor antagonist DhβE (0.1 μM) to block the control of dopamine release by cholinergic interneurons in the striatum through nicotinic receptors. In cNurr1 mice, dopamine release originated from residual axon terminals because the amount of DA markers and of dopamine were reduced (Fig. 1D, E), indicating degeneration of most striatal DA axons. In older (10–12 months) cNurr1 mice, the amounts of striatal dopamine measured with HPLC were not further decreased compared to 6–8-months old mice (Supplemental Fig. 7), indicating that the axons from which we measured dopamine release were preserved with time. Isradipine (0.2 μM) and NNC 55-0396 (10 μM) decreased dopamine release evoked by single stimulation pulses in the dorsolateral striatum of cNurr1 mice, and their controls, as well as in G2019S mice and their WT littermates. There was no difference in the ability of isradipine and NNC 55-0396 to decrease dopamine release in the four groups of mice examined (Fig. 7A). We then assessed the effect of isradipine and NNC 55-0396 on dopamine release evoked by low- and high-frequency stimulation trains to mimic the in vivo firing pattern of DA neurons. Indeed, when recorded in vivo, these neurons fire action potential in a phasic (bursting), as well as tonic (regular) mode^51,52^. Moreover, VGCCs might control dopamine release in a frequency-dependent manner^50^. We first observed that dopamine release evoked by trains of four pulses at 15 Hz was increased in the dorsolateral striatum of cNurr1 mice as compared to Ctrl mice, but not in the dorsolateral striatum of G2019S mice (Fig. 7B). Dopamine release evoked by 100 Hz trains was similar in the four groups of mice examined (Fig. 7D). Isradipine and NNC 55-0396 did not alter dopamine release evoked by 15 Hz and 100 Hz trains in cNurr1 mice and in G2019S mice, as compared to Ctrl and WT mice, respectively (Fig. 7C, D).

## Discussion

VGCCs are pharmacological targets for the treatment of PD. Whether dysfunctions of these channels occur in DA neurons in models that recapitulate the slow development and late-onset of this disease is largely unknown. In this study, we demonstrate that an altered function of somatic L-type and T-type VGCCs occurs in opposite ways in two different PD models. Specifically, cNurr1 mice have a reduced function of T-type channels while an increased function of L-type channels is observed in G2019S mice. Interestingly, the role of L-type and T-type VGCCs in the control of dopamine release from axon terminals in the striatum is unaltered in cNurr1 and G2019S mice. Our results demonstrate that altered VGCCs function, resulting from oxidative stress and possibly leading to intracellular Ca^2+^ dyshomeostasis, affects SNc-DA neurons somata but not their axon terminals in both cNurr1 and G2019S mice.

Nurr1, a transcription factor of the NR4A subgroup of nuclear receptor superfamily, plays key roles in the development and maintenance of DA neurons^20,22,53^. *Nurr1* deletion in adult DA neurons gives rise to behavioral impairments, DA deficits including decreased TH, DAT and VMAT2 in the striatum, and loss of DA fibers in the midbrain and striatum^23,54^. The differential expression of nuclear-encoded mitochondrial genes in SNc-DA neurons from cNurr1 mice suggests an involvement of Nurr1 in oxidative respiration^23^. The precise mechanisms involved in DA deficits and Parkinsonism in cNurr1 mice are still not elucidated, but our study suggests a link between Nurr1 and maintenance of T-type VGCCs function in SNc-DA neurons. Both *Nurr1* deletion in DA neurons and the G2019S mutation in the *LRRK2* gene seem to interfere with mitochondrial homeostasis and lead to increased oxidative stress^55^. However, these gene modifications lead to alterations of the function of different VGCCs, in opposite direction, which indicates the involvement of different pathways specific for Nurr1 and LRRK2. LRRK2 is a large multi-domain protein, with a GTPase domain and a serine-threonine kinase domain which confer to the protein multiple roles, many of which remain to be elucidated. LRRK2 is involved in vesicle trafficking and was suggested to regulate synaptic vesicle endocytosis through phosphorylation of proteins involved in this process^56^. LRRK2 also plays roles in Ca^2+^ signaling and homeostasis. Altered LRRK2 kinase function, due to, e.g., G2019S mutation, might thus impair proper synaptic vesicle endocytosis, Ca^2+^ buffering capacity, increase neuronal vulnerability to oxidative stress, and contribute to degeneration of DA neurons^55,56^. How these altered LRRK2 functions contribute to increased L-type VGCC function in SNc-DA neurons remains to be examined, but our study indicates a role for phosphorylation of LRRK2 substrates and oxidative stress. Direct action of LRRK2 on L-type VGCCs through physical interactions, as was demonstrated for the P/Q-type (Cav2.1) VGCC in HEK293 cells^57^, might also contribute to changes in the function of this channel in G2019S mice.

Our behavioral and neurochemical observations confirmed previously described deficits associated with degenerative processes in cNurr1 mice^23,54^ and lack of such impairments in G2019S mice^30,32^. This indicates that in cNurr1 mice, the neurophysiological changes observed are associated with impaired motor behavior, reduced striatal DA innervation, and reduced TH expression in SNc-DA neurons. In G2019S mice, neurophysiological alterations develop before the onset of motor deficits and striatal DA denervation. We found that SNc-DA neurons somata and dendritic arborization are preserved in these two mouse lines. An earlier study reported abnormal dendritic integrity in cNurr1 mice^23^, which we did not observe in our study. This discrepancy is likely due to differences in the experimental conditions, in particular the age of the mice and the use of intracellular labeling with neurobiotin vs. TH immunostaining. Nevertheless, in both cNurr1 and G2019S mice, we found that the amounts of TH and/or of TH mRNA in SNc-DA neurons are decreased, demonstrating neurochemical alterations even before motor symptoms onset. Thus, the small but significant decrease in the expression TH mRNAs observed in SNc-DA neurons from G2019S mice might constitute an early marker of the disease in this mouse line.

Our electrophysiological analyses show that the firing and membrane properties of SNc-DA neurons are preserved in cNurr1 and G2019S mice. These results, obtained in brain slices, also demonstrate that DA and motor deficits of cNurr1 mice do not affect most basic membrane properties of SNc-DA neurons. Different ion channels underlie or facilitate SNc-DA neurons pacemaker firing, and its precision. Among these are the HCN channels and VGCCs. We found that non-selective Na^+^/K^+^ HCN channels underly the pacemaker firing of most SNc-DA neurons, and that this role is unchanged in cNurr1 and G2019S mice. Variable effects of the HCN channel blocker ZD 7288 were observed in previous studies and when this blocker reduced the pacemaker firing, this was observed in a subpopulation of SNc-DA neurons^35,36,58^. These discrepancies are likely due to differences in the experimental conditions, such as the concentration and duration of perfusion with the blocker, but most importantly the age of the animals used is a key factor that likely accounts for the observed differences. Together, these studies and our results from adult and middle-aged mice indicate that the contribution of HCN channels to the pacemaker firing increases with age. In addition, we found that the slow component of the AHP current following a long membrane depolarization was increased in SNc-DA neurons of cNurr1 mice. In several neuronal populations, the slow AHP controls spike firing adaptation and is mediated by small-conductance Ca^2+^-activated potassium (SK) channels^59^. SNc-DA neurons express SK3, a member of the SK channel family known to play a key role in regulating the firing pattern of these neurons^60^. However, in SNc-DA neurons, the slow AHP elicited by a long depolarization was shown to be insensitive to a SK channel blocker, apamin^61^, and is mediated by Ca^2+^-independent, voltage-dependent, potassium currents^62^. In accordance with these studies, the increased slow AHP amplitude in SNc-DA neurons of cNurr1 mice was not associated with an increased spike firing adaptation during trains of action potentials induced by positive current injections. Together, these observations demonstrate that SK channels do not play a role in the slow AHP in DA neurons in the mice used in this study, and under our experimental conditions. The increased slow AHP amplitude following long depolarization steps is therefore unlikely to be due to an increased SK channel function and might therefore not be dependent on Ca^2+^ influx. Nevertheless, the absence of alterations in the membrane properties, Ih current, pacemaker firing, action potential characteristics, excitability, fast AHP in SNc-DA neurons is remarkable given the age of G2019S mice and the severe neurochemical alterations and behavioral deficits in cNurr1 mice.

Our results support previous demonstrations that L-type VGCCs are not essential for the pacemaker firing in SNc-DA neurons^4,38^. Indeed, the L-type VGCC blocker isradipine did not affect the firing of these neurons in control and WT mice. Isradipine did not alter the pacemaker activity in cNurr1 mice, showing that the lack of contribution of Cav1.3 channels to the pacemaker firing is not altered. However, these channels play a necessary role and contribute to this autonomous activity in G2019S mice. This is associated with a reduction in Cav1.3 mRNAs in SNc-DA neurons. Together, these results suggest that an increased function of L-type VGCCs, and a reduced mRNA expression for these channels, characterize G2019S mice. Functional coupling between Cav1.3 L-type VGCCs and DA-D2 receptors was demonstrated in SNc-DA neurons and was suggested to contribute to PD pathology^38^. The increased contribution of L-type VGCCs to SNc-DA neurons pacemaker activity in G2019S mice would lead to an increased sensitization of somatic DA-D2 autoreceptors, but this is unlikely because we did not observe any increased membrane hyperpolarization during the perfusion with quinpirole applied at two concentrations.

Cav1.3 L-type VGCCs were shown to amplify the firing rate and bursts in SNc-DA neurons, and a recent study demonstrated that this occurs in neurons located in the lateral SNc^63,64^. In G2019S mice, an increased L-type channels function might lead to enhanced burst firing or an altered regularity of firing, but this was not observed in our recording conditions. The location of the recorded neurons in the SNc (medial or lateral), the age of the mice, the use of brain slices rather than in vivo recordings are factors that could influence the generation of bursting activity in DA neurons. Future studies examining the firing of DA neurons in the lateral SNc in vivo are needed to determine possible alterations in the firing pattern in relation to increased function of L-type channels in G2019S mice. In addition, an increased bursting activity in a population of SNc-DA neurons might cause an elevated dopamine release from axon terminals in the striatum. This increased release might be of benefit for the mice and might delay the onset of motor deficits, indeed, those appear only in aged mice. However, increased bursting might also lead to enhanced intracellular Ca^2+^ and contribute to cell loss. The L-type VGCC blocker isradipine was examined in preclinical and clinical studies for its ability to reduce mitochondrial oxidative stress and its potential neuroprotective effects in mice and in PD patients^8,9,65^. Although the conclusions of a phase III clinical trial with isradipine were negative^65^, a recent re-analysis of the phase II trial shows evidence that isradipine slows early-stage PD progression^66^. Our findings support the suggestion that targeting L-type VGCCs might have benefit in some PD patients, in particular before the occurrence of motor symptoms, but the implementation of such treatment strategy in the general population would be complicated or unlikely given the difficulty to identify patients susceptible to develop PD.

Our study demonstrates that the function of T-type VGCCs is altered in SNc-DA neurons of cNurr1 mice, but not G2019S mice. Using a pharmacological approach, we found a reduced contribution of T-type VGCCs to SNc-DA neurons pacemaker activity in cNurr1 mice. Using a physiological method, where hyperpolarization-depolarization steps were applied to disinhibit and activate T-type channels, we also found a reduced function of these channels, as indicated by a reduced spike frequency adaptation. Given that T-type channels contribute to the pacemaker firing in Ctrl mice, it is remarkable that the firing of SNc-DA neurons in cNurr1 mice is unaltered. This is likely due to compensatory mechanisms, such as contribution of other channels to replace the reduced T-type channel, that maintain proper firing activity. The reduced function of T-type channels in cNurr1 mice might be a protective mechanism engaged by SNc-DA neurons to maintain their somata by reducing the intracellular Ca^2+^ concentration during waves of Ca^2+^ resulting from the transient activation of T-type channels. Indeed, intracellular Ca^2+^ levels might exert feedback on VGCCs, as shown for both L-type and T-type channels^67^. In addition, the reduced function of T-type channels might lead to desensitization of somatic DA-D2 autoreceptors. In accord with this possibility, we found that DA-D2 autoreceptors desensitize more in SNc-DA neurons of cNurr1 compared with Ctrl mice. Desensitization was not associated with a decrease in the amounts of DA-D2 receptors and of NCS-1, but was reduced in the presence of kaempferol, indicating a link to oxidative stress. Desensitization of DA-D2 autoreceptors would cause a reduced autoinhibition of SNc-DA neurons firing by somatodendritically released dopamine through activation of inwardly rectifying potassium (GIRK) channels^48^. As a consequence, more dopamine would be released in the striatum from residual axon terminals, which would have beneficial effects. Recent studies indicate that T-type VGCCs might contribute to vulnerability of SNc-DA neurons to degenerative stressors^12^, and these channels are suggested as novel PD drug targets^68^. Indeed, T-type channel blockers efficiently counteract PD tremor-like behaviors in rodents^69^, and a selective T-type channel blocker is being examined in a clinical trial for the treatment of tremor associated with PD^70^. Moreover, it is believed that the beneficial effects of zonisamide against PD tremor and wearing off is largely mediated via antagonism of T-type VGCCs^71,72^. Our findings support the suggestion that targeting T-type VGCCs might provide benefit for PD patients.

We found that the altered role of L-type VGCC in SNc-DA neurons pacemaker firing in G2019S mice is due to an increased LRRK2 kinase activity. Indeed, L-type VGCCs are unaltered in G2019S mice in the presence of a LRRK2 kinase inhibitor, GSK2578215A. Given that LRRK2 kinase inhibitors are being investigated for the treatment of PD in clinical trials, our results provide mechanistic insights into the possible effect of these compounds in early stages of the disease. We also found that the functions of L-type VGCCs in G2019S mice and of T-type VGCCs and DA-D2 autoreceptors in cNurr1 mice were unaltered in the presence of kaempferol. Kaempferol is a flavonoid with antioxidant and neuroprotective activity. This compound reduces the production of reactive oxygen species and neuronal damage in different models^42,73^. Kaempferol was also shown to activate the mitochondrial Ca^2+^ uniporter (MCU)^42,74^. Ca^2+^ uptake by mitochondria through the MCU normally mitigates local increases in cytosolic Ca^2+^ concentration during pacemaker firing. Kaempferol might thus reduce cytosolic Ca^2+^ during the transient activation of T-type VGCCs. However, mitochondrial Ca^2+^ overload is suggested to lead to the generation of reactive oxygen species and cell death^75^. In addition, MCU inhibitors have neuroprotective properties in neurodegenerative diseases^76^, which is not in accord with the effect of an MCU activator. In cNurr1 mice, altered mitochondrial function, in particular oxidative phosphorylation, was suggested in a previous study^23^, and the LRRK2 G2019S mutation is associated mitochondrial dysfunctions and increased susceptibility to toxic environmental factors^55,77,78^. Our results with kaempferol, and the increased Nrf2 amounts in the striatum of cNurr1 mice, suggest that oxidative stress occurs in our two PD mouse models. Although we did not observe any significant change in the amounts of Nrf2 in G2019S mice, other oxidative stress markers, not examined in the present study, might be altered in these mice. Alternatively, oxidative stress might be observable only when G2019S mice are exposed to toxic environmental factors, as suggested earlier^77^. It is possible that the changes in the functions of T-type and L-type VGCCs, as well as of DA-D2 autoreceptors in SNc-DA neurons of cNurr1 and G2019S mice might not be a direct cause of oxidative stress but a consequence of this stress. Indeed, these functions are unaltered in the presence of kaempferol and a LRRK2 kinase inhibitor. Interestingly, two different VGCCs are affected in opposite directions in cNurr1 and G2019S mice. This suggests that distinct cellular mechanisms, which remain to be identified, mediate the altered VGCC function in these two mouse lines.

In contrast to the changes observed in SNc-DA neurons somata, the function of L-type and T-type VGCC was unaltered in the axon terminals of these neurons. The frequency-dependence of dopamine release was not altered in G2019S mice, but we found a small facilitation of dopamine release evoked by 15 Hz trains in cNurr1 mice, instead of a depression. This facilitation might be due to a decreased inhibitory control by dopamine released by the first pulse in the train, given that the amounts of dopamine are drastically reduced in cNurr1 mice. This reduction in striatal dopamine is likely due to the decreased expression of TH and DAT in the striatum of cNurr1 mice. Downregulation of DA-D2 autoreceptors might also cause this facilitation. No increased facilitation was observed at 100 Hz trains which might indicate lower DA-D2 receptor autoregulation of dopamine released by high frequency trains. Our results show that isradipine and NNC 55-0396 inhibit dopamine release in the dorsolateral striatum. This agrees with a previous study which additionally demonstrated that the inhibitory effects of these compounds on dopamine release were observed in the presence of GABA and glutamate receptor blockers, ruling out the involvement of VGCCs in, e.g., GABAergic striatal interneurons and glutamatergic axon terminals^50^. Moreover, isradipine inhibited dopamine release induced by optogenetic stimulation of DA axons^50^. It is therefore likely that L-type and T-type VGCCs are present at DA axon terminals in the dorsal striatum where they regulate dopamine release. Interestingly, isradipine and NNC 55-0396 inhibited dopamine release in the dorsal striatum, which is mostly innervated by DA neurons in the SNc and had no effect on dopamine release in the ventral striatum which is mostly innervated by DA neurons in the ventral tegmental area^50^. L-type and T-type channels might thus play a role in the susceptibility of SNc-DA neurons to degeneration in PD. Our results further demonstrate that the functions of these channels are unaltered in the residual terminals of cNurr1 mice as well as in the terminals that have not degenerated yet in G2019S mice. It is therefore possible that axon terminals do not engage similar protective mechanisms as seen in their somata, which could contribute to their early loss in PD.

Our study has some limitations. First, we did not examine SNc-DA neurons in the same mouse line before the development of motor and neurochemical impairments and when these deficits occur, which would have allowed us to associate neurophysiological dysfunctions with presymptomatic vs. symptomatic Parkinsonism. Indeed, cNurr1 mice start developing DA deficits as early as one week following tamoxifen injections^23^, and G2019S mice start demonstrating motor and neurochemical impairments at an old age, which makes electrophysiological investigations difficult to conduct^30^. Second, we did not perform in vivo electrophysiological recordings of the activity of DA neurons to investigate if changes in VGCCs functions correlated with an altered the firing pattern. Third, we have shown that oxidative stress and increased LRRK2 kinase activity contribute to the altered VGCC function, and desensitization of DA-D2 autoreceptors, but additional mechanistic insight into these changes in relation to the function of Nurr1 and LRRK2 need further investigations.

In conclusion, we have identified alterations in VGCCs function, which occur in the somata, but not in the axon terminals, of SNc-DA neurons of cNurr1 and G2019S mice and are associated with different stages of the development of motor and neurochemical impairments. Thus, SNc-DA neurons of different PD models might engage different protective mechanisms that counteract PD triggers and might delay degeneration of their somata. Our results provide insight into the possibilities for development of therapies or tools that maintain DA neurons somata and prevent further loss of their axons in PD.

## Methods

### Animals

Animal experiments were approved by our local ethical committee (Stockholms norra djurförsöksetiska nämnd, 20464-2020). Mice were bred in our animal facility. *Nurr1* floxed mice and mice harboring a tamoxifen-inducible Cre (*CreER*^*T2*^) linked to the dopamine transporter gene regulatory sequences in a bacterial artificial chromosome (*BAC-DAT-CreER*^*T2*^ mice) were generated as described previously^23,79^. Crosses between these transgenic lines facilitates inducible *Nurr1* gene ablation exclusively in DA neurons. Littermates homozygous for *Nurr1* floxed allele harboring no copy of the *BAC-DAT-CreER*^*T2*^ transgene served as controls (Ctrl). Littermates homozygous for *Nurr1* floxed allele harboring a single copy of the *BAC-DAT-CreER*^*T2*^ transgene served as conditional cNurr1^DATCreER^ knockout mice (cNurr1). All mice, both CreER positive (cNurr1 mice) and negative (Ctrl mice), were injected with tamoxifen (Tmxf, 2 mg, i.p., 9:1 sunflower seed oil/ethanol; Sigma-Aldrich; Fig. 1A) daily for 5 consecutive days starting at postnatal day 35 (P35). This treatment induced *Nurr1* ablation in DA neurons in cNurr1 mice but not in Ctrl mice^23^ (Supplemental Fig. 1A). We used male and female 6–8-month-old cNurr1 and Ctrl mice. We also used 10–12-month-old mice in the experiments presented in Supplemental Fig. 1D and Supplemental Fig. 7. Behavioral experiments were performed in the afternoon (12.00–16.00). For the other experiments, mice were sacrificed in the morning (10.00–12.00).

Mice which express the G2019S mutation in the human *LRRK2* gene were generated via BAC transgenesis. These mice were obtained from The Jackson laboratory (C57BL/6J-Tg(LRRK2-G2019S)2AMjff/J, JAX stock #018785; RRID:IMSR_JAX:018785) and were mated as Noncarrier x Hemizygote^30,32^. We used 10–12-month-old, male and female, hemizygous mice (G2019S) and non-transgenic wildtype (WT) littermates. We also used 8-month-old mice in the experiments presented in Supplemental Fig. 1B, D. All studied mice were backcrossed on a C57BL/6 J genetic background. Mice were housed in small groups (2–5 per cage, IVC Mouse—GM500) in a humidity-controlled room with a 12:12 h light/dark cycle and had free access to food and water.

### Behavioral test: Pole test

We assessed fine motor behavior by performing the Pole test. Mice were allowed to adapt to the testing room for at least 30 min before the test was conducted. Mice were placed individually on top of a vertical pole (diameter: 8 mm, height: 50 cm) with their head facing upwards. During the first 2 days of the test, mice were trained to turn and descend the pole back into a cage. On the third day of the test, mice were videotaped while descending the pole for a total of three trials. The time taken by the mice to turn downward (Tturn) and the total time to descend the pole (Ttotal) were measured manually with a timer. Data represent the average of three trials.

### Electrophysiology and amperometry in brain slices

Mice were deeply anesthetized with isoflurane and underwent transcardiac perfusion with 60 ml ice-cold oxygenated (95% O_2_ + 5% CO_2_) artificial cerebrospinal fluid (aCSF) containing (in mM): NaCl (126), KCl (2.5), NaH_2_PO_4_ (1.2), MgCl_2_ (1.3), CaCl_2_ (2.4), glucose (10) and NaHCO_3_ (26). Their brains were rapidly removed and submerged in a slicing solution containing (in mM): NaCl (15.9), KCl (2), NaH_2_PO_4_ (1), Sucrose (219.7), MgCl_2_ (5.2), CaCl_2_ (1.1), glucose (10) and NaHCO_3_ (26). Coronal hemisections (200–250 μm thick) containing the midbrain and the striatum were obtained using a microslicer (VT 1000 S, Leica Microsystem, Heppenheim, Germany). The sections were incubated in a modified aCSF containing (in mM): NaCl (126), KCl (2.5), NaH_2_PO_4_ (1.2), MgCl_2_ (4.7), CaCl_2_ (1), glucose (10) and NaHCO_3_ (23.4) at 32 °C for 1 h following the slicing and afterwards at 28 °C. We performed somatic cell-attached and whole-cell patch-clamp recordings of visually identified SNc-DA neurons, as described previously^32,80^. Neurons were identified as being DA based on several anatomical, morphological, and electrophysiological criteria which included the location of these neurons in the SNc, their slow spontaneous firing (<6 Hz), the presence of an Ih current and membrane capacitance >40 pF which characterize DA neurons. Cells that did not meet these criteria were not further recorded and were not included in the analyses. These criteria allowed us to differentiate between DA neurons and non-DA neurons, and our previous studies confirmed that the recorded neurons contained tyrosine hydroxylase (TH)^80^. Patch electrodes (3–5 MΩ) were filled with a solution containing (in mM): D-gluconic acid potassium salt (120), KCl (20), HEPES (10), EGTA (10), MgCl_2_ (2), CaCl_2_ (1), ATP-Mg (2), GTPNa_3_ (0.3), pH adjusted to 7.3 with KOH. Recordings were performed with a MultiClamp 700B (Axon Instruments, Foster City CA, USA), acquired at 10 kHz and filtered at 2 kHz. Spontaneous firing of DA neurons was measured with tight seal (>500 MΩ, monitored during the recording) somatic cell-attached recordings at 0 mV. Amperometric detection of DA release was performed, as described previously^81,82^, with carbon fiber electrodes (10 μm diameter, World Precision Instruments Europe) which had an active part of 100 μm that was positioned within the dorsolateral part of the striatum in the brain slice. A constant voltage of + 500 mV was applied to the carbon fiber via an Axopatch 200B amplifier (Axon Instruments) and currents were recorded with the same amplifier. A stimulating electrode (patch electrode filled with aCSF) was placed on the slice surface, in the vicinity of the carbon fiber electrode. Stimulations evoked a response corresponding to oxidation of dopamine at the surface of the carbon fiber electrode. Stimulations consisted of either single pulses or trains of 4 pulses at 15 Hz and at 100 Hz (pulse duration 0.2 ms; intensity 15–20 μA) and were applied every minute (single) and every 2 min (trains). Data were acquired and analyzed with the pClamp 10 software (Axon Instruments, Foster City CA, USA).

### Western blotting

Brain slices were prepared as described for slice electrophysiology. The striatum and midbrain were dissected from the slices, frozen and stored at −80 °C until processed. The samples were sonicated in 1% sodium dodecyl sulfate (SDS) and boiled for 10 min. 1% SDS was diluted in water from 10% SDS (prepared with 18 MΩ water, Bio-Rad, Cat. No. 1610416). Protein concentration was determined in each sample with a bicinchoninic acid protein assay (BCA-kit, Pierce, Rockford, US, Cat. No. 23225). Equal amounts of protein (15 μg for midbrain and 30 μg for striatum) were re-suspended in sample buffer (4 × Laemmli Sample Buffer, Bio-Rad, Hercules, USA, Cat. No. 1610747, added with 10% β-mercaptoethanol, Sigma, St Louis, USA, Cat. No. M3148) and separated by SDS–polyacrylamide gel electrophoresis using a 9% or 12% acrylamide gel (Acrylamide/Bis-acrylamide, 30% solution: Sigma, St Louis, USA, Cat. No. A3699) and transferred to a nitrocellulose transfer membrane (Bio-Rad, Hercules, USA, Cat. No. 1620115). The membranes were incubated for 1 h at room temperature with 5% (w/v) fat-free dry milk (Cell Signaling, Danvers, USA, Cat. No. 9999 S) in TBS-T (Tris base 0.05 mol/L, NaCl 0.15 mol/L, tween 0.1%). Immunoblotting was carried out with primary antibodies in 5% dry milk dissolved in TBS-T at 4 °C overnight. Antibodies were obtained from Sigma-Aldrich, St Louis, USA (TH, Cat. No. T2928 or A11004, dilution 1:2000; β-actin, Cat. No. A2228, dilution 1:2000); Millipore, Temecula or Billerica, USA (D2R, Cat. No. AB50884P, dilution 1:1000; DAT, Cat. No. MAB369, dilution 1:1000); Abcam, Cambridge, UK (VMAT2, Cat. No. ab191121, dilution 1:500; NCS-1, Cat. No. ab129166, dilution 1:1000); and Thermo Scientific, Rockford, USA (Nrf2, Cat. No. PA5-27882, dilution 1:1000). The membranes were washed three times with TBS-T and incubated, for 1 h at room temperature, with secondary horseradish peroxidase-linked Anti-Rabbit IgG (H + L) (Thermo Scientific, Rockford, USA, Cat. No. 32260, 1:5000 dilution) or Anti-Mouse IgG (H + L) (Thermo Scientific, Cat. No. 32230, Rockford, US, 1:5000 dilution). At the end of the incubation, membranes were washed six times with TBS-T and immunoreactive bands were detected by enhanced chemiluminescence (Bio-Rad, Hercules, USA, Cat. No. 170-5061). The membranes were then scanned in ChemiDoc MP system (Bio-Rad, Hercules, USA) and quantified with ImageJ 1.50b software (NIH, USA). The protein amounts were normalized to the value of β-actin and expressed as a percentage of the averaged value obtained for WT or Ctrl mice. All blots were processed in parallel and derive from the same experiment.

### Analysis of neurotransmitters by high-performance liquid chromatography (HPLC)

HPLC with electrochemical detection (ECD) was done according to previously published protocols^83,84^. The striatum was dissected from fresh brain hemispheres and placed in Eppendorf tubes which were weighed beforehand and then stored at −80 °C until use. Total weight of both tissue and Eppendorf tube was measured before use and tissue weight was calculated by subtracting Eppendorf weight from total weight. Ice-cold 0.1 M perchloric acid was added to tissue sample. Samples were sonicated and incubated on ice for 10 min, vortexed and centrifuged at 16,000 x *g* for 15 min at 4 °C. Resulting supernatants were filtered through 0.2 µm nylon membrane inserts and centrifuged at 5000 x *g* for 5 min. Eluents were immediately stored at −80 °C until subjected to HPLC-ECD analysis. Standard solutions of dopamine hydrochloride, 3,4-dihydroxyphenylacetic acid (DOPAC), homovanillic acid (HVA) and 3-Methoxytyramine hydrochloride (3-MT) were prepared in 0.1 M perchloric acid to obtain final standard concentrations of 200, 100, 50, 10, 5, 2 and 1 ng/ml. Calibration curves were obtained with the Chromeleon software through linear regression of peak area versus concentration. The HPLC-ECD system used was a Dionex Ultimate 3000 series (Dionex, ThermoFisher Scientific, USA). Analyte separation was performed on a Dionex C18 reversed-phase MD-150 3.2 mm × 250 mm column (3 µm particle size). Column and analytical cell were kept at 30 °C. The mobile phase, which was pumped at a flow rate of 0.4 ml/min, consisted of 75 mM monobasic sodium phosphate, 2.2 mM 1-octanesulfonic acid sodium salt, 100 µl/l triethylamine, 25 µM ethylene-diamine-tetra-acetic acid disodium salt and 10 % acetonitrile (v/v), pH 3.0 adjusted with 85% phosphoric acid. For detection of dopamine and its metabolites, the first and second analytical cells were set to −100 mV and +300 mV, respectively. Processed tissue samples were thawed on ice in the dark for about 1 h before analysis, placed in the autosampler and kept at 5 °C before injection. Chromatograms were acquired with Dionex Chromeleon 7 software over an acquisition time of 55 min. Analyte concentrations in tissue samples were expressed as ng/mg of tissue.

### Immunofluorescence and cell count

Mice underwent transcardiac perfusion with saline followed by 4% paraformaldehyde (Sigma-Aldrich, St Louis, USA, Cat. No. 16005) in phosphate buffer saline (PBS; Sigma-Aldrich, St Louis, USA, Cat. No. P4417) under deep isoflurane anesthesia. Their brain was removed, post-fixated in 4% paraformaldehyde overnight and dehydrated in 30% sucrose-PBS buffer for 2–3 days. Dehydrated brains were embedded in OCT cryomount (Cat. No. 45830, Histolab, Gothenburg, Sweden), frozen at −20 °C and sliced with a MICROM cryostat (HM 500 M) at a 40 μm thickness. The sections were collected and stored in NaN_3_ (0.01% in PBS) in 24-well plates at 4 °C. Free floating brain sections containing the midbrain were incubated for one night at 4 °C in a TH primary antibody (Sigma-Aldrich, St Louis, USA, T2928, dilution 1:4000 or Millipore, Temecula, USA, Cat. No. AB152, dilution 1:2000). Sections were washed 3 times in PBS and incubated in Alexa Fluor® 568-conjugated goat anti-rabbit-IgG (Thermo Scientific, Rockford, USA, Cat. No. A-11011, dilution 1:2000), Alexa Fluor® 488-conjugated goat anti-rabbit-IgG (A-11004, Thermo Scientific, Rockford, US, dilution 1:2000) or Alexa Fluor® 488-conjugated goat anti-mouse-IgG (Thermo Scientific, Rockford, USA, Cat. No. A-11001, dilution 1:2000) for 2 h at room temperature, followed by re-washing in PBS and mounting with 70% glycerol. The sections were imaged on a Carl Zeiss LSM 880 confocal microscope (Oberkochen, Germany) using a 20x or a 63x oil objective. Images were z-stacked. For counting the number of TH-positive cells, TH immunostaining was performed in 4-5 sections containing different antero-posterior (Bregma -4.80 - 6.04) regions of the SNc for each mouse examined. After confocal scanning with a 20x objective, the number of TH-positive cells in the SNc in both sides of each section was counted manually using Cell Counter plugin in Fiji^85^ and a surface cell count method described earlier^86,87^. The number of cells from the two sides of the same section were added, and the average of all sections was calculated for each mouse.

### Neuronal morphology and Sholl analysis

Patch electrodes made of borosilicate glass were filled with intracellular recording solution containing 0.2% Neurobiotin Tracer (Vector Laboratories, Burlingame, US). After patch clamp recording, the slices with neurobiotin-filled cells were fixed overnight in 4% paraformaldehyde at 4 °C. The slices were washed 5 min 3 times with PBS. Cell membrane permeabilization was achieved by 1 h incubation in PBS-based buffer containing 10% Triton. The slices were then washed with PBS, incubated with Alexa Fluor® 488-conjugated streptavidin (Jackson ImmunoResearch, West Groove PA, US, 1:750) for 1 h. After washing, slices were mounted on slides, dried, and covered with cover slips with 70% glycerol. Confocal images of neurobiotin-containing neurons were obtained with a Carl Zeiss LSM 880 confocal microscope using a 63 × 1.4 NA oil immersion objective and processed by Fiji^85^. Neuron morphology reconstruction and analysis were performed by Simple Neurite Tracer and Sholl Analysis plugins^88^.

### In situ hybridization

In situ hybridization was performed in cryostat (CM 3050 S, Leica) fresh frozen thaw-mounted sections (12 μm thick) as previously described^89^. Briefly, ^35^S-labeled anti-sense cRNA probes were prepared by in vitro transcription from DNA corresponding to fragments of Nurr1. The transcription was performed from 50 to 100 ng of linear DNA using [^35^S] UTP (1000 Ci/mmol) and T3 polymerase. Cryostat sections were post fixed in 4% paraformaldehyde for 5 min at room temperature, rinsed twice in 4 × sodium chloride–sodium citrate buffer (SSC) and placed into 0.25% acetic anhydride in 0.1 M triethanolamine/4 × SSC (pH 8) for 10 min at room temperature. After dehydration in graded alcohols, the sections were hybridized overnight at 55 °C with ^35^S-labeled probe in 50 μl of hybridization solution (20 mM Tris–HCl/1 mM EDTA/300 mM NaCl/50% formamide/10% dextran sulphate/1 × Denhardt’s/250 μg/ml yeast tRNA/100 μg/ml salmon sperm DNA/0.1% SDS/0.1% sodium thiosulphate). The slides were washed in 4 × SSC (5 min, four times), RNAse A (20 μg/ml) (20 min, at 37 °C), 2 × SSC (5 min, twice), 1 × SSC (5 min), 0.5 × SSC (5 min) at room temperature and rinsed in 0.1 × SSC at 65 °C (30 min, twice). The slides were washed once 0.1 × SSC (5 min) at room temperature before being dehydrated in graded alcohols. The slides were then exposed on X-ray films for 1 week.

### Fluorescent in situ hybridization (FISH)

FISH (RNAscope^®^) was performed using the RNAscope^®^ Multiplex Fluorescent Assay (Advanced Cell Diagnostics, Cat. No 320850). Cryostat (Leica CM 3050 S) fresh-frozen thaw mounted sections (12 μm thick) were post-fixated with 4% paraformaldehyde (dissolved in 1× PBS). The slides were dehydrated with graded ethanol solutions (75%, 80%, 90%, 95% and 100%). Protease IV Reagent (Advanced Cell Diagnostics) was then applied for 30 min at room temperature. Afterwards, the sections were hybridized for 2 h at 40 °C with the following probes: TH (Cat No. 317621-C2), CACNA1D (Cav1.3, Cat No. 502591), CACNA1G (Cav3.1, Cat No. 459761). The hybridization step was followed by standardized steps of amplification (Amp 1-FL 30 min at 40 °C, Amp 2-FL 15 min at 40 °C, Amp 3-FL 30 min at 40 °C, Amp 4C-FL 15 min at 40 °C). The last amplification step was followed by DAPI (Advanced Cell Diagnostics) counterstaining and mounting with Dako fluorescent mounting medium. The brain sections were imaged on a Carl Zeiss LSM 880 confocal microscope using a 63 × 1.4 NA oil immersion objective. Z-stacks of 5-10 µm thickness were obtained in each caption. Quantification of mRNA was done by measuring fluorescence within individual cells using FIJI software. Data are expressed as a percentage of Ctrl or WT.

### Chemicals and drugs

Salts and other chemicals were purchased from Sigma-Aldrich (Stockholm, Sweden), Tocris/Bio-Techne Ltd. (Abingdon, UK) and Hello Bio (Bristol, UK). The compounds used for slice electrophysiology were prepared in stock solutions, diluted in aCSF to their final concentration, and applied in the perfusion solution. The following compounds were used (final concentrations in μM): ZD 7288 (50), quinpirole (0.1 and 10), isradipine (0.2), NNC 55-0396 dihydrochloride (10), kaempferol (5), dihydro-β-erythroidine hydrobromide (DhβE, 0.1), GSK2578215A (1). The ion channel blockers (ZD 7288, isradipine and NNC 55-0396) were applied in the perfusion solution. Evoked-dopamine release was measured during the last 5 min of 35 min drug perfusion and expressed as percentage of the value measured before the start of the perfusion. The firing of SNc-DA neurons was measured for 1 min of stable baseline before the start of drug application, and the last minute of 30 min drug perfusion. In the experiments using kaempferol and GSK2578215A, slices were pre-incubated in aCSF containing kaempferol (5 μM) for at least 3 h, or GSK2578215A (1 μM) for at least 2 h^90^, before being placed in the recording chamber.

### Statistical analysis

The GraphPad Prism 9 software was used for data analysis and statistics. Data are expressed as mean ± s.e.m with *n* and *N* indicating the number of neurons, or slices, and mice tested. We used the Shapiro-Wilk test to assess normal distribution of the data. Statistical significance of the results was assessed by using the Student’s *t*-test for paired and unpaired observations, or one-way or two-way ANOVA followed by Dunnett’s or Tukey multiple comparison test when datasets fulfill normal distribution. Mann–Whitney *U*-test, Wilcoxon test, or Kruskal-Wallis tests were used when non-normal distributed datasets were tested. All tests were two-tailed. Significant levels were set at *P* < 0.05.

### Reporting summary

Further information on research design is available in the Nature Portfolio Reporting Summary linked to this article.

## Supplementary information

Supplemental Information

Western blots

Reporting summary

## Data Availability

The datasets generated and/or analyzed during the current study are available from the corresponding author, and will be shared, on request.
