# Supplementary material for: Aberrant somatic calcium channel function in cNurr1 and LRRK2-G2019S mice
Source: NPJ Parkinsons Dis. 2023 Apr 7;9:56. doi: 10.1038/s41531-023-00500-5 (PMC10082048; doi:10.1038/s41531-023-00500-5)
Supplement: Supplementary file 1 — Supplemental Information [file 41531_2023_500_MOESM1_ESM.pdf]

Supplementary Information

Aberrant somatic calcium channel function in cNurr1 and LRRK2-G2019S mice

Olga Skiteva,[1] Ning Yao,[1] Ioannis Mantas,[2] Xiaoqun Zhang,[2] Thomas Perlmann,[3] Per Svenningsson,[2] and Karima Chergui[1,*]

[1] Department of Physiology and Pharmacology, Karolinska Institutet, Stockholm, Sweden

[2] Department of Clinical Neuroscience, Karolinska Institutet, Stockholm, Sweden

[3] Department of Cell and Molecular Biology, Karolinska Institutet, Stockholm, Sweden

*Corresponding author: karima.chergui@ki.se

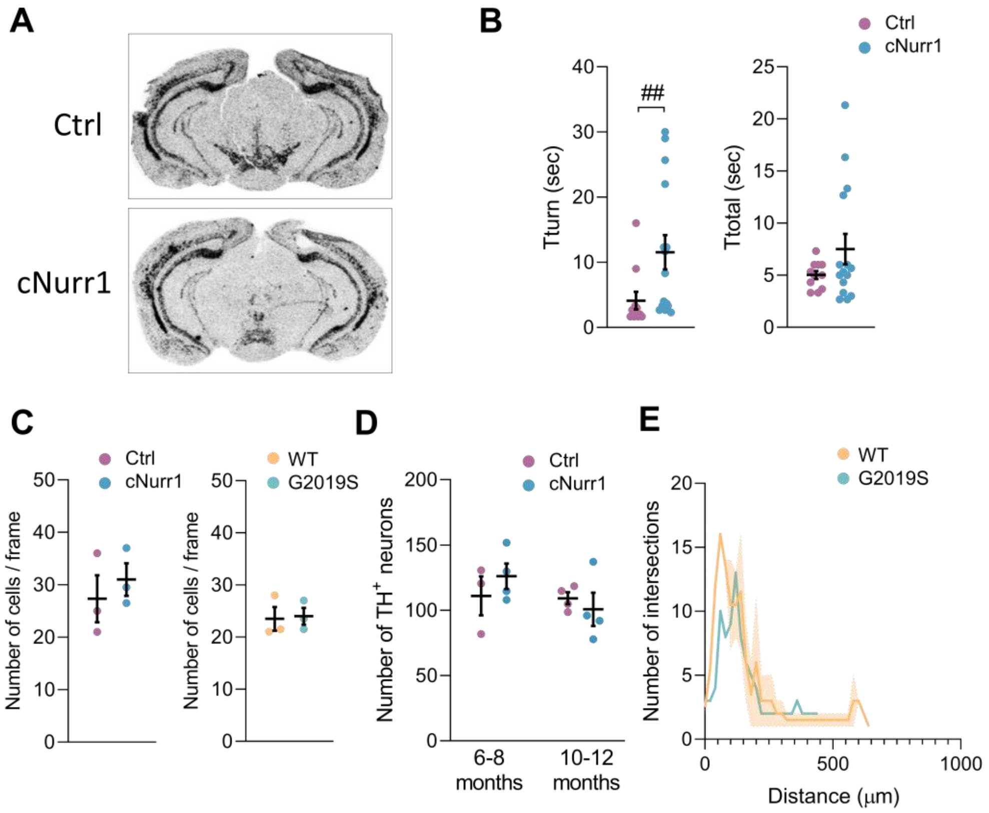

Supplemental Figure 1: Characterization of cNurr1 and G2019S mice. (**A**) *In situ* hybridization images illustrating the presence of Nurr1 mRNA in the SNc of a Ctrl mouse and its absence in a cNurr1 mouse. (**B**) Fine motor coordination was assessed with the pole test in middle-aged (10-12 months) Ctrl and cNurr1 mice. Tturn: time taken by the mice to turn downward from the top of a vertical pole; Ttotal: total time to descend the pole. N = 11 Ctrl and 15 cNurr1 mice. $^{\#\#}P < 0.01$; Mann-Whitney U test. (**C**) Number TH-positive neurons, counted from our FISH experiments, in the SNc of 6-8-month-old Ctrl and cNurr1 mice and 10–12-month-old WT and G2019S mice (N = 3 mice in each group). (**D**) Number of TH-positive neurons in the SNc of middle-aged (10-12 months) Ctrl and cNurr1 mice counted from our immunofluorescence experiments in N = 4 Ctrl and 4 cNurr1 mice. Data from 6-8-month-old Ctrl and cNurr1 mice (from Fig. 2) are included in the graph for comparison. (**E**) Sholl analysis shows the number of intersections measured in neurobiotin-filled neurons in the SNc of adult (8 months) WT (N = 2) and G2019S (N = 1) mice.

	cNurr1 Ctrl aCSF	cNurr1 Ctrl KF	cNurr1 KO aCSF	cNurr1 KO KF	LRRK2 WT aCSF	LRRK2 WT KF	LRRK2 G2019S aCSF	LRRK2 G2019S KF
Frequency (Hz)	2.87 ± 0.24	3.17 ± 0.23	2.56 ± 0.27	2.86 ± 0.25	2.82 ± 0.16	2.71 ± 0.16	2.77 ± 0.18	3.08 ± 0.27
	n=26	n=18	n=27	n=18	n=32	n=30	n=26	n=20
CV (%)	5.71 ± 0.58	6.56 ± 1.12	7.04 ± 0.66	8.45 ± 1.42	5.04 ± 0.46	5.41 ± 0.46	5.82 ± 0.35	6.41 ± 0.68
	n=26	n=18	n=27	n=18	n=32	n=30	n=26	n=20
Cm (pF)	70.8 ± 3.46	75.9 ± 4.74	69.64 ± 2.95	67.86 ± 4.53	65.93 ± 2.45	59.29 ± 3.58	65.48 ± 2.75	69.46 ± 7.28
	n=30	n=10	n=25	n=14	n=27	n=7	n=46	n=13
Ri (MΩ)	249.8 ± 33.0	250.58 ± 58.9	246.0 ± 24.2	260.6 ± 36.2	254.6 ± 26.5	265.7 ± 51.9	220.2 ± 17.0	311.5 ± 64.9
	n=30	n=10	n=25	n=14	n=27	n=7	n=46	n=13

Supplemental Table 1: Firing and intrinsic membrane properties of SNc-DA neurons are unaltered in cNurr1 and G2019S mice. The pacemaker activity (action potential firing frequency in Hz and coefficient of variation of interspike intervals in %) of SNc-DA neurons was recorded in the cell-attached mode. Membrane capacitance (Cm, pF) and input resistance (Ri, MΩ) were measured in the whole-cell configuration. *n* indicates the number of SNc-DA neurons examined. aCSF: control conditions; KF: slices incubated in kaempferol. No statistically significant differences were observed between the different groups (Unpaired Student's *t*-test).

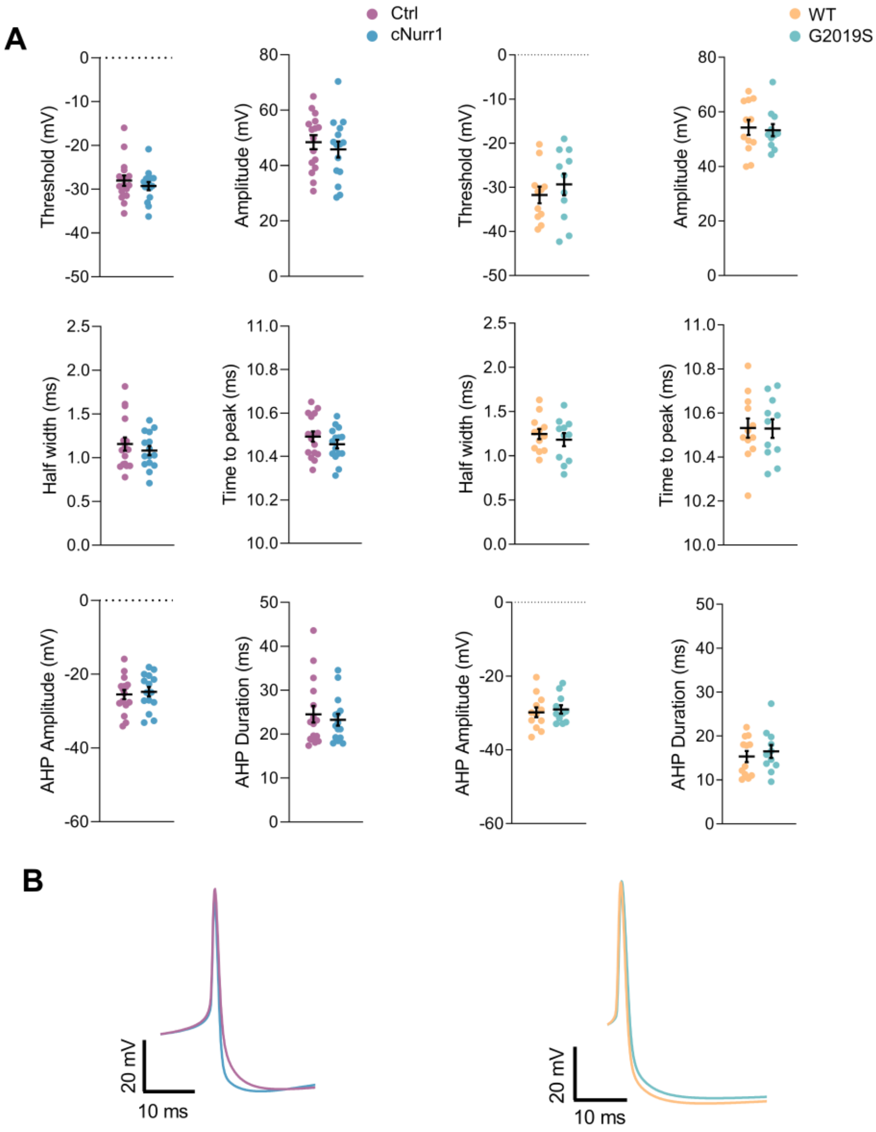

Supplemental Figure 2: Action potential characteristics are unaltered in SNc-DA neurons of cNurr1 and G2019S mice. (**A**) Action potential threshold, amplitude, half width, time to peak and after hyperpolarization (AHP) amplitude and duration were measured during pacemaker firing in SNc-DA neurons recorded in the whole-cell current-clamp configuration. n = 16, 15, 11, 11 neurons from N = 8 Ctrl, 7 cNurr1, 6 WT and 5 G2019S mice. (**B**) Superimposed representative traces of single action potentials measured in four SNc-DA neurons from Ctrl, cNurr1, WT and G2019S mice.

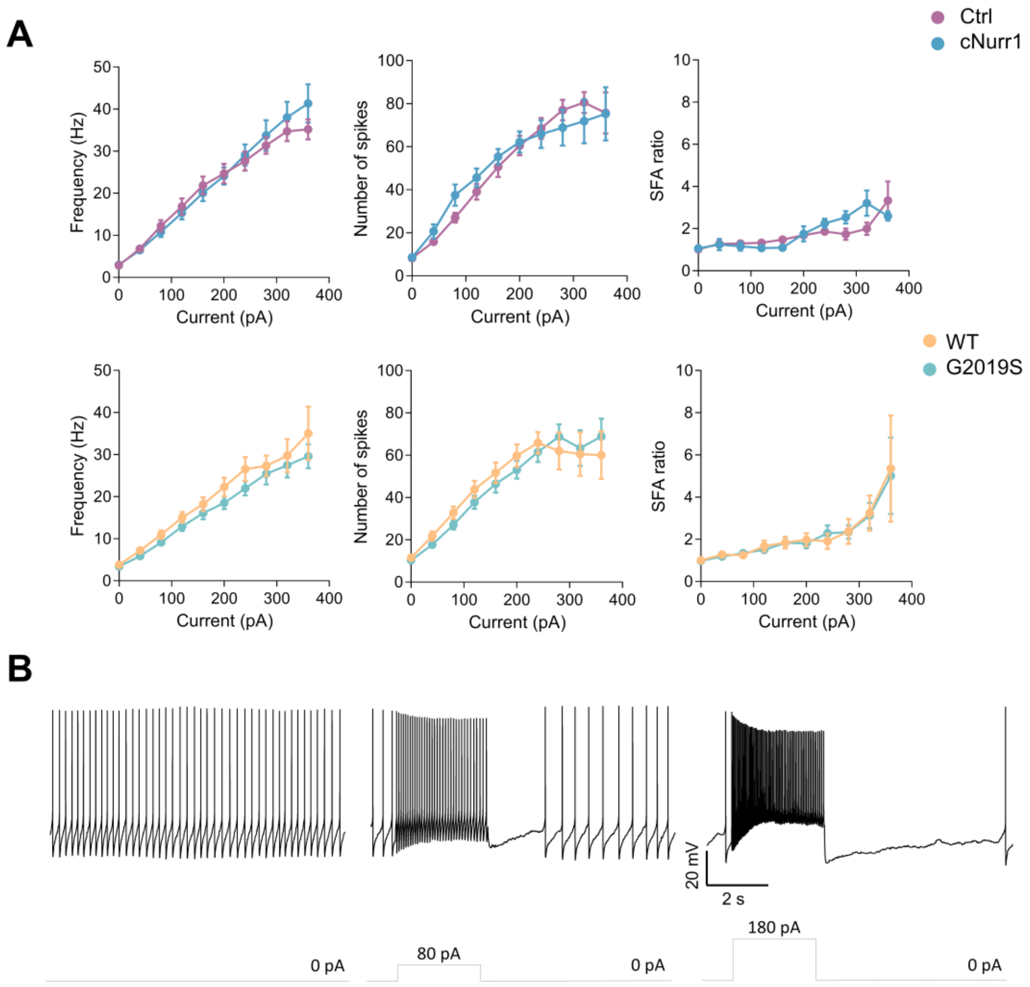

Supplemental Figure 3: Excitability of SNc-DA neurons is unaltered in cNurr1 and G2019S mice. (**A**) Graphs show action potential firing frequency, number of action potentials (spikes) and spike firing adaptation (SFA) ratio (ratio of the interspike interval between the last two action potentials and that of the first two action potentials) in response to increasing current steps and recorded in the whole-cell current-clamp configuration. *n* = 8, 10, 9, 9 neurons from *N* = 2 Ctrl, 6 cNurr1, 4 WT and 4 G2019S mice. (**B**) Representative current-clamp recordings of a SNc-DA neuron firing autonomously (pacemaker) with no current injection (0 pA) and firing evoked by +80 pA and +180 pA current injections from 0 pA.

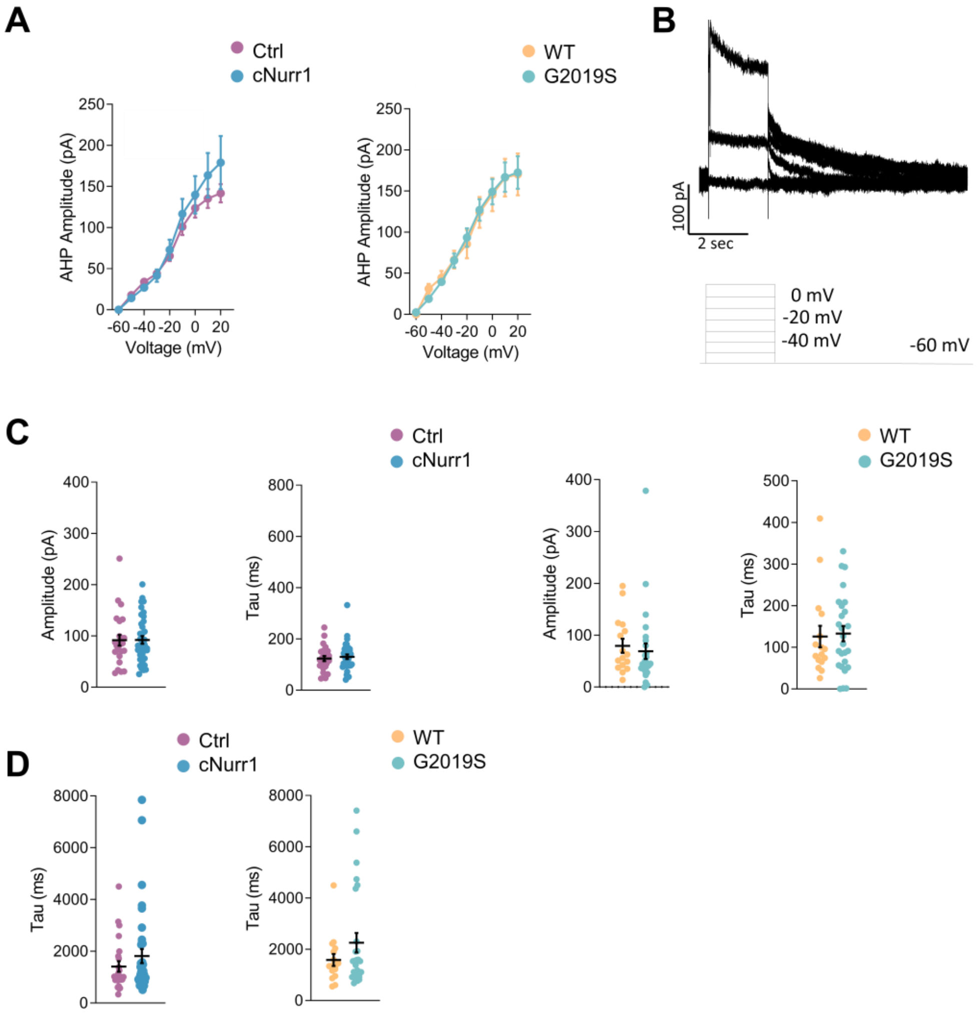

Supplemental Figure 4: AHP and fast component AHP following long positive voltage steps are not altered in cNurr1 and G2019S mice. (**A**) Graphs show the amplitude of the AHP measured following increasing depolarizing voltage steps (2 sec long) from a holding potential of -60 mV. *n* = 8, 10, 9, 9 neurons from *N* = 2 Ctrl, 6 cNurr1, 4 WT and 4 G2019S mice. (**B**) Representative voltage-clamp recordings showing currents induced by depolarizing voltage steps of increasing amplitude from a holding potential of -60 mV (bottom). Currents evoked by voltage steps above -40 mV are not shown to emphasize the AHP currents. (**C**) Graphs show fast AHP amplitude and Tau measured in *n* = 24, 37, 16, 26 SNc-DA neurons from *N* = 9 Ctrl, 11 cNurr1, 5 WT and 8 G2019S mice. (**D**) Graph shows slow AHP Tau measured in *n* = 24, 37, 16, 26 SNc-DA neurons from *N* = 9 Ctrl, 11 cNurr1, 5 WT and 8 G2019S mice.

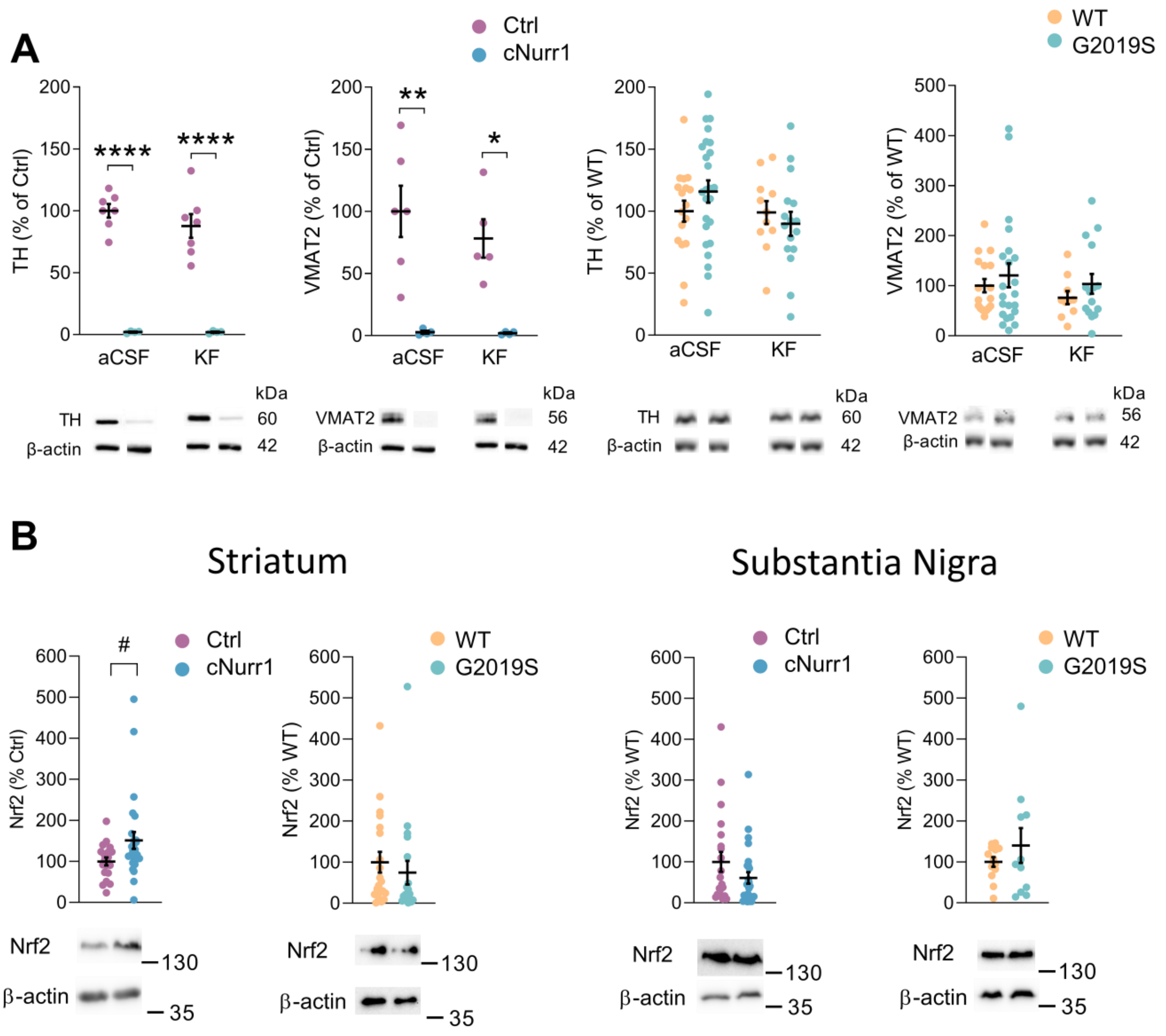

Supplemental Figure 5: Lack of effect of kaempferol on the amounts of striatal DA markers and increased Nrf2 amounts in the striatum of cNurr1 mice. (**A**) Western blotting of TH and the vesicular monoamine transporter 2 (VMAT2) from striatal slices incubated in aCSF and kaempferol (KF). $N = 7$ Ctrl, 4 cNurr1, 14 WT and 25 G2019S mice. Data for TH from the aCSF group of LRRK2/WT mice are the same as those shown in Fig. 1 and are included in the graphs for comparison. $^{*}P < 0.05$; $^{**}P < 0.01$; $^{****}P < 0.0001$; Two-way ANOVA followed by multiple comparisons (Tukey). (**B**) Western blotting of Nrf2 in the striatum of $N = 19$ Ctrl, 26 cNurr1, 20 WT and 19 G2019S mice, and in the substantia nigra of $N = 21$ Ctrl, 26 cNurr1, 13 WT and 11 G2019S mice $^{\#}P < 0.05$; Mann-Whitney U test.

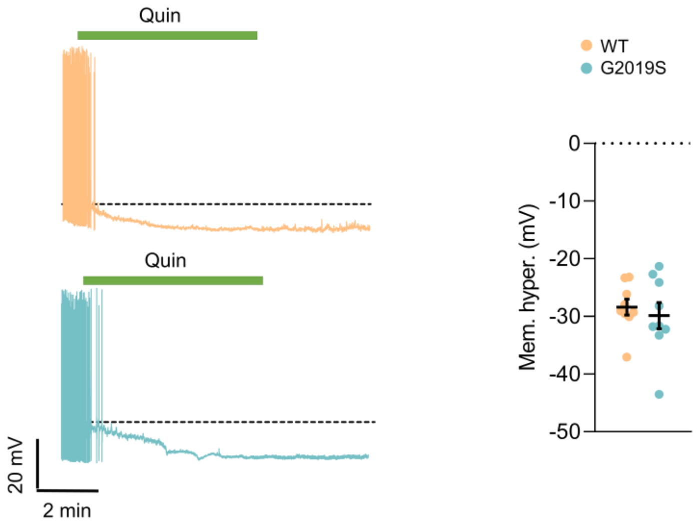

Supplemental Figure 6: A low concentration of quinpirole produces membrane hyperpolarizations of similar amplitude in WT and G2019S mice. Representative current-clamp recordings of the pacemaker firing measured at resting membrane potential (dotted lines) before, during and after the perfusion with quinpirole (100 nM, 5 min). Graphs show the amplitude of the quinpirole-induced membrane hyperpolarization. *n* = 9 and 9 neurons from *N* = 4 and 5 WT and G2019S mice.

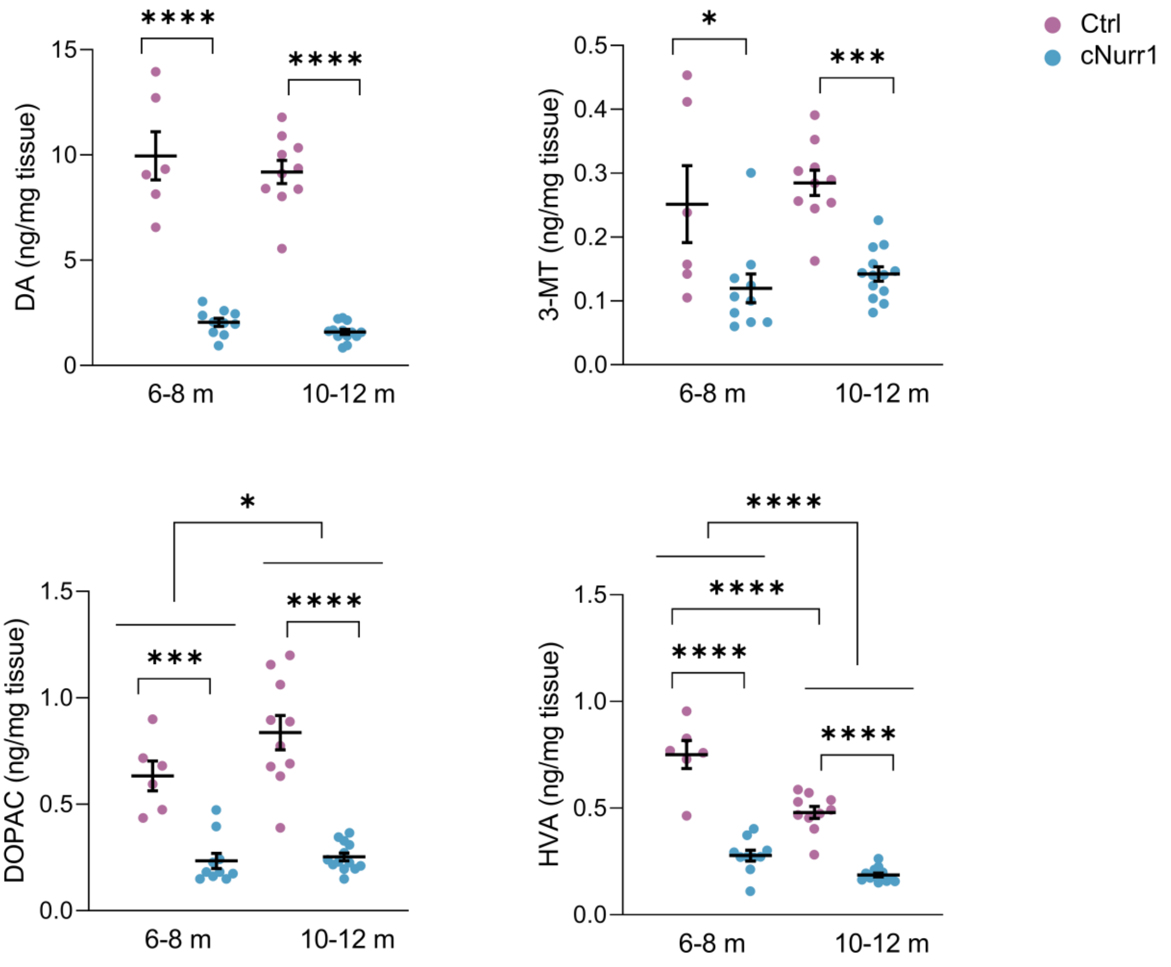

Supplemental Figure 7: The amounts of DA and 3-MT, but not DOPAC and HVA, in the striatum of middle-aged cNurr1 mice are not further reduced compared to adult mice. Amounts of DA and its metabolites 3-MT, DOPAC and HVA measured with HPLC in the striatum of $N = 10$ Ctrl and 13 cNurr1 mice aged 10-12 months. Data from the 6-8 months group are the same as those shown in Fig. 1 and are included in the graphs for comparison. $^{*}P < 0.05$; $^{***}P < 0.001$; $^{****}P < 0.0001$; Two-way ANOVA followed by multiple comparisons (Tukey).
