# Supplementary material for: Aberrant somatic calcium channel function in cNurr1 and LRRK2-G2019S mice
Source: NPJ Parkinsons Dis. 2023 Apr 7;9:56. doi: 10.1038/s41531-023-00500-5 (PMC10082048; doi:10.1038/s41531-023-00500-5)

LRRK2 Striatum Gels 1-2

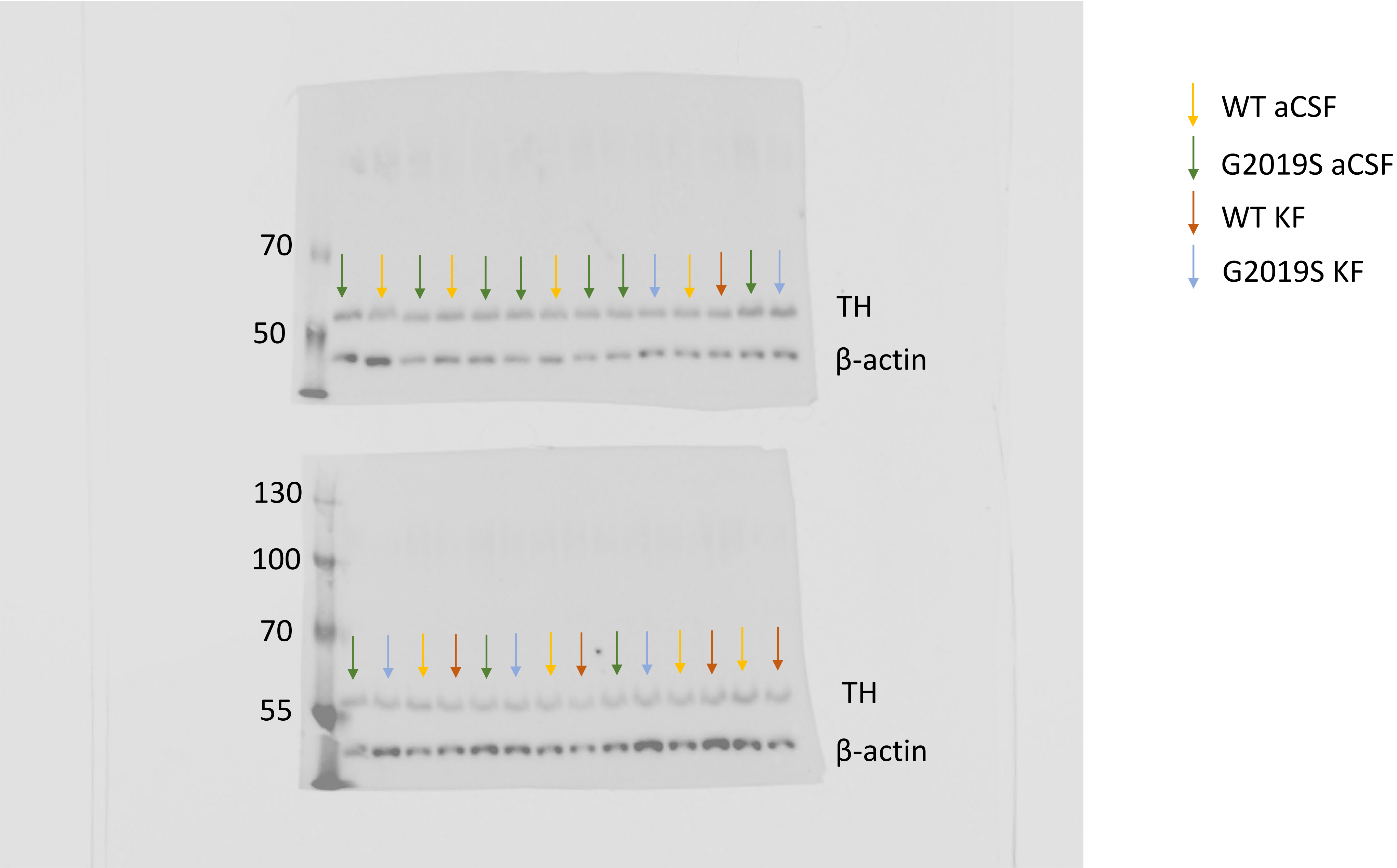

LRRK2 Striatum Gels 1-2

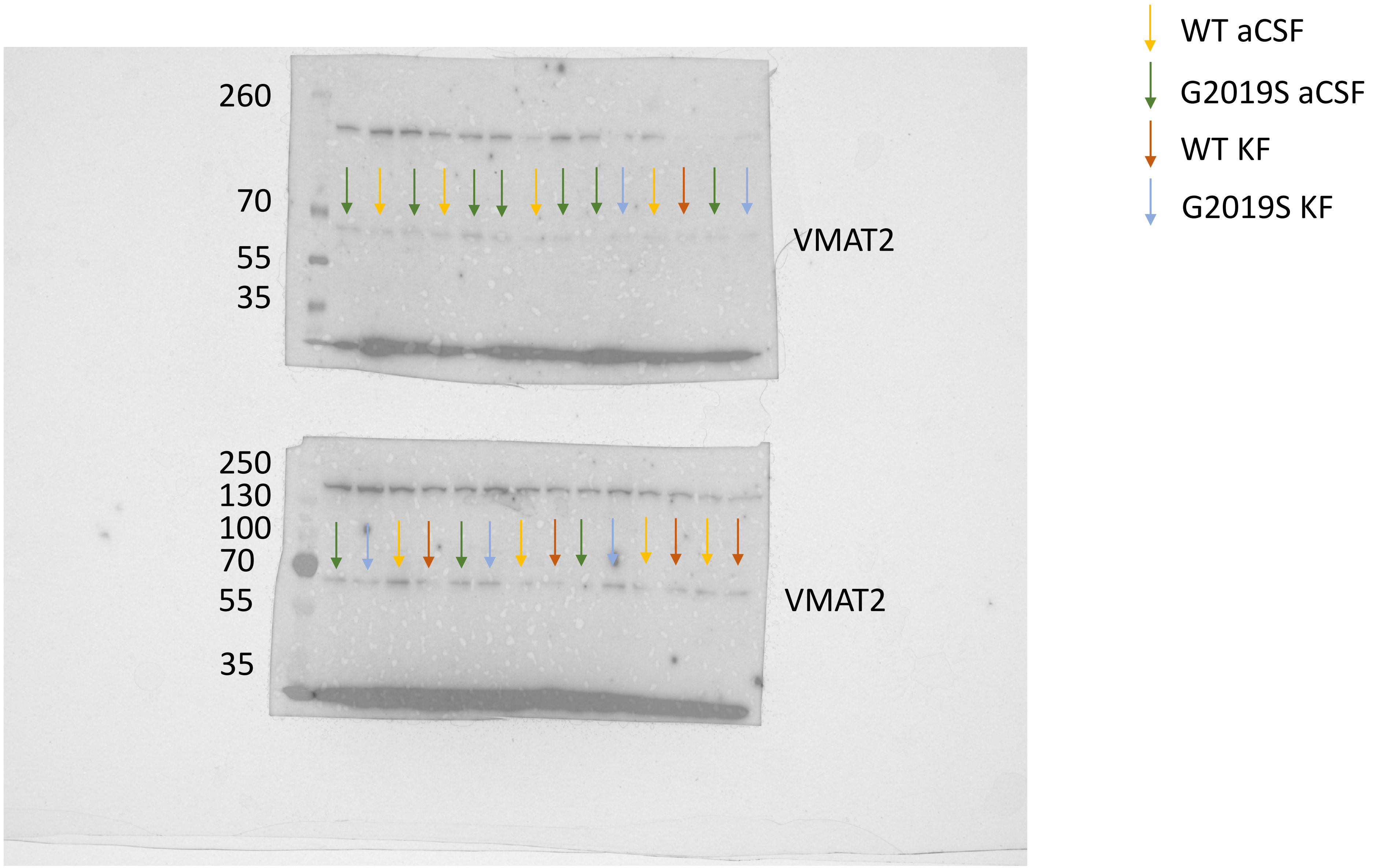

LRRK2 Striatum Gels 1-2

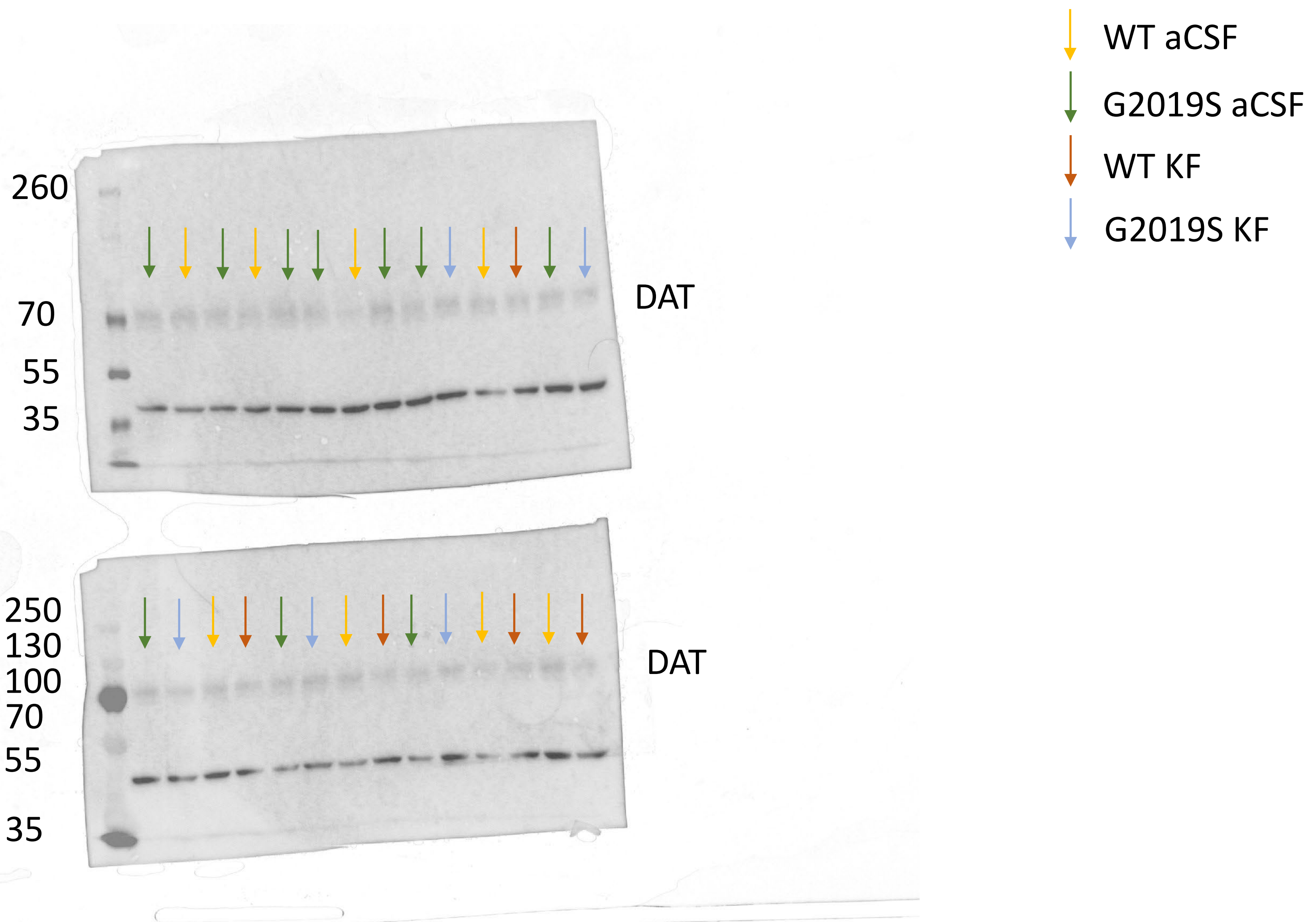

LRRK2 Striatum Gels 3-4

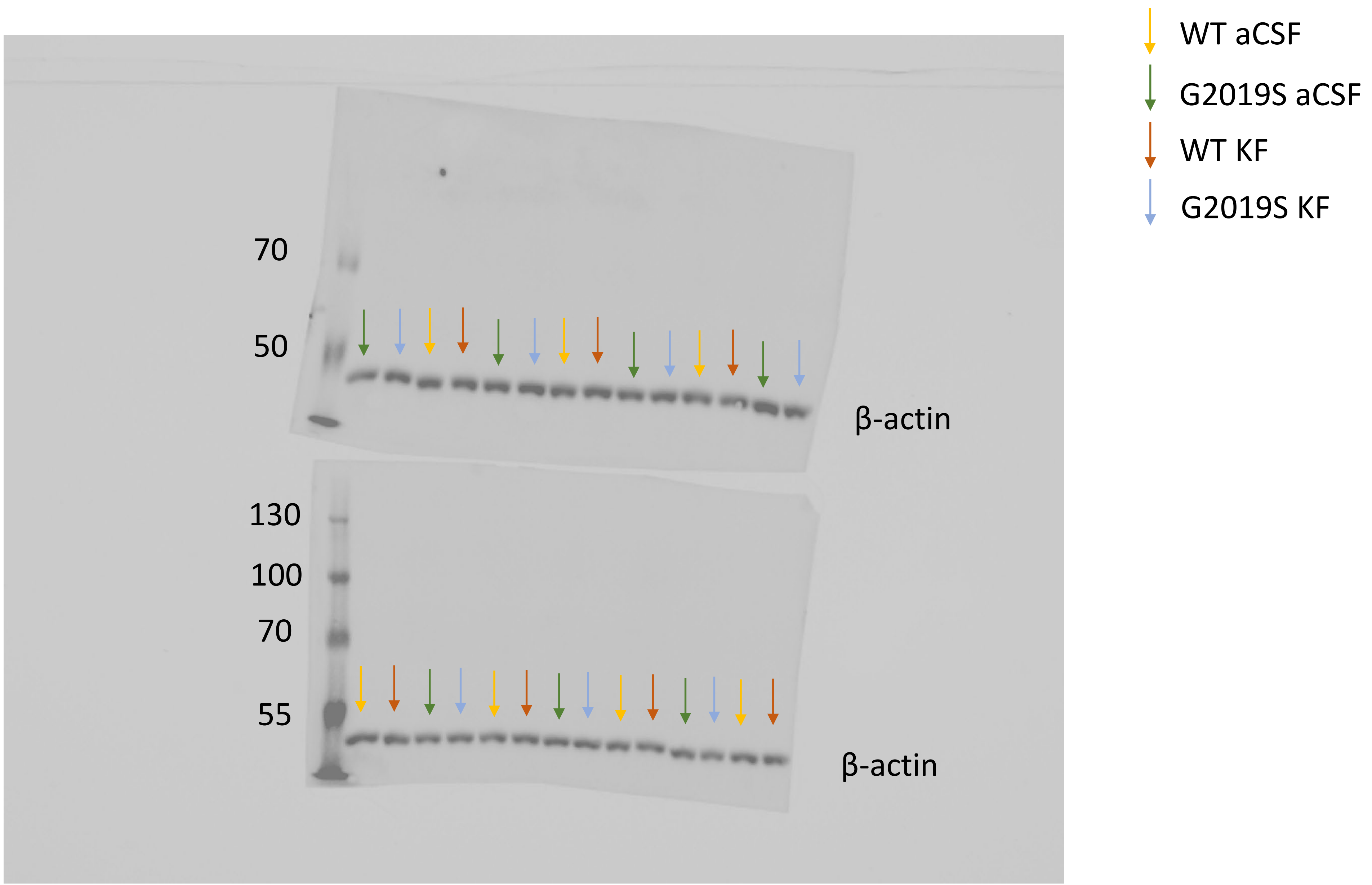

LRRK2 Striatum Gels 3-4

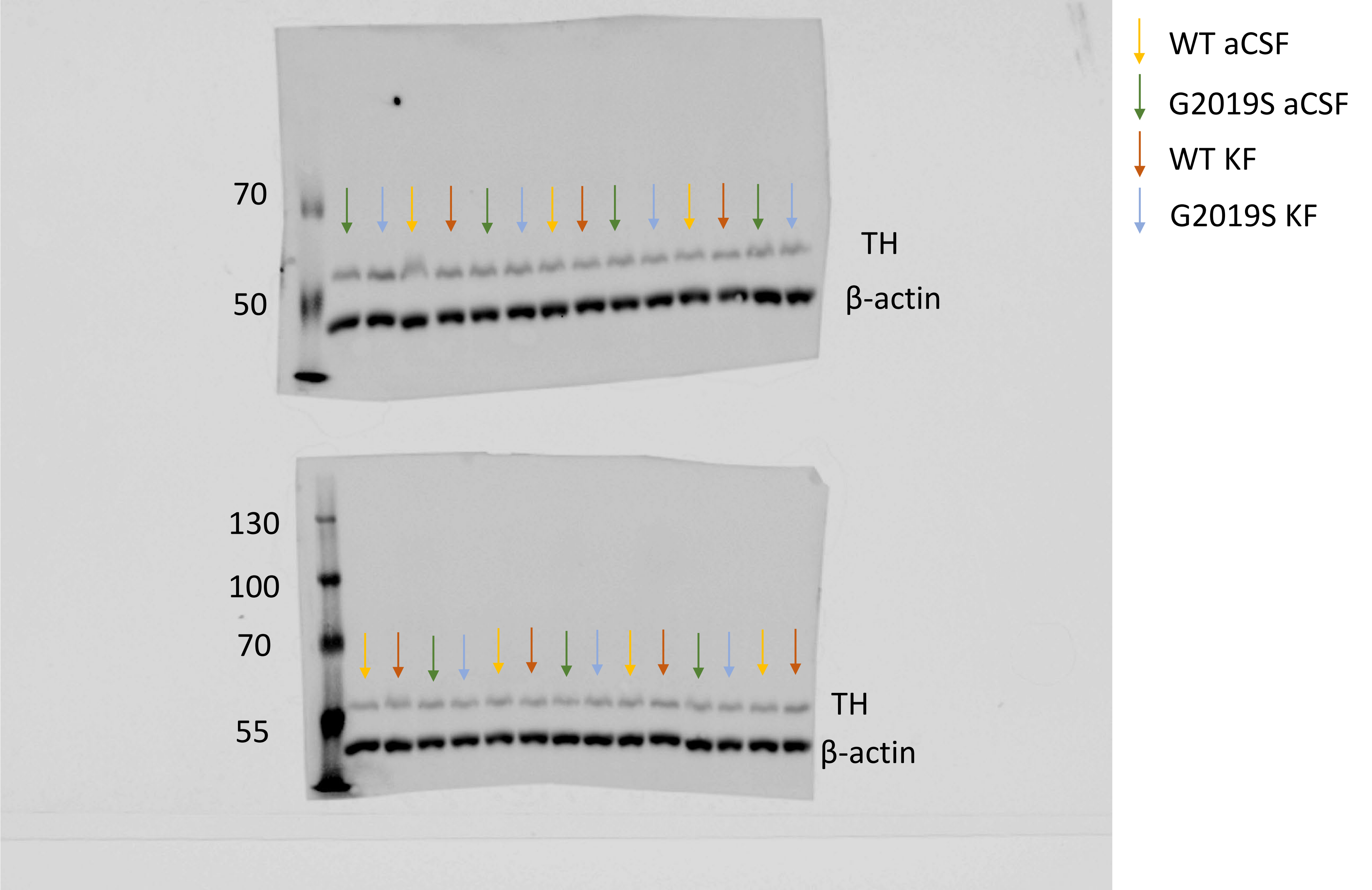

LRRK2 Striatum Gels 3-4

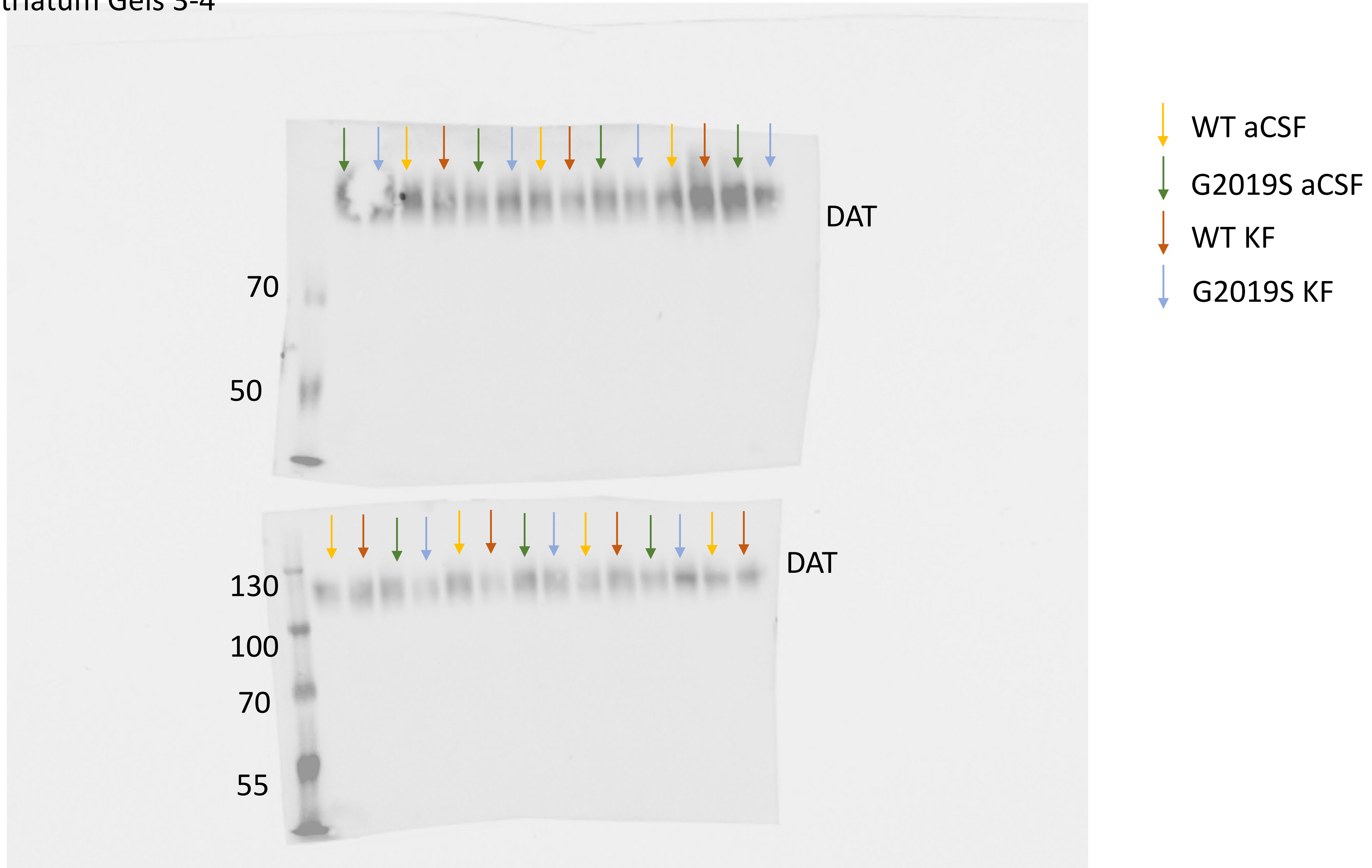

LRRK2 Striatum Gels 3-4

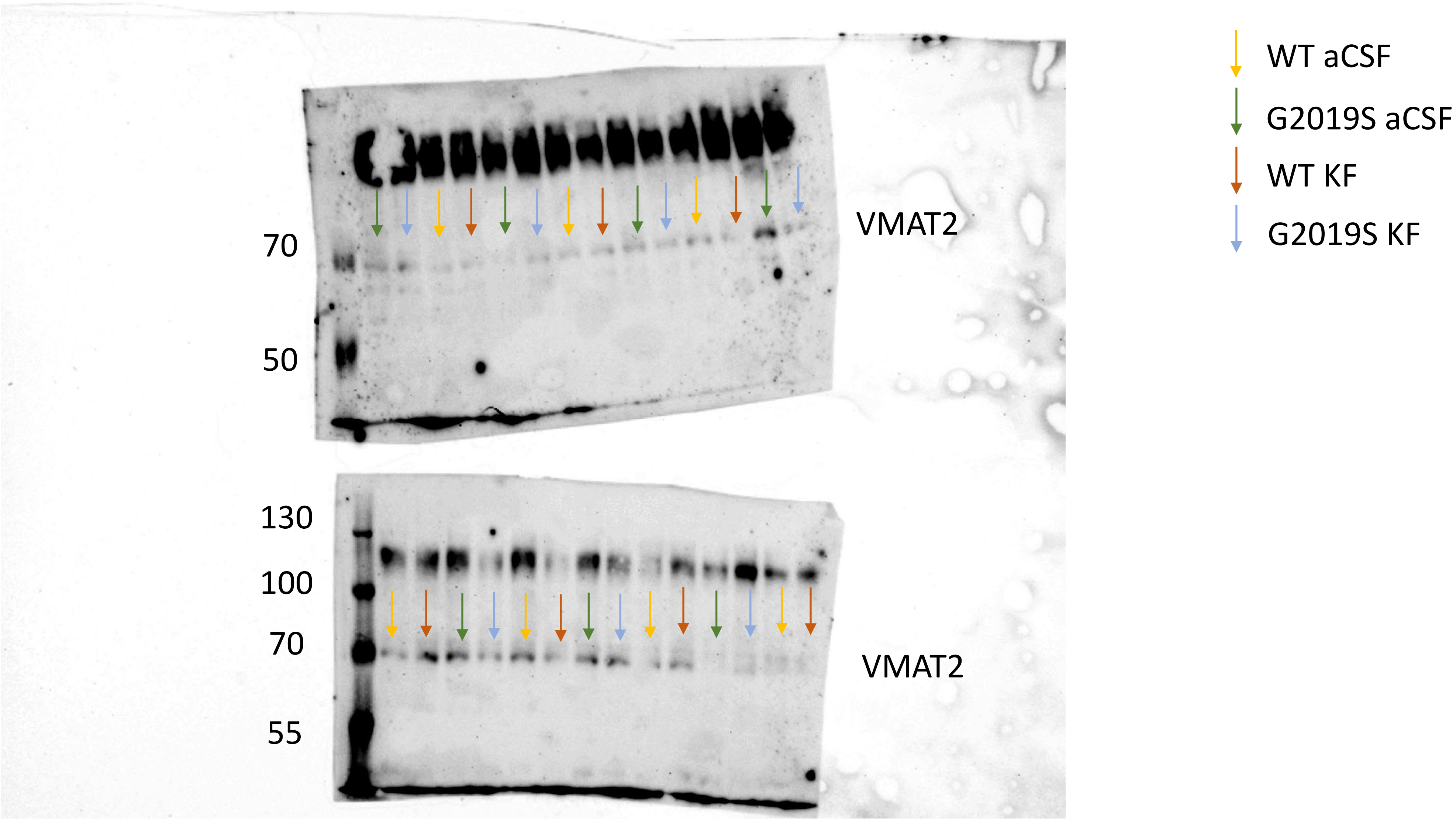

LRRK2 Striatum Gels 5-6

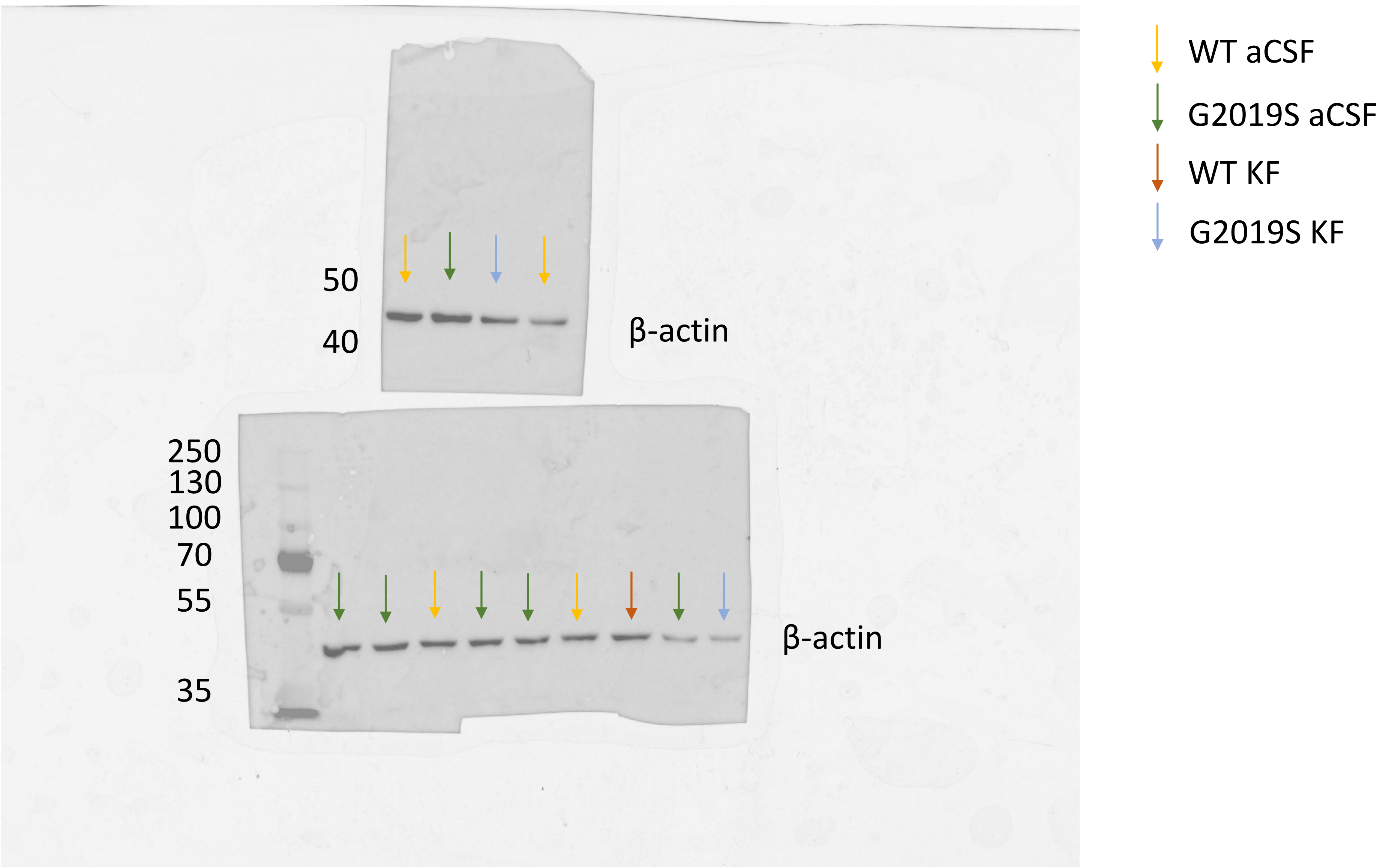

LRRK2 Striatum Gels 5-6

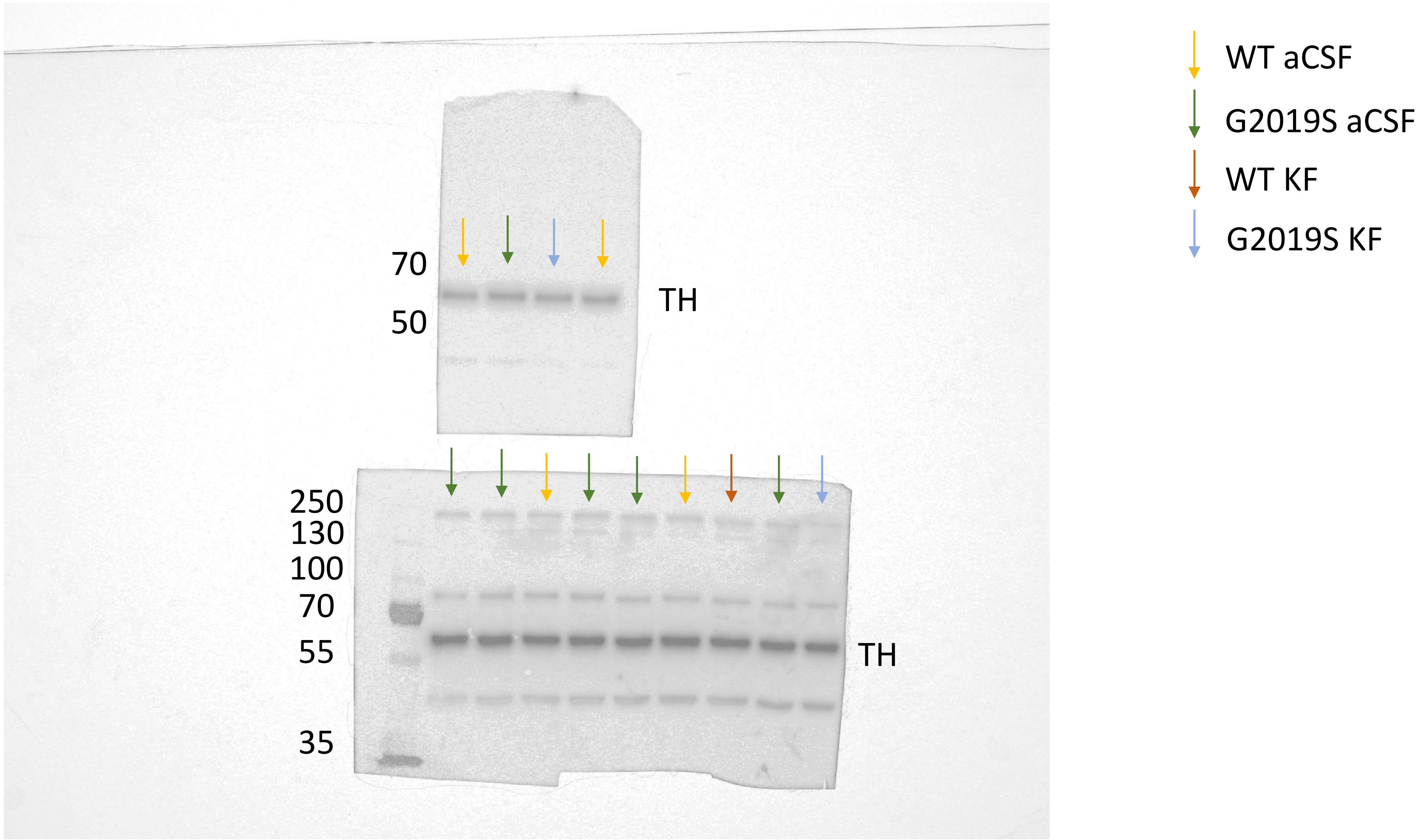

LRRK2 Striatum Gels 5-6

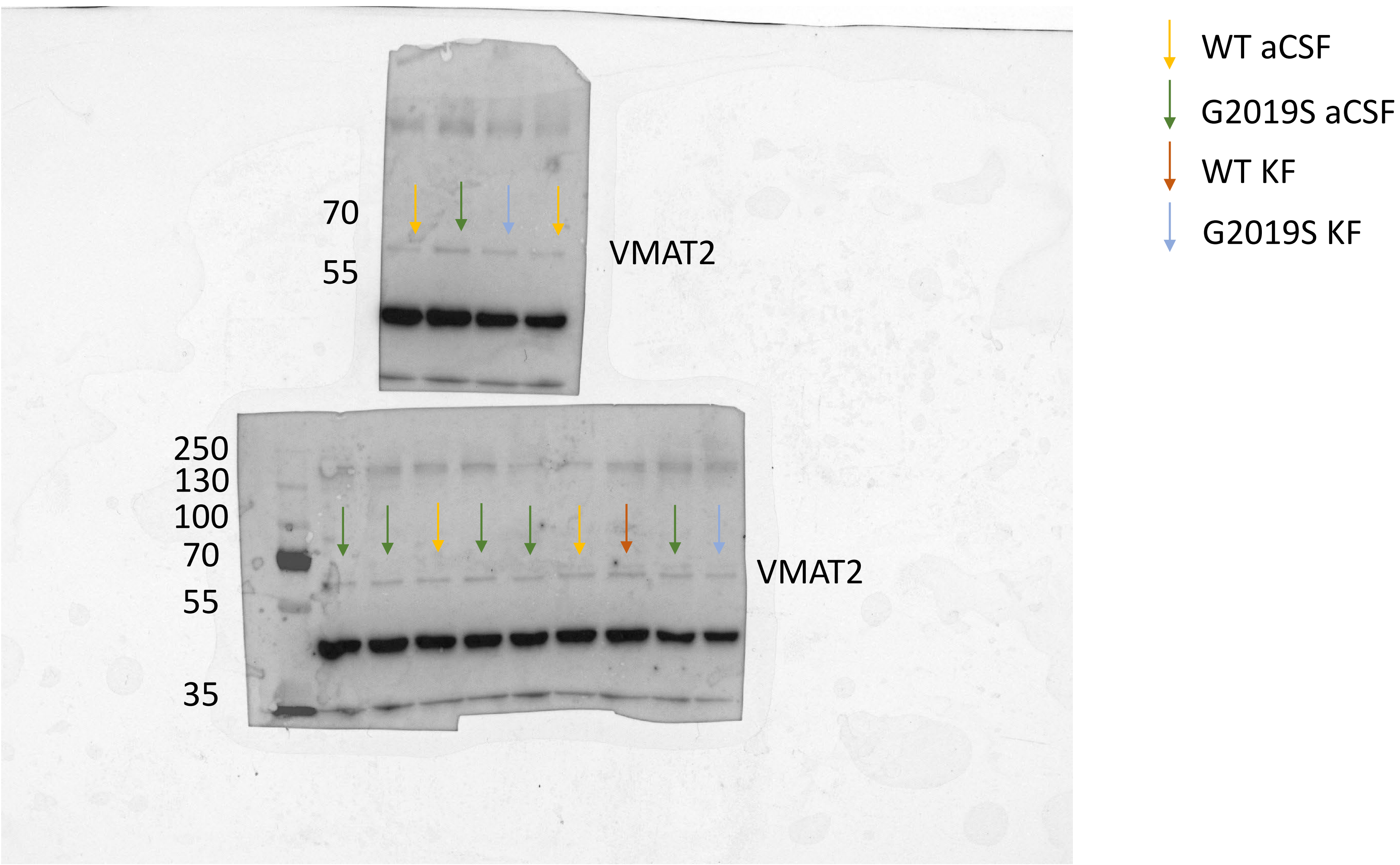

LRRK2 Striatum Gels 5-6

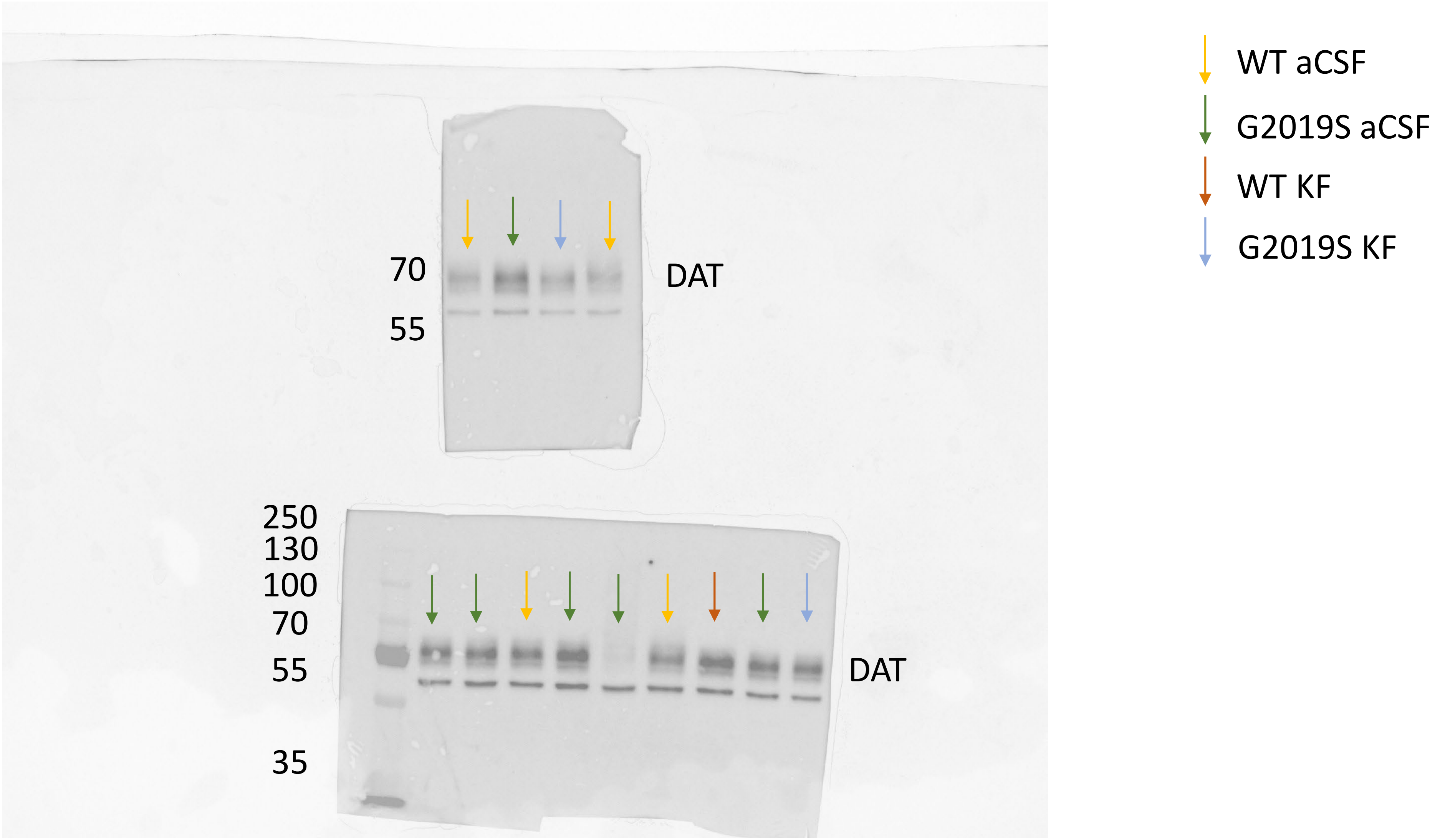

LRRK2 Substantia Nigra Gels 1-2

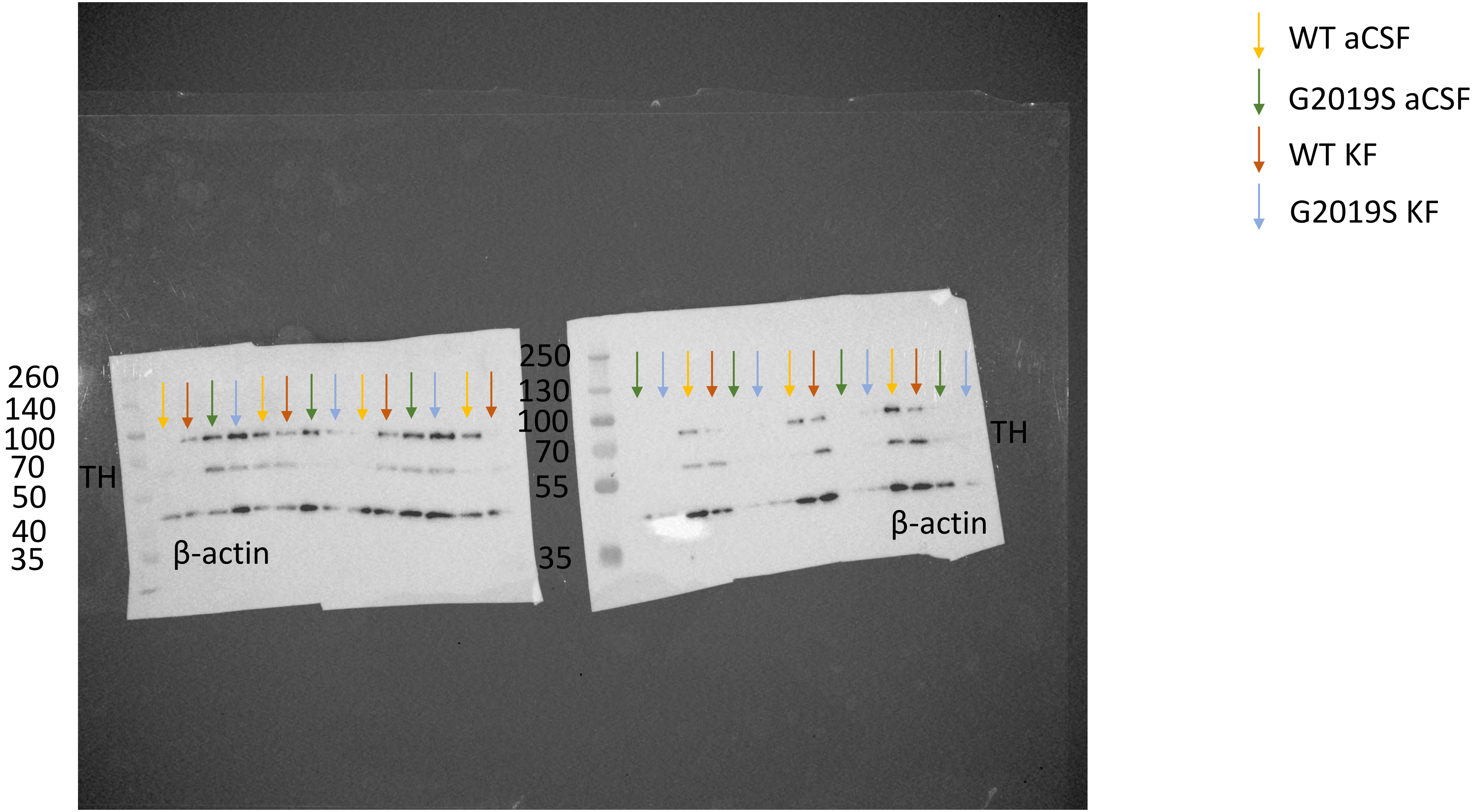

LRRK2 Substantia Nigra Gels 1-2

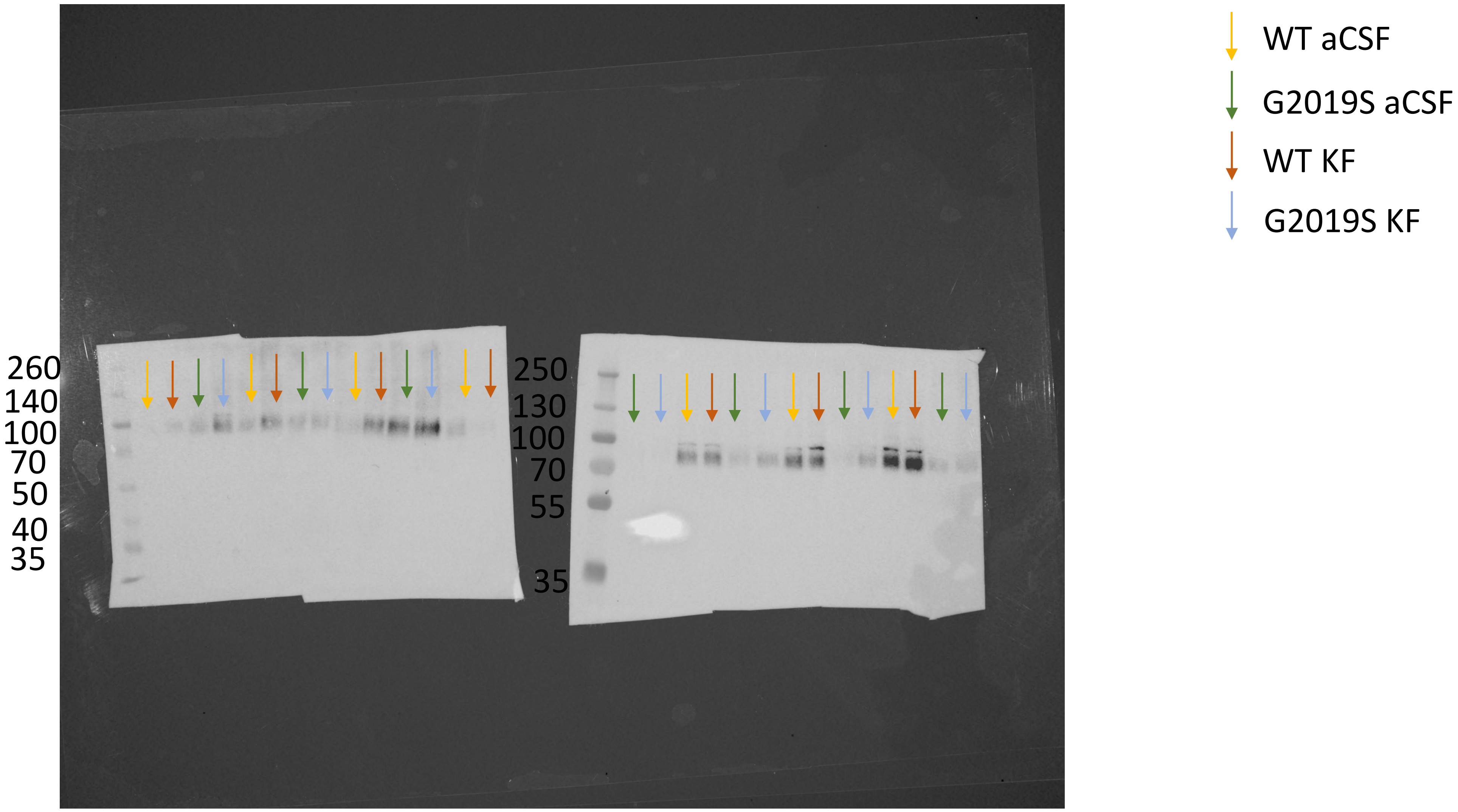

LRRK2 Substantia Nigra Gels 3-4

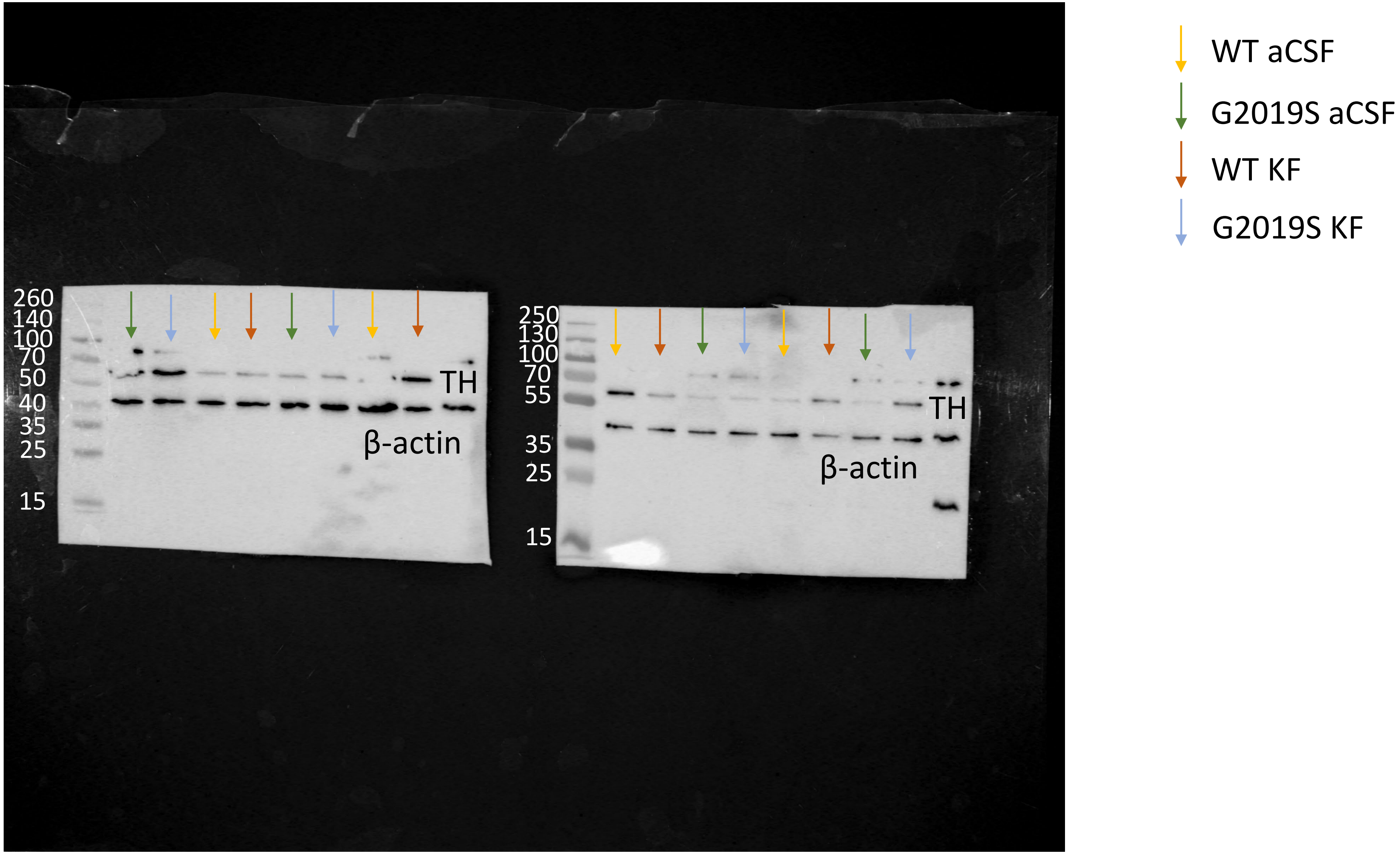

LRRK2 Substantia Nigra Gels 3-4

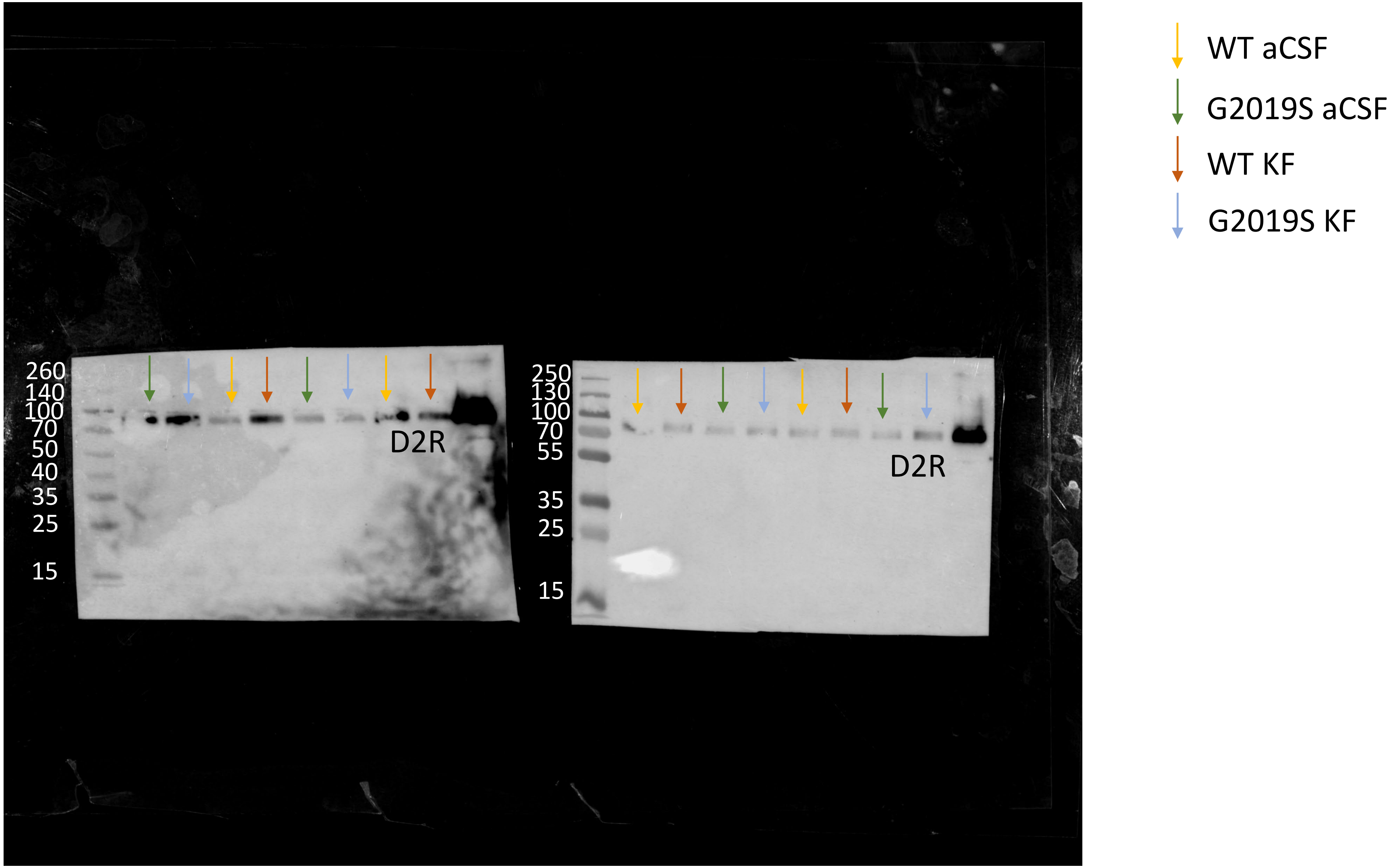

LRRK2 Substantia Nigra Gels 3-4

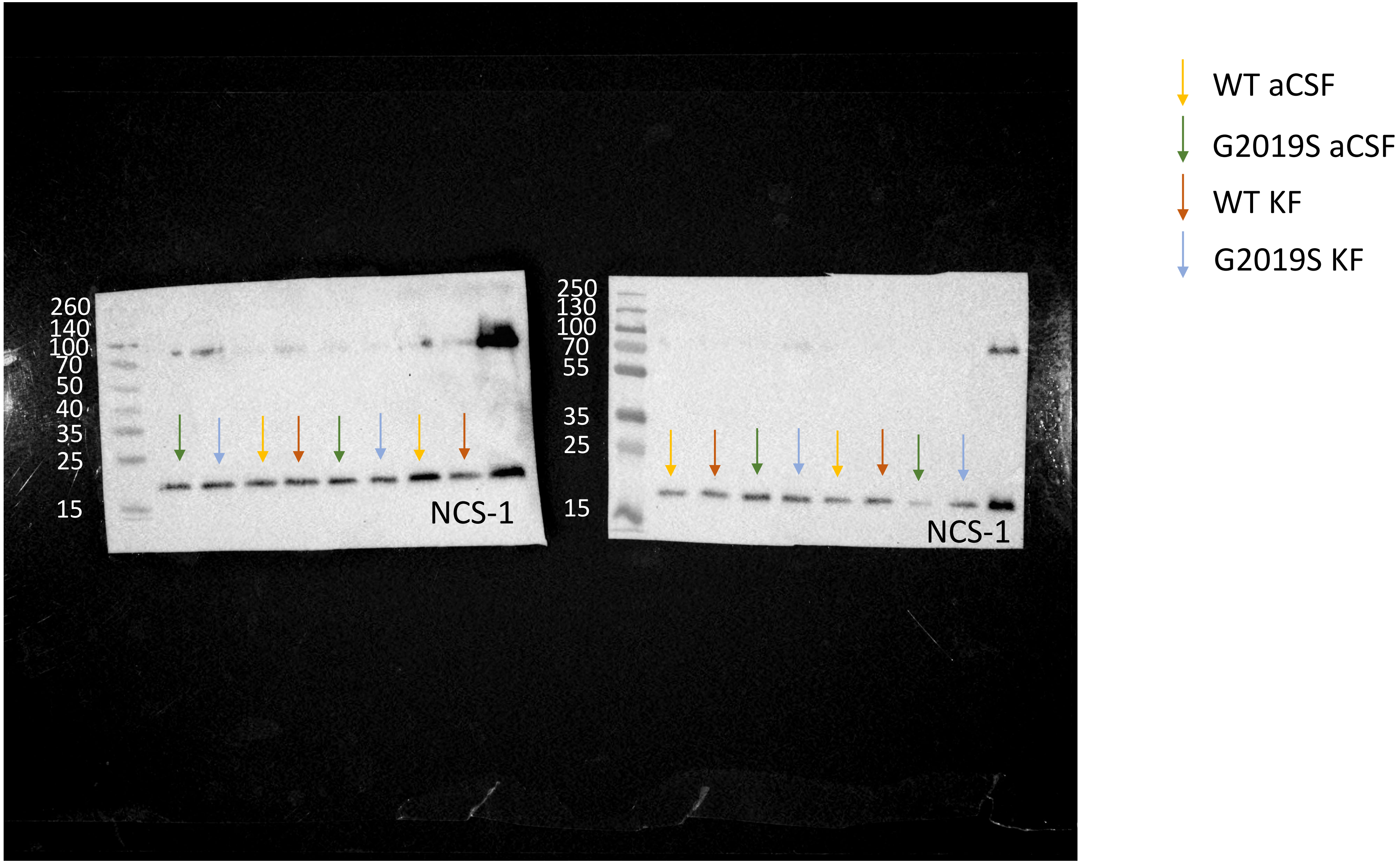

LRRK2 Substantia Nigra Gel 5

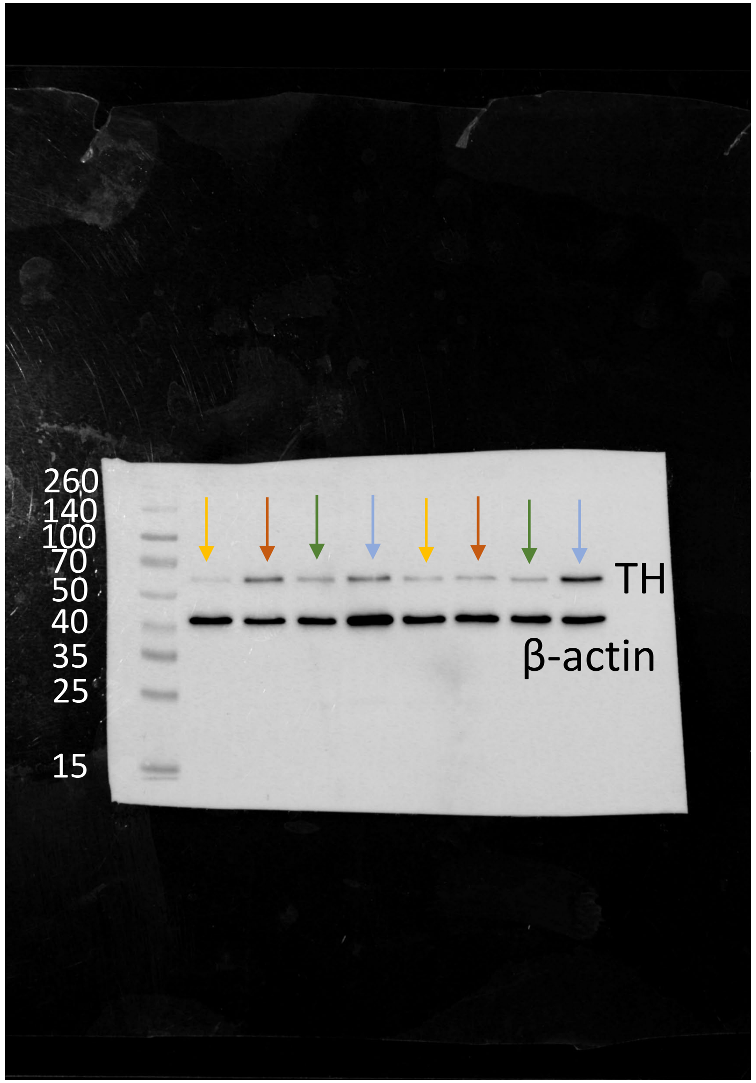

WT aCSF

G2019S aCSF

WT KF

G2019S KF

LRRK2 Substantia Nigra Gel 5

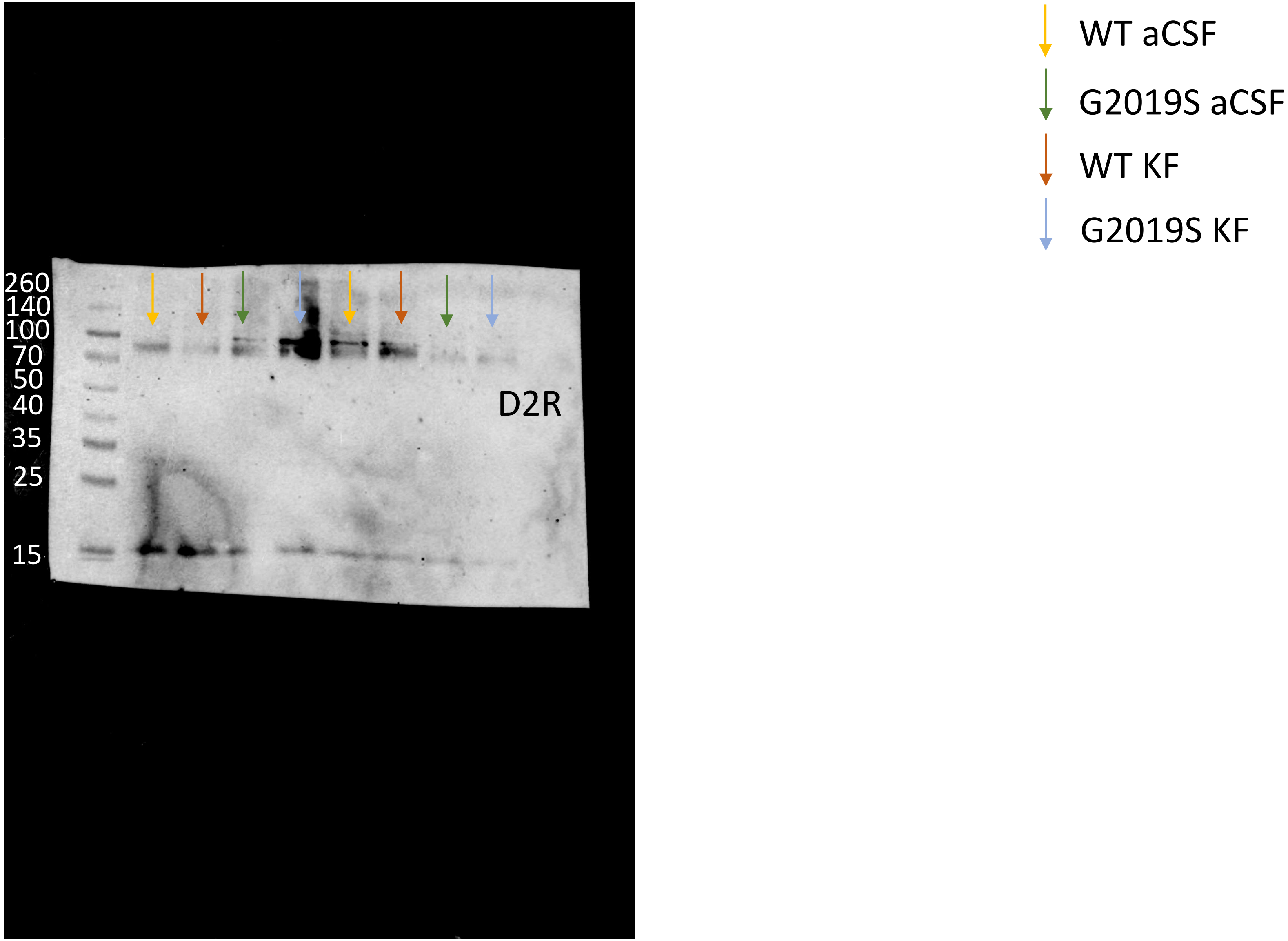

LRRK2 Substantia Nigra Gel 5

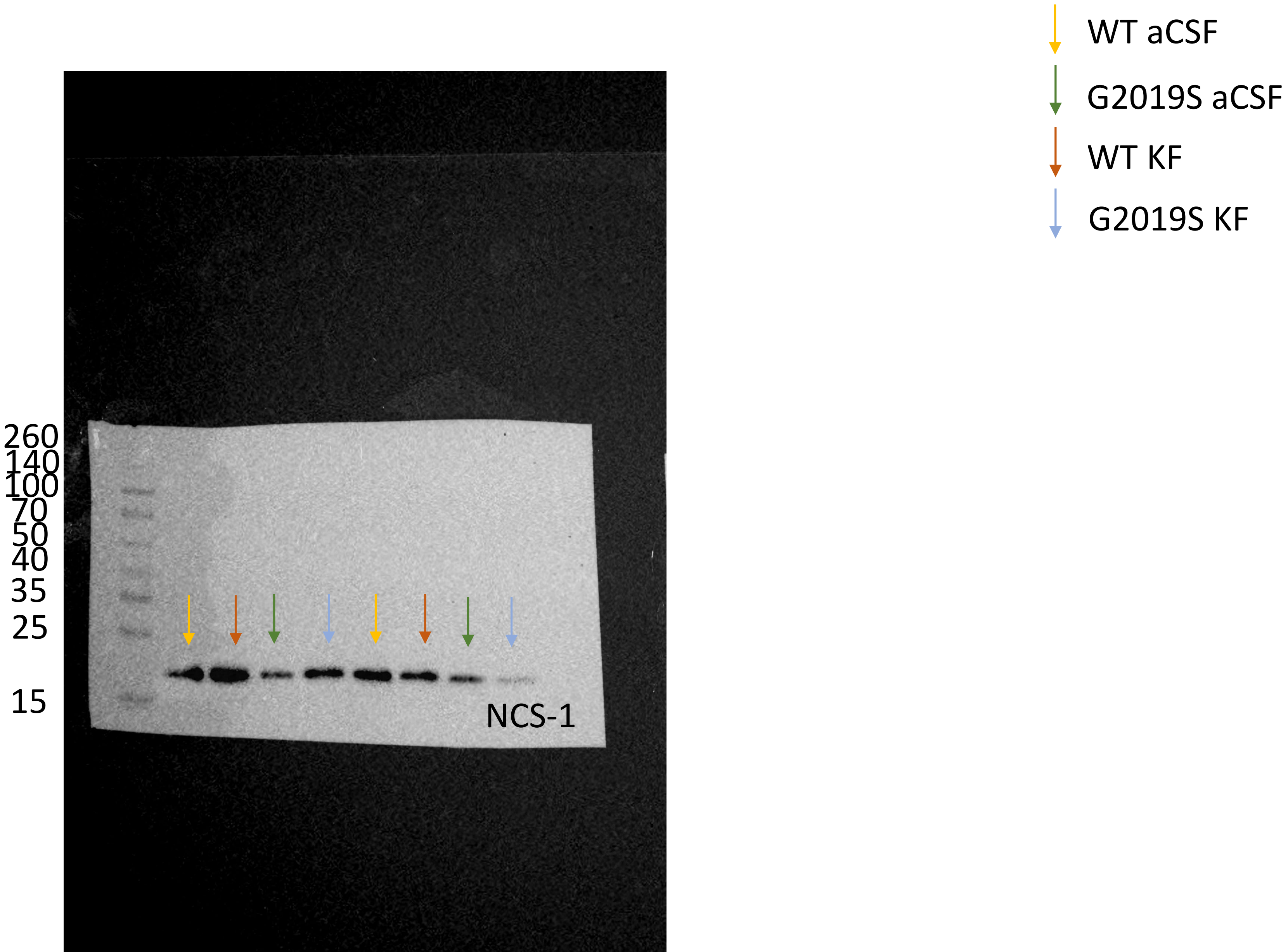

LRRK2 Substantia Nigra Gels 7-8

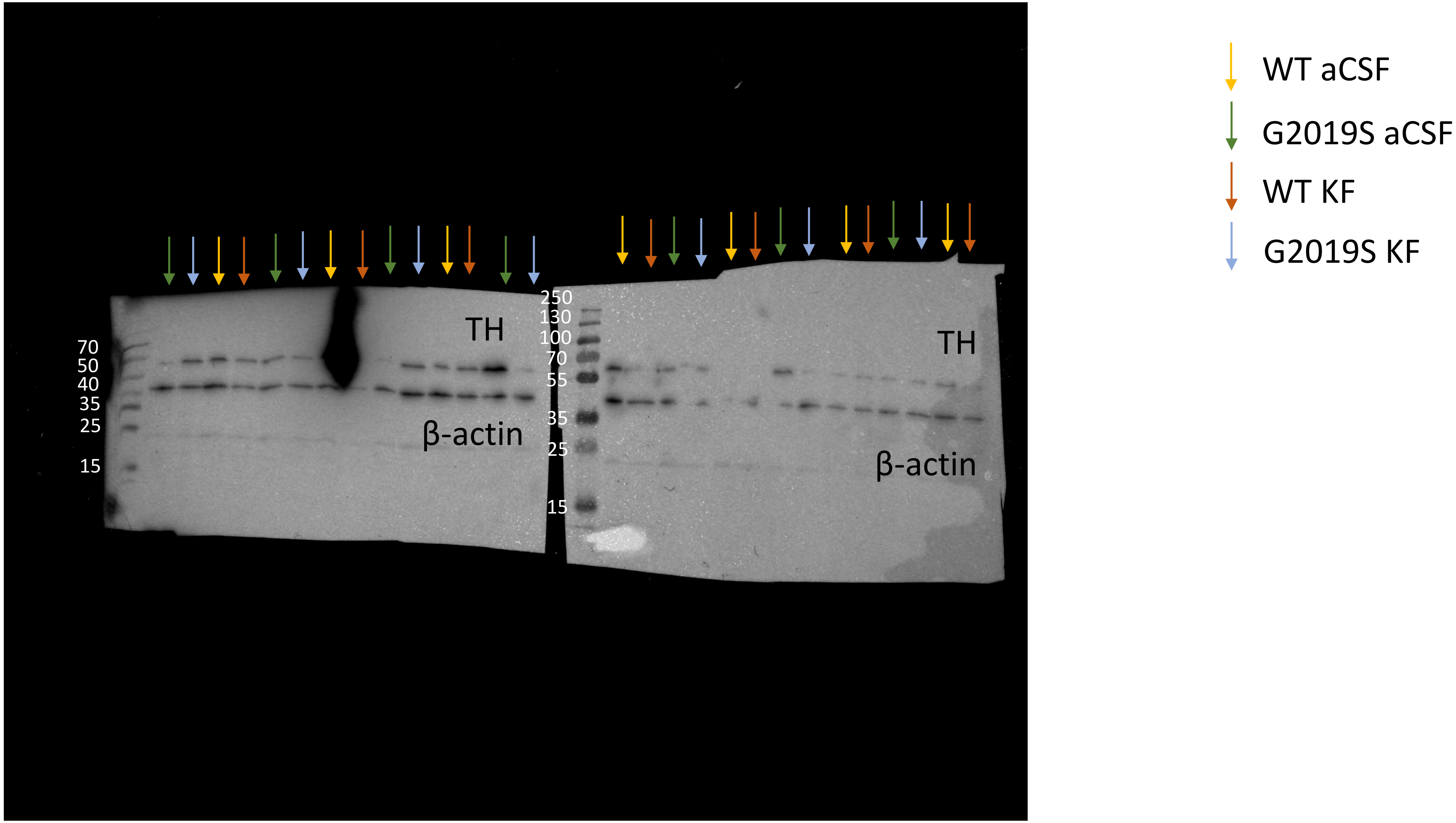

LRRK2 Substantia Nigra Gels 7-8

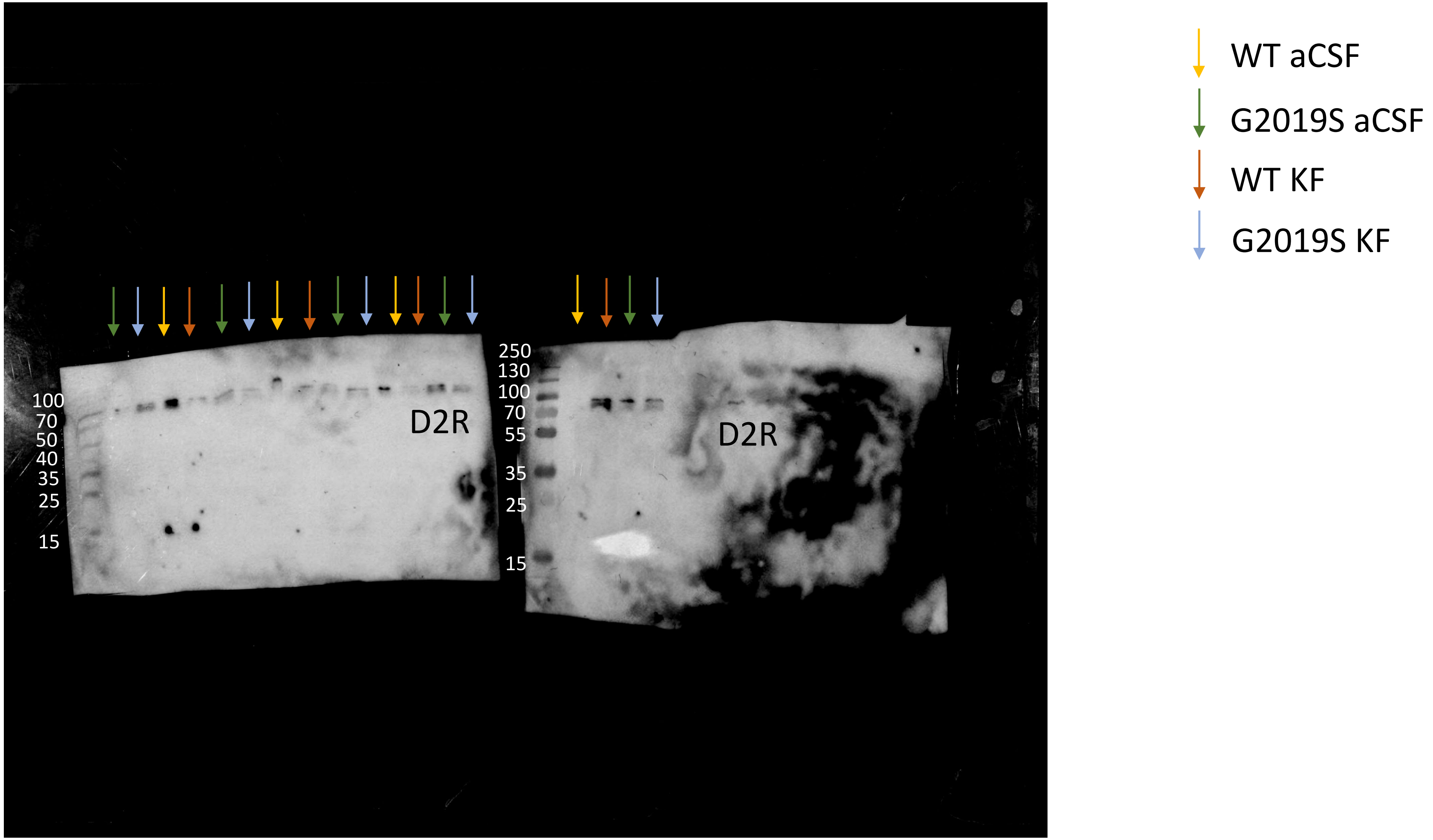

LRRK2 Substantia Nigra Gels 7-8

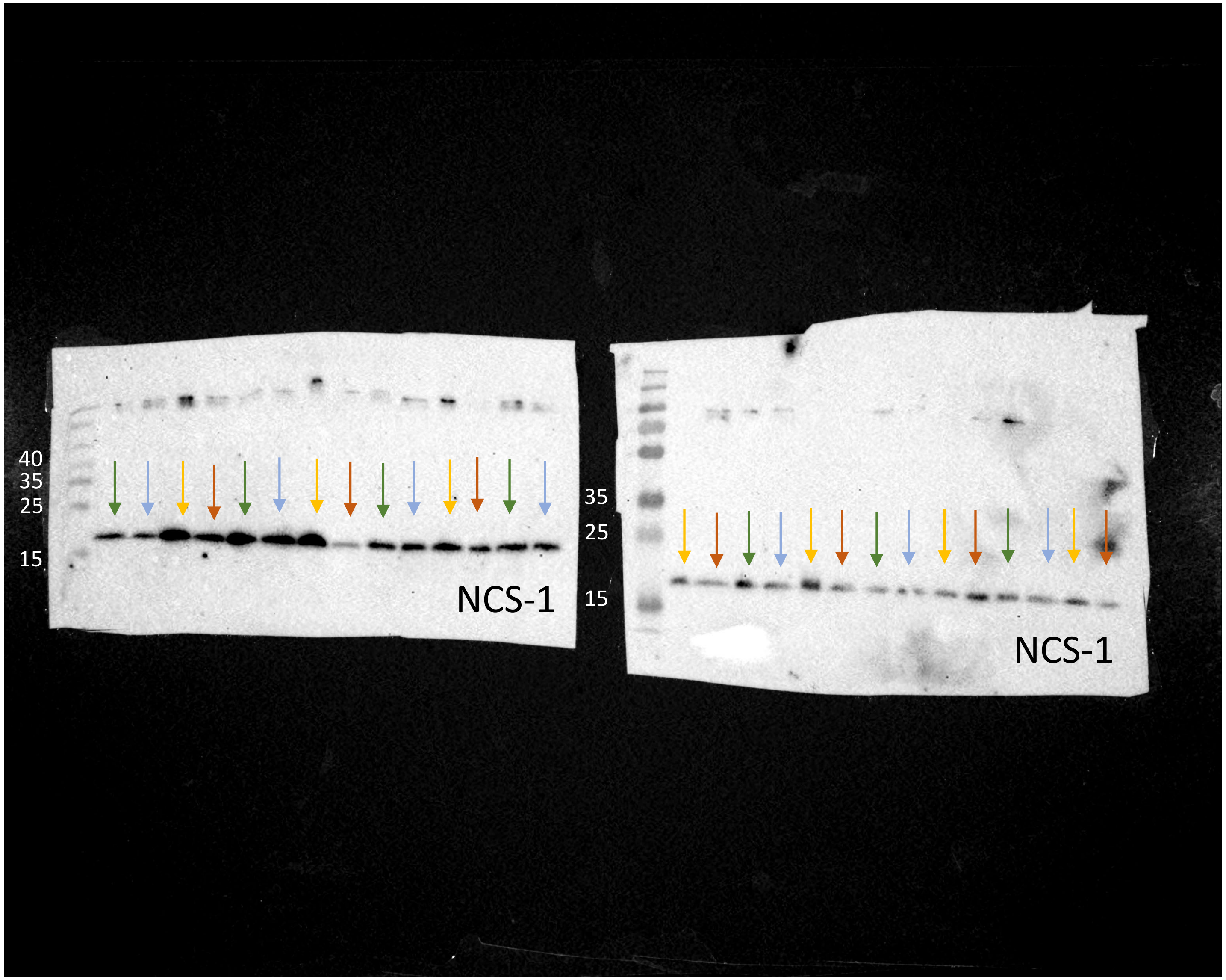

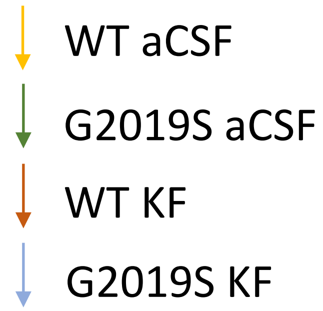

Nurr-1 Substantia Nigra Gels 1-2

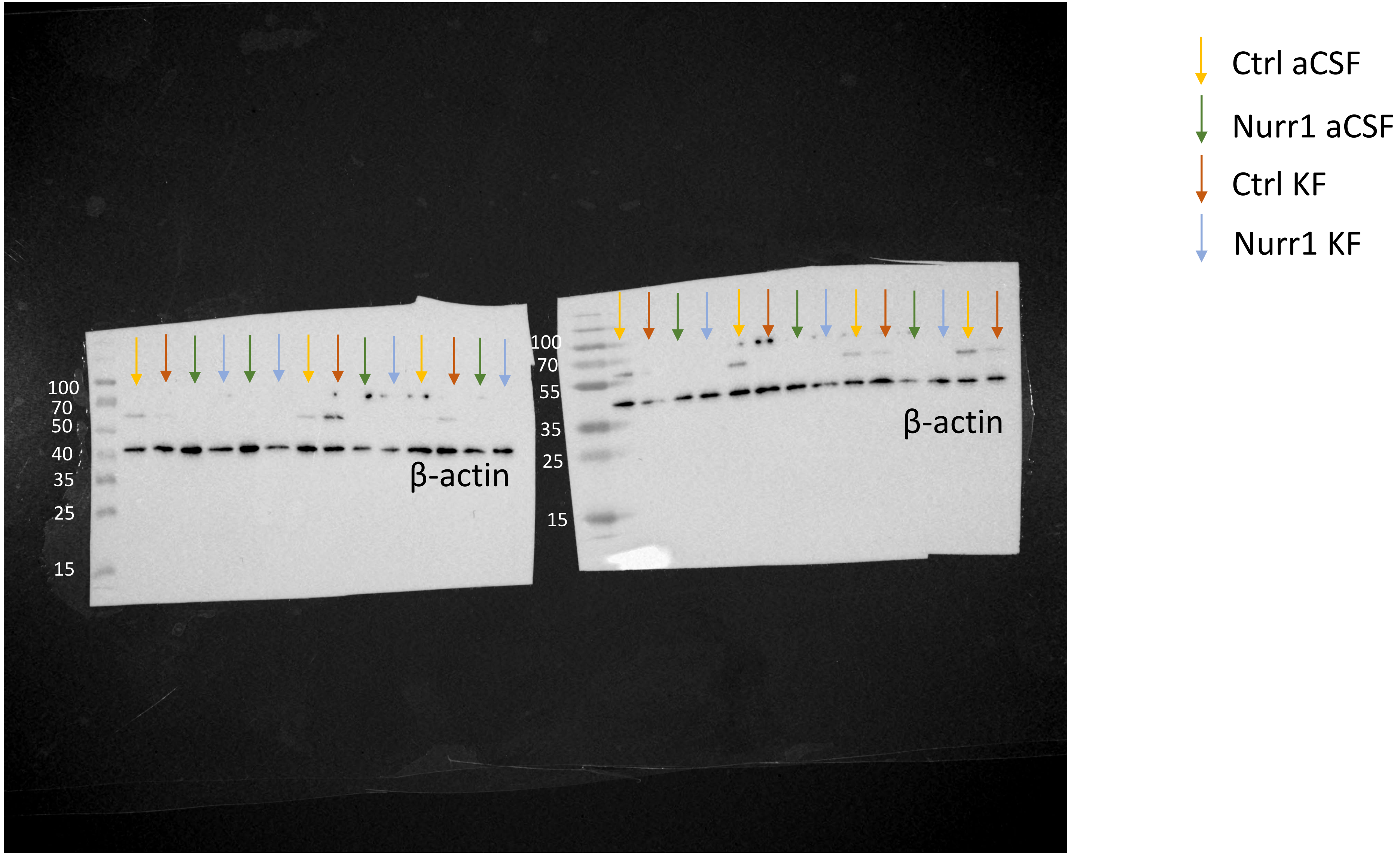

Nurr-1 Substantia Nigra Gels 1-2

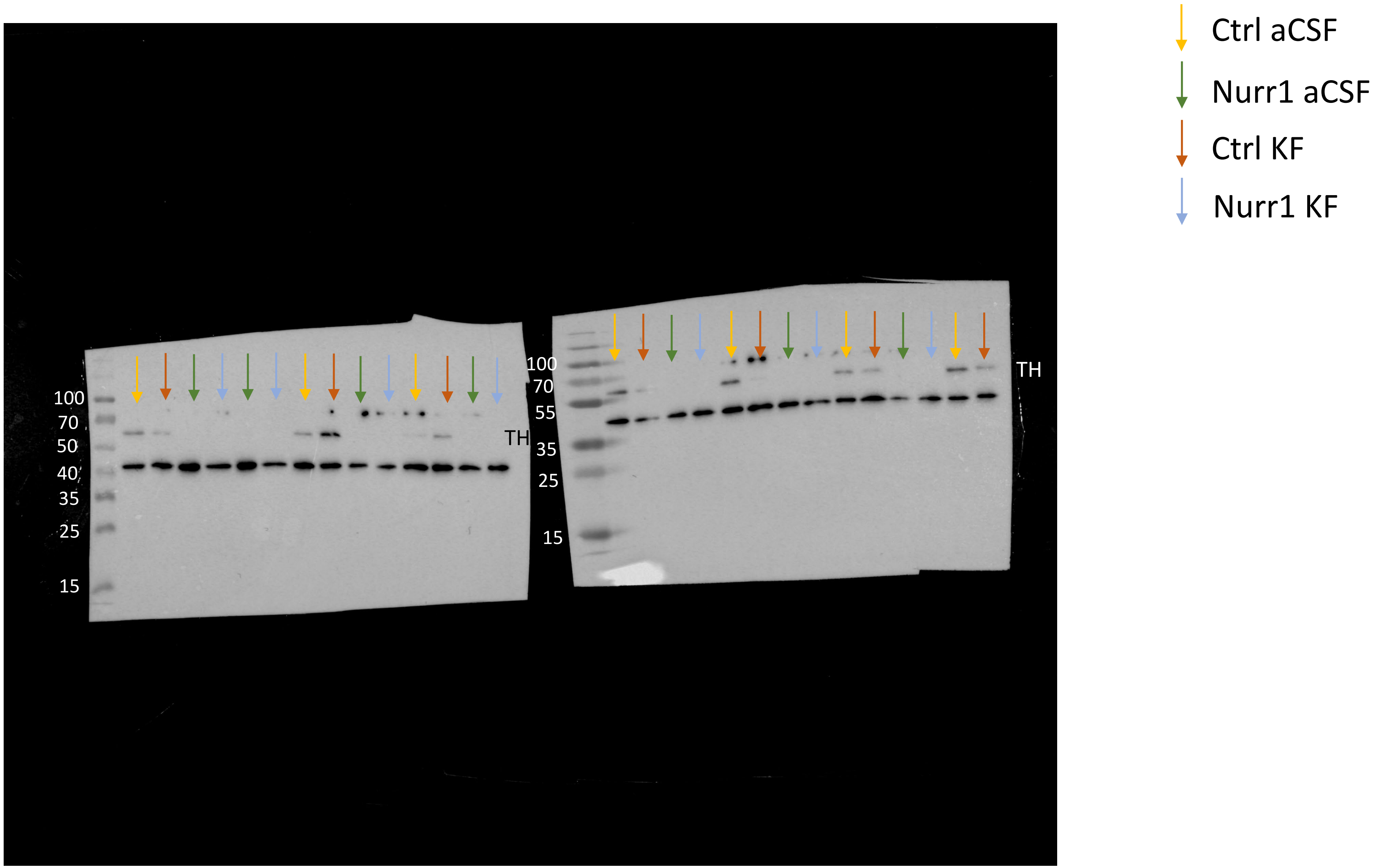

Nurr-1 Substantia Nigra Gels 1-2

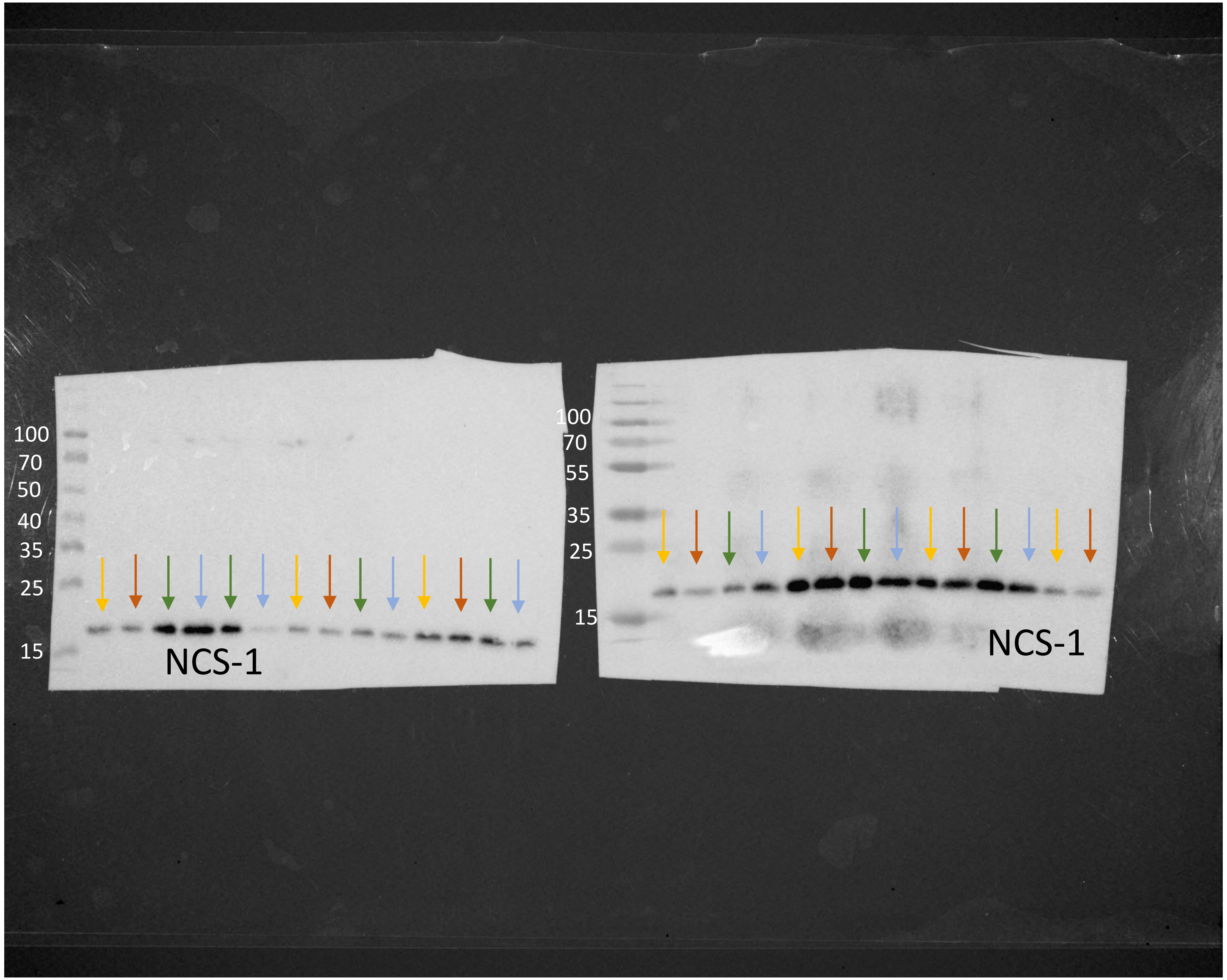

Ctrl aCSF

Nurr1 aCSF

Ctrl KF

Nurr1 KF

Nurr-1 Substantia Nigra Gels 1-2

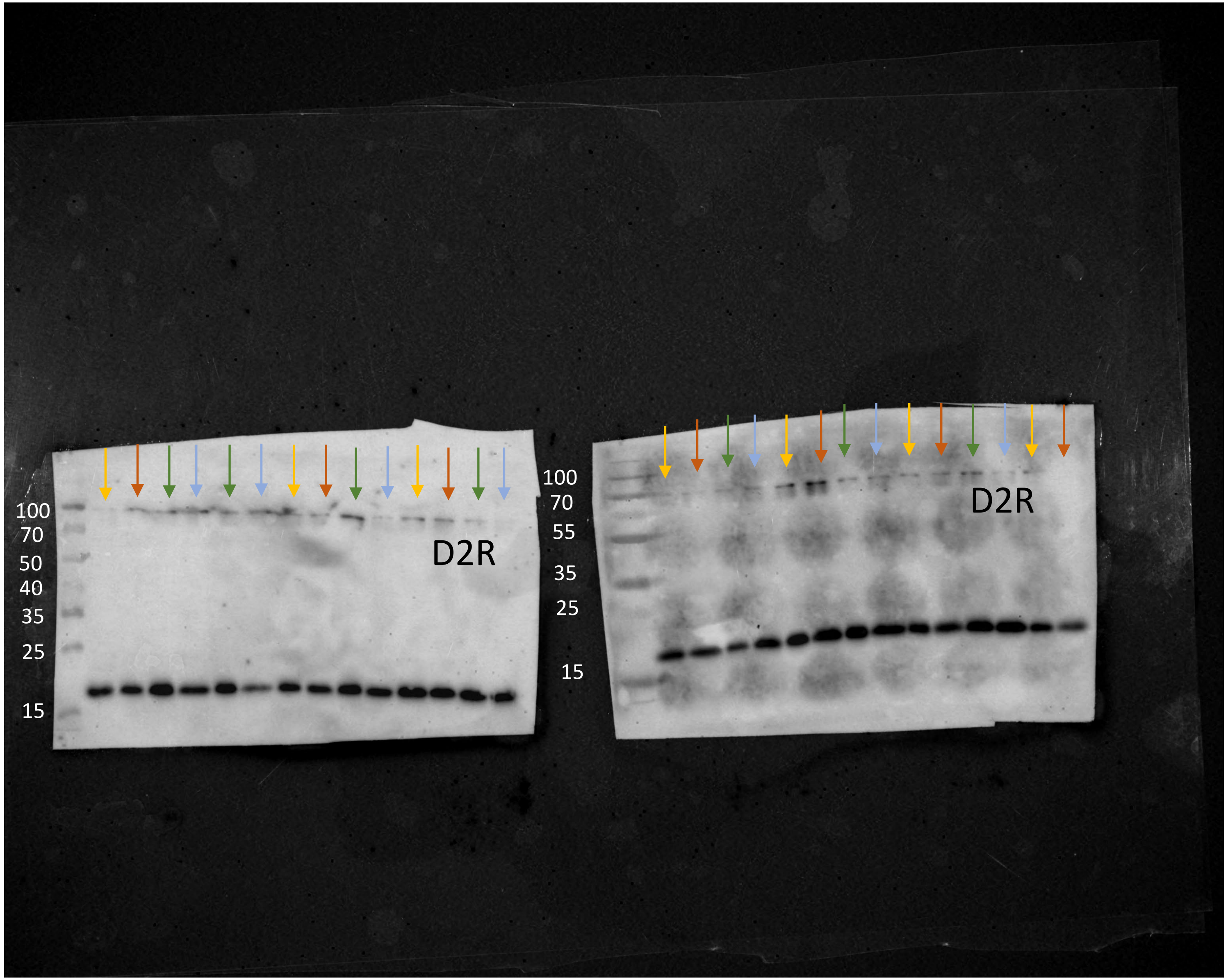

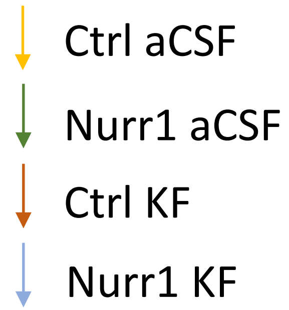

Nurr-1 Substantia Nigra Gels 3-4

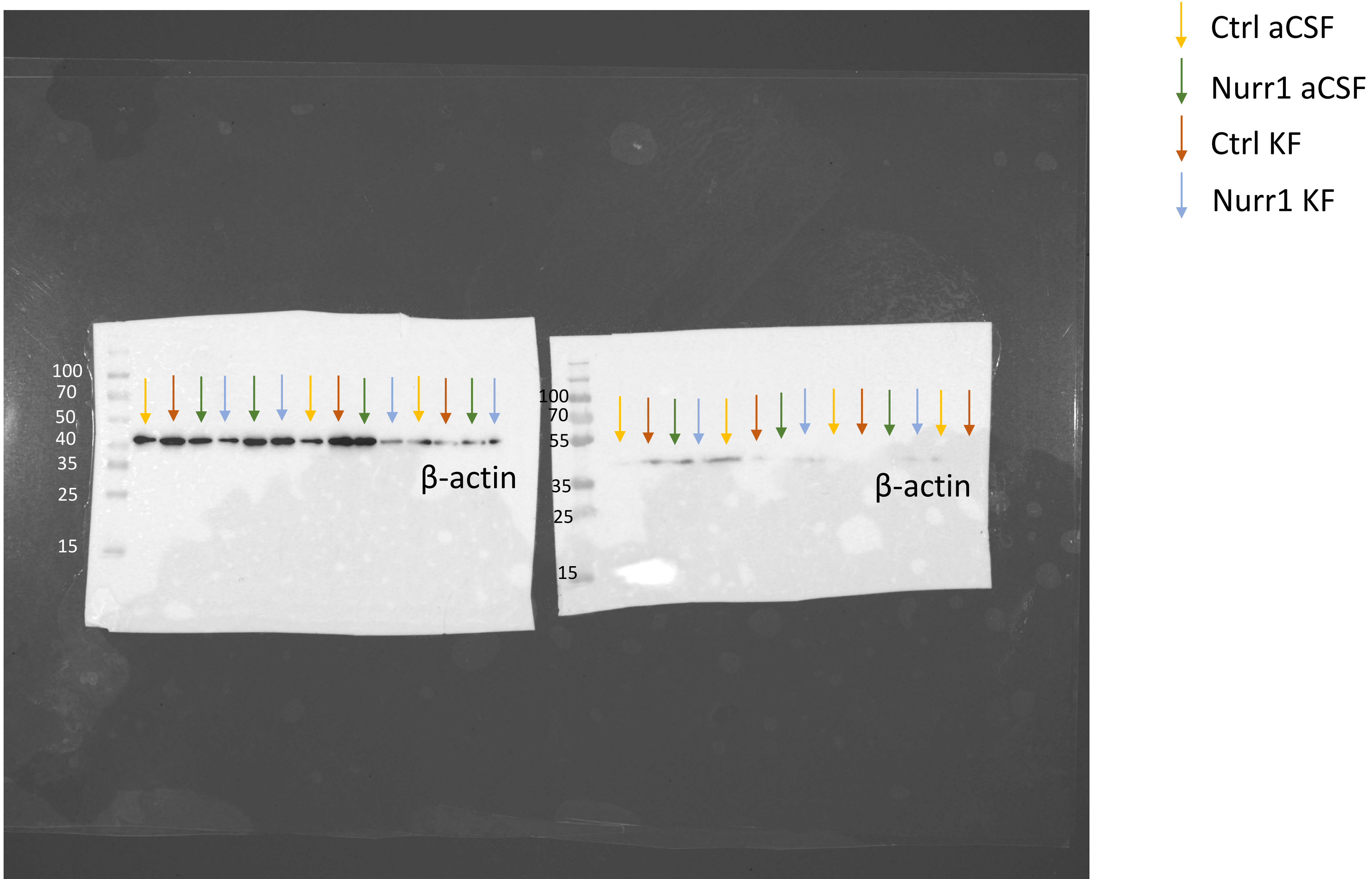

Nurr-1 Substantia Nigra Gels 3-4

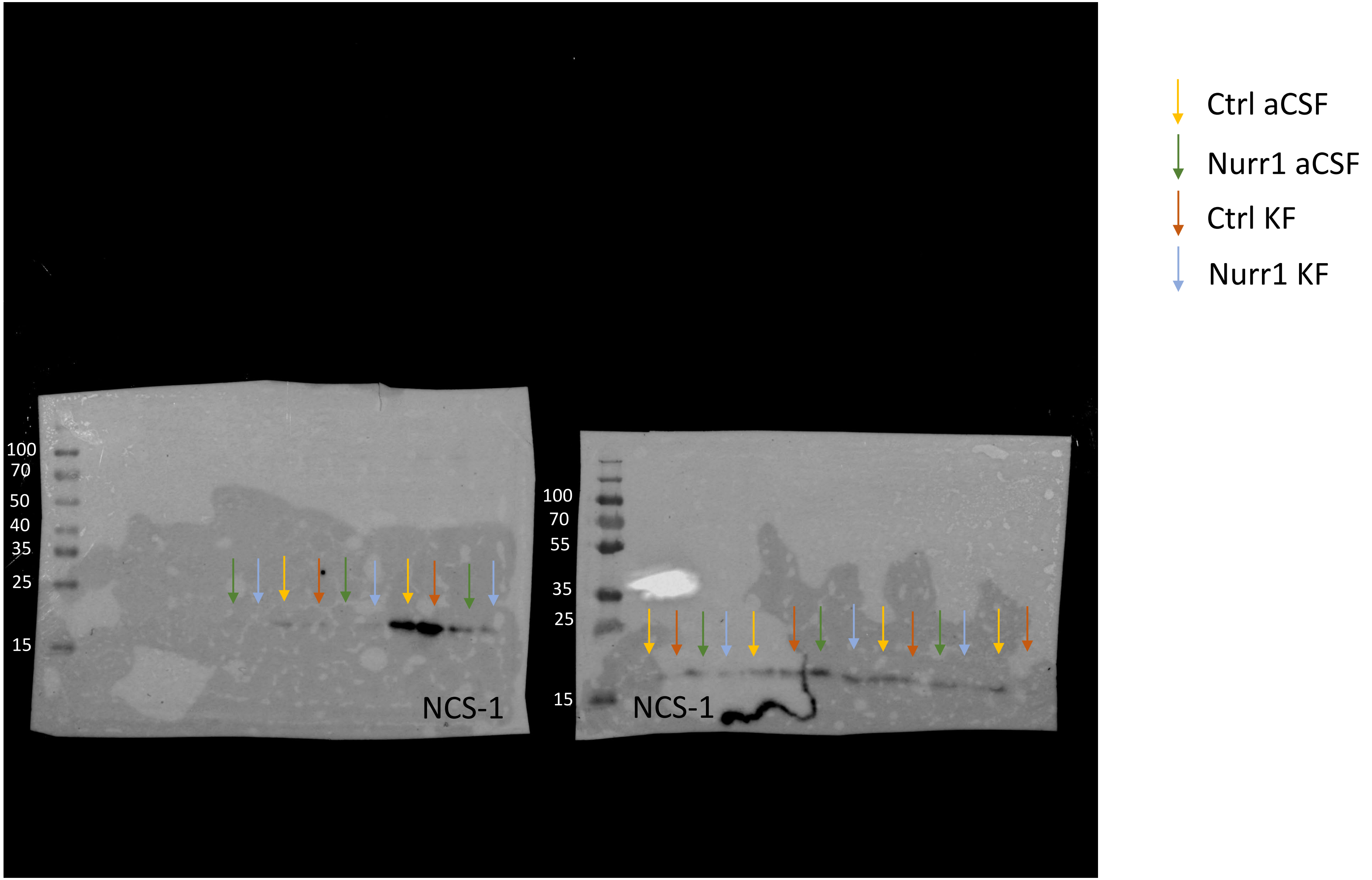

Nurr-1 Substantia Nigra Gels 3-4

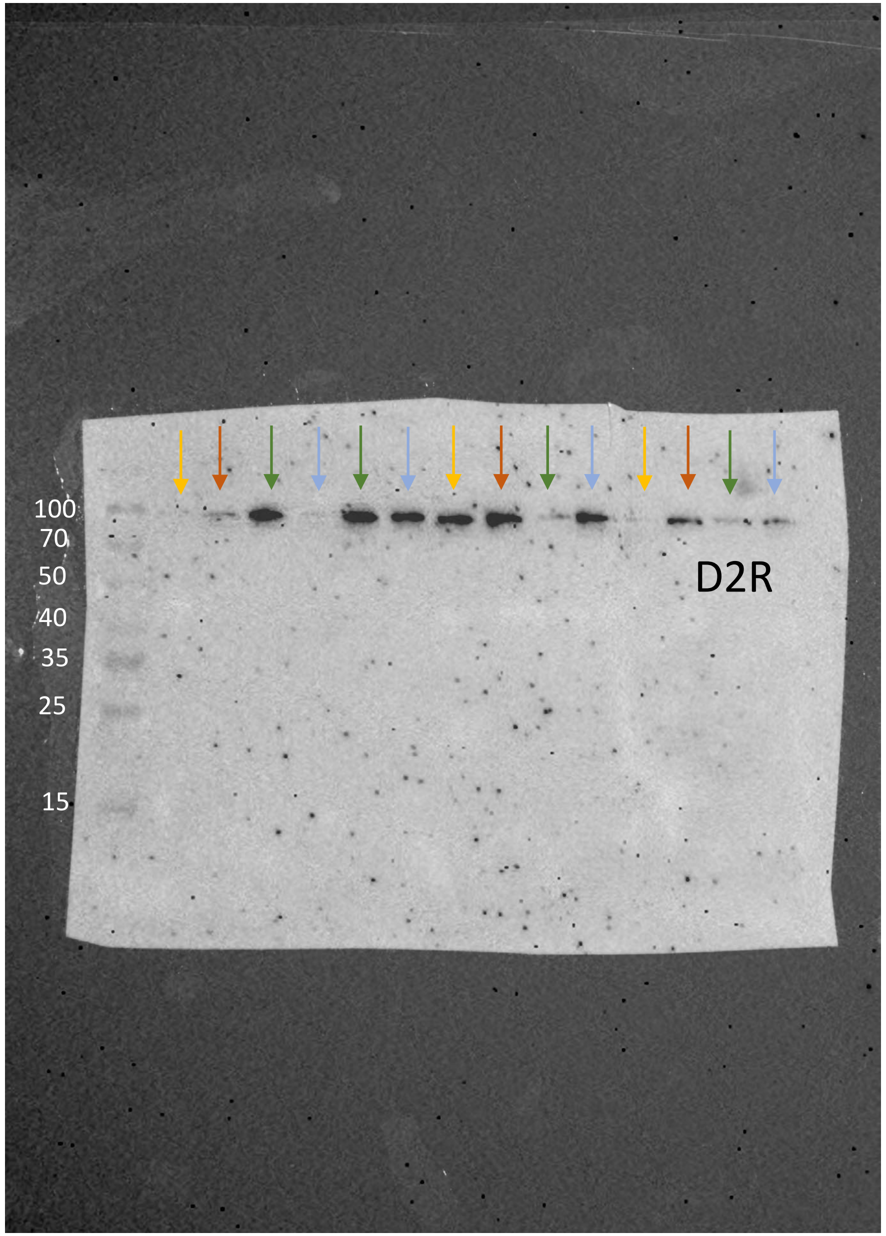

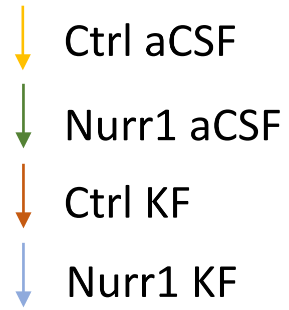

Nurr-1 Substantia Nigra Gels 5-6-7

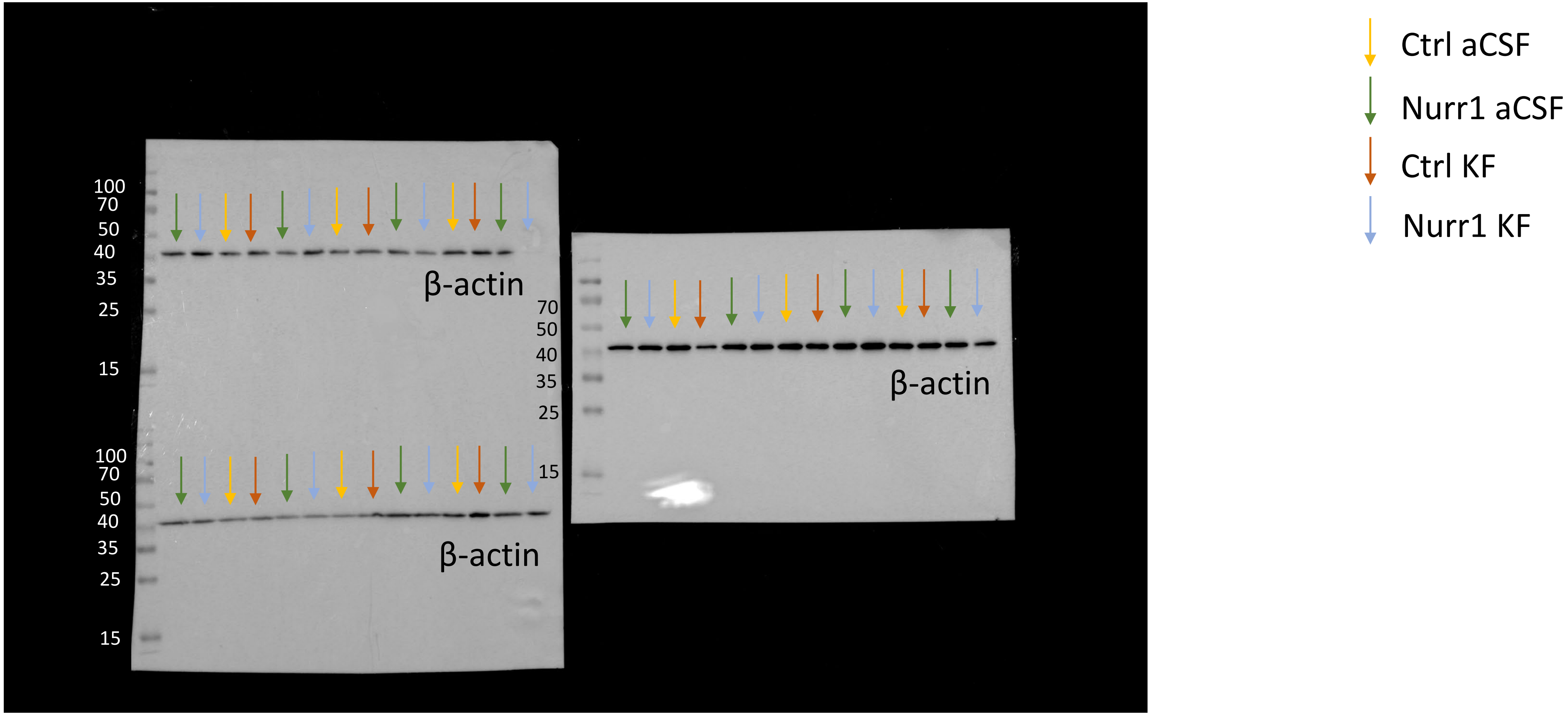

Nurr-1 Substantia Nigra Gels 5-6-7

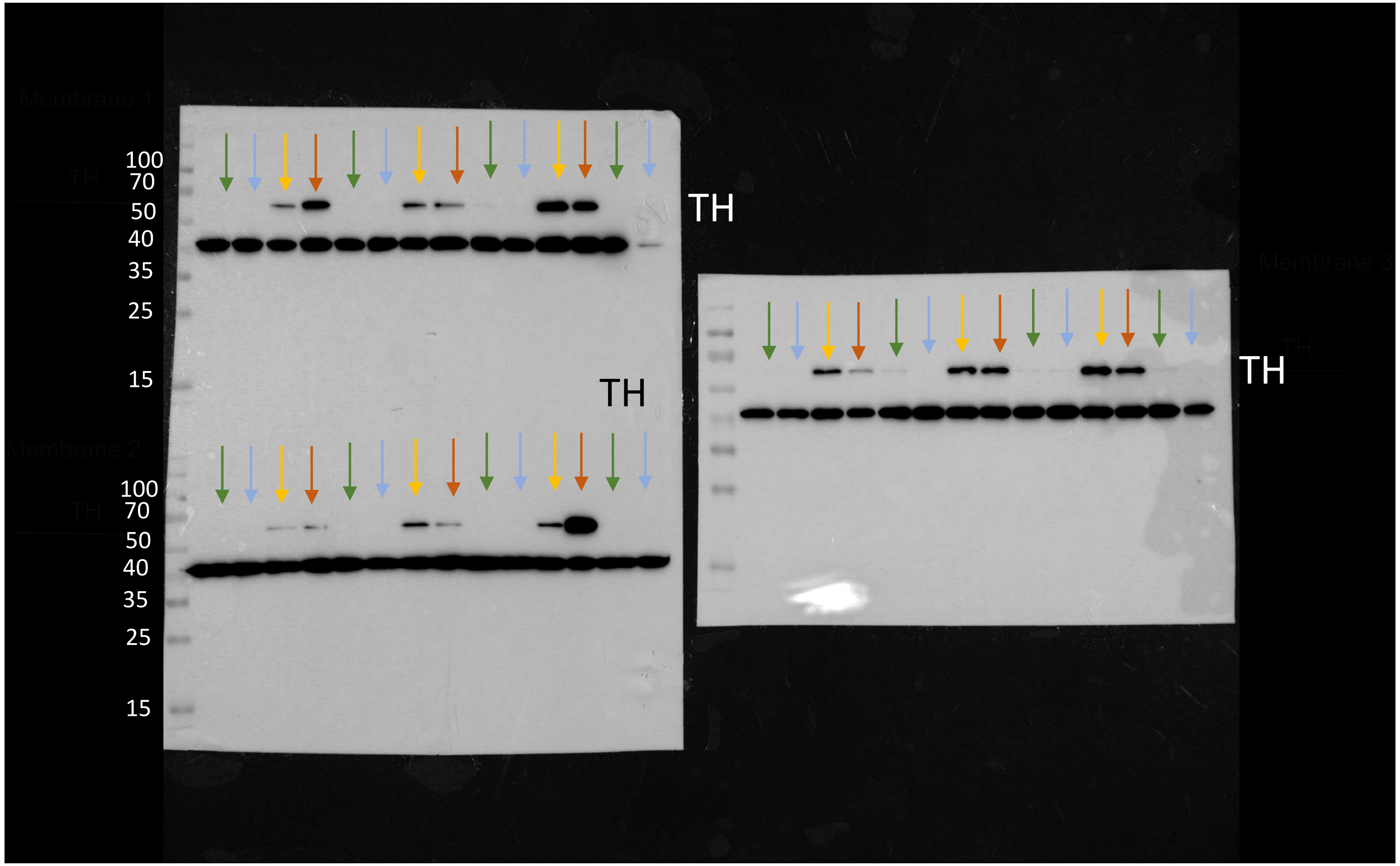

Ctrl aCSF

Nurr1 aCSF

Ctrl KF

Nurr1 KF

Nurr-1 Substantia Nigra Gels 5-6-7

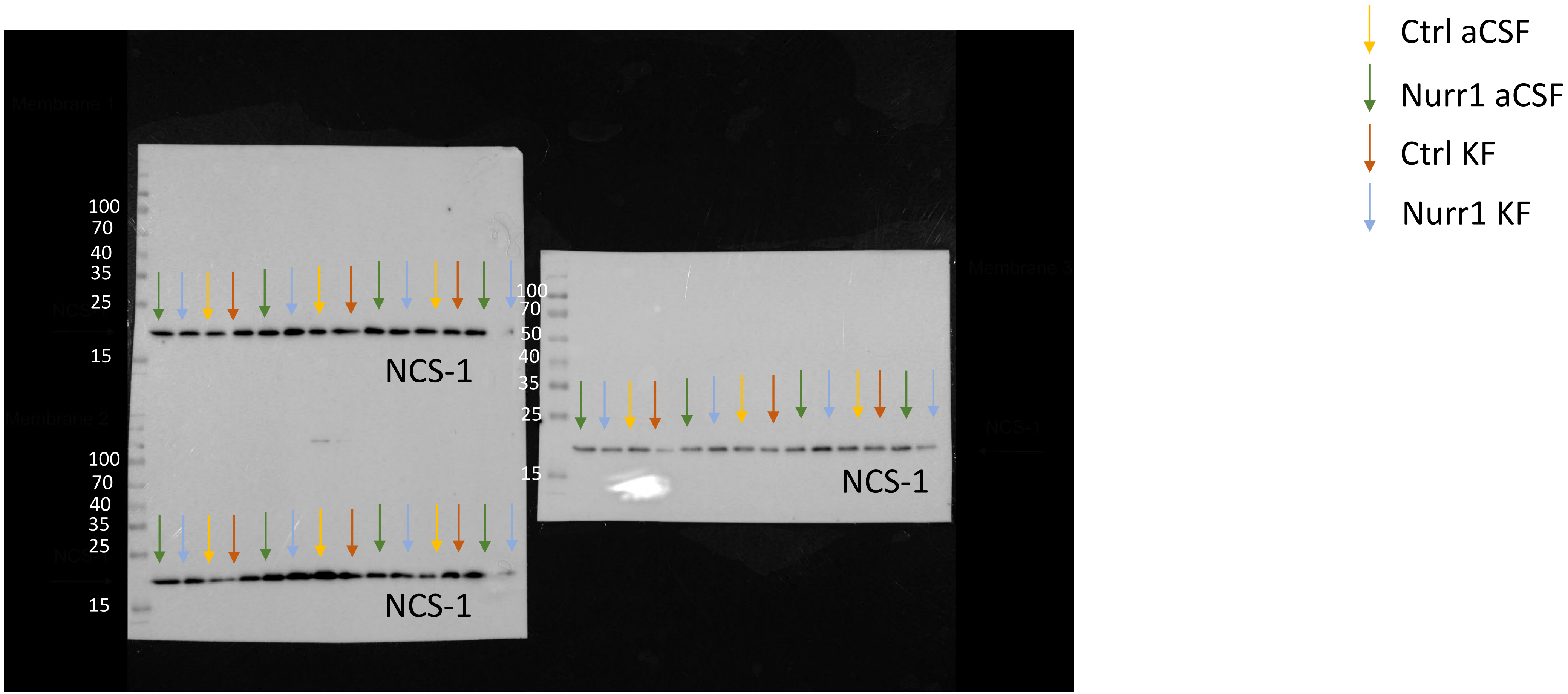

Nurr-1 Substantia Nigra Gels 5-6-7

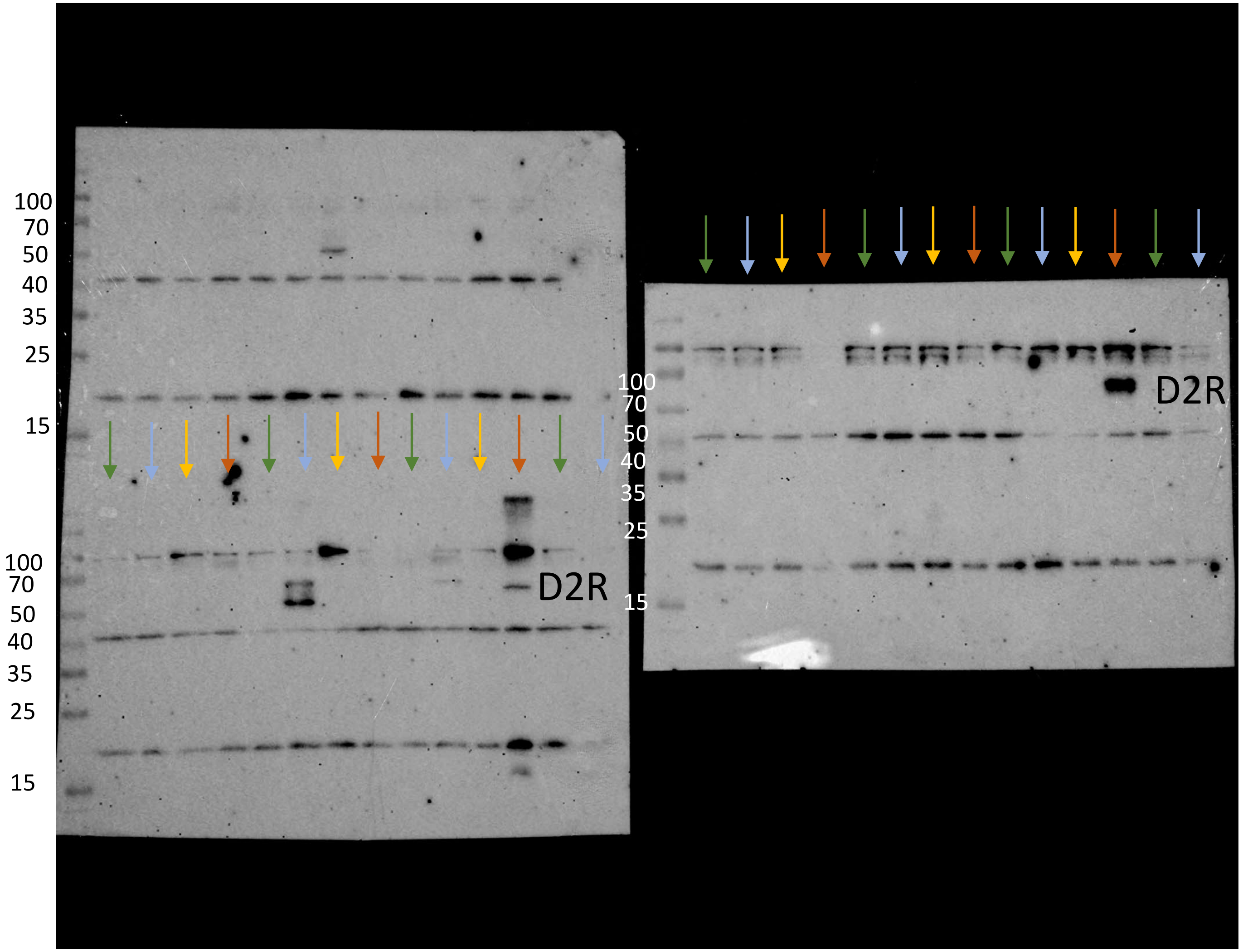

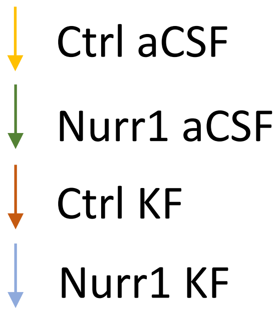

Nurr-1 Striatum Gel 3

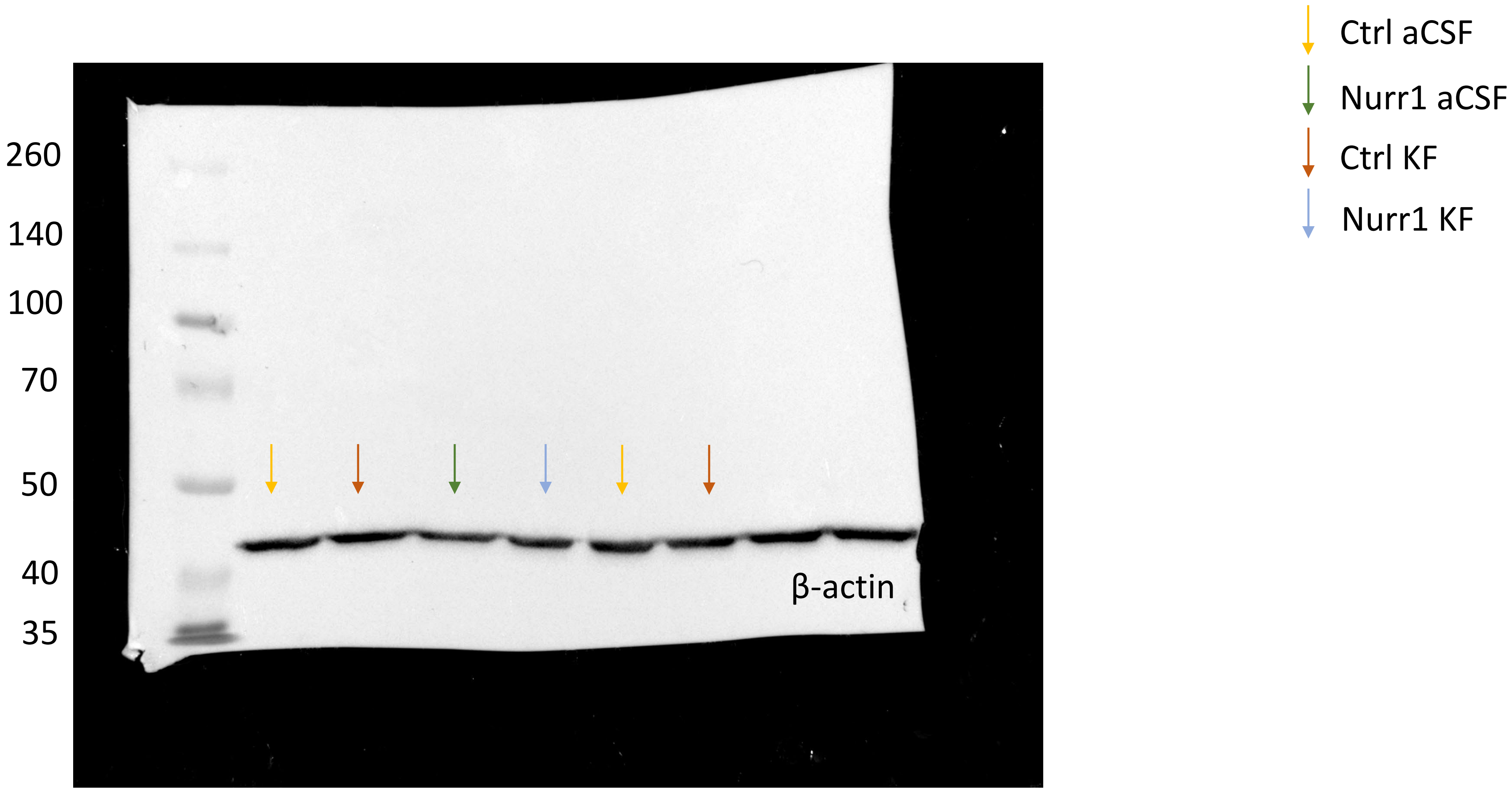

Nurr-1 Striatum Gel 3

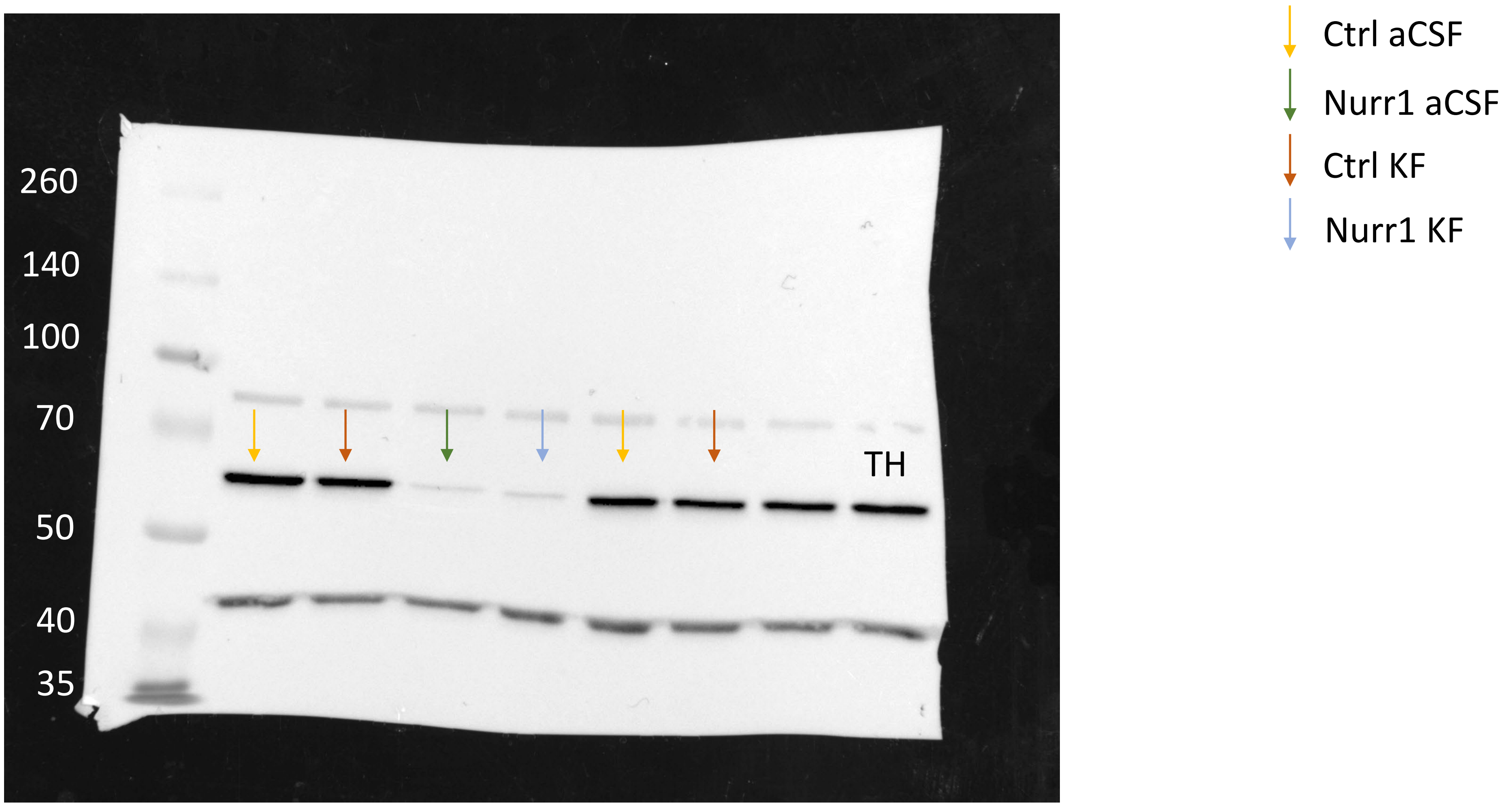

Nurr-1 Striatum Gel 3

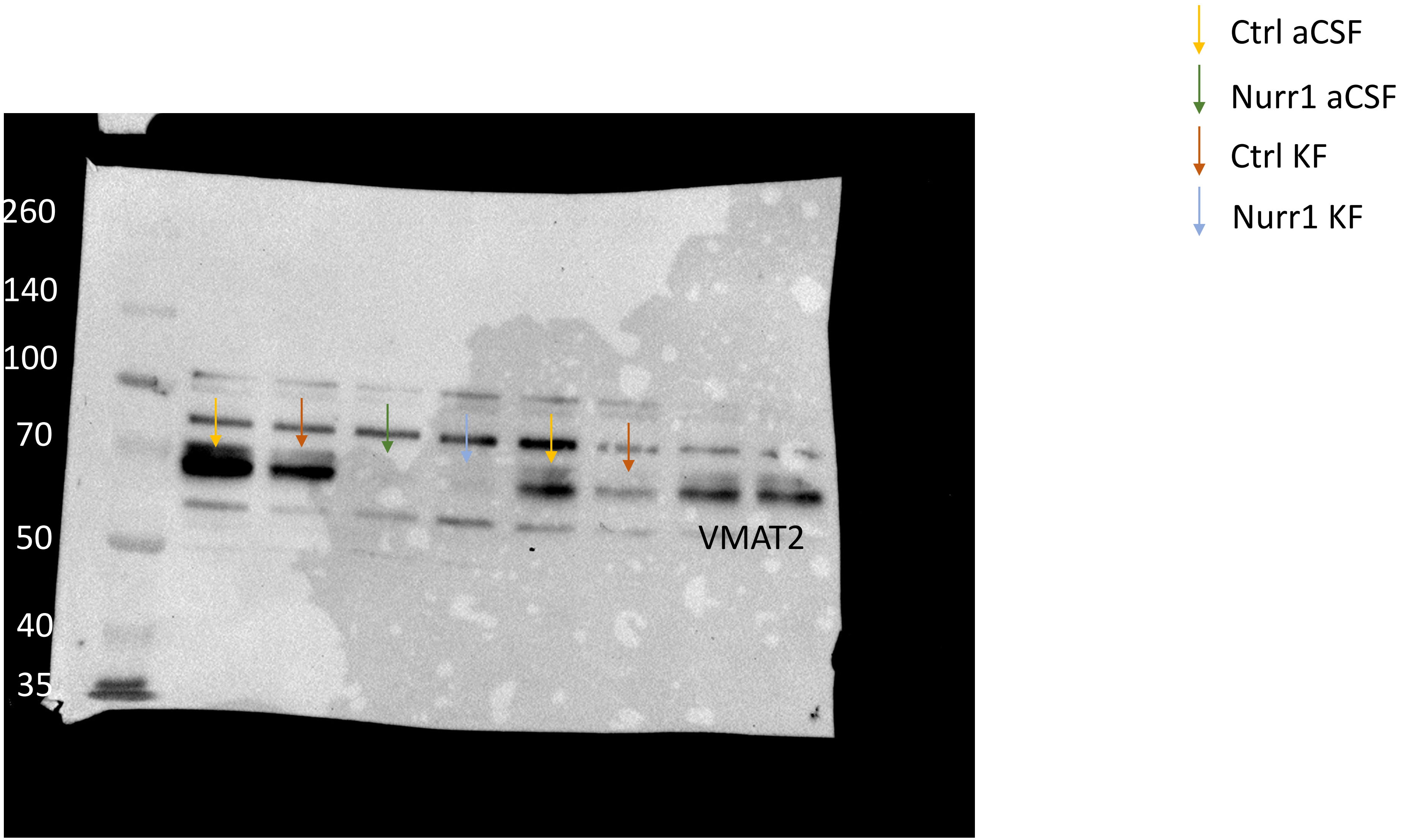

Nurr-1 Striatum Gel 4

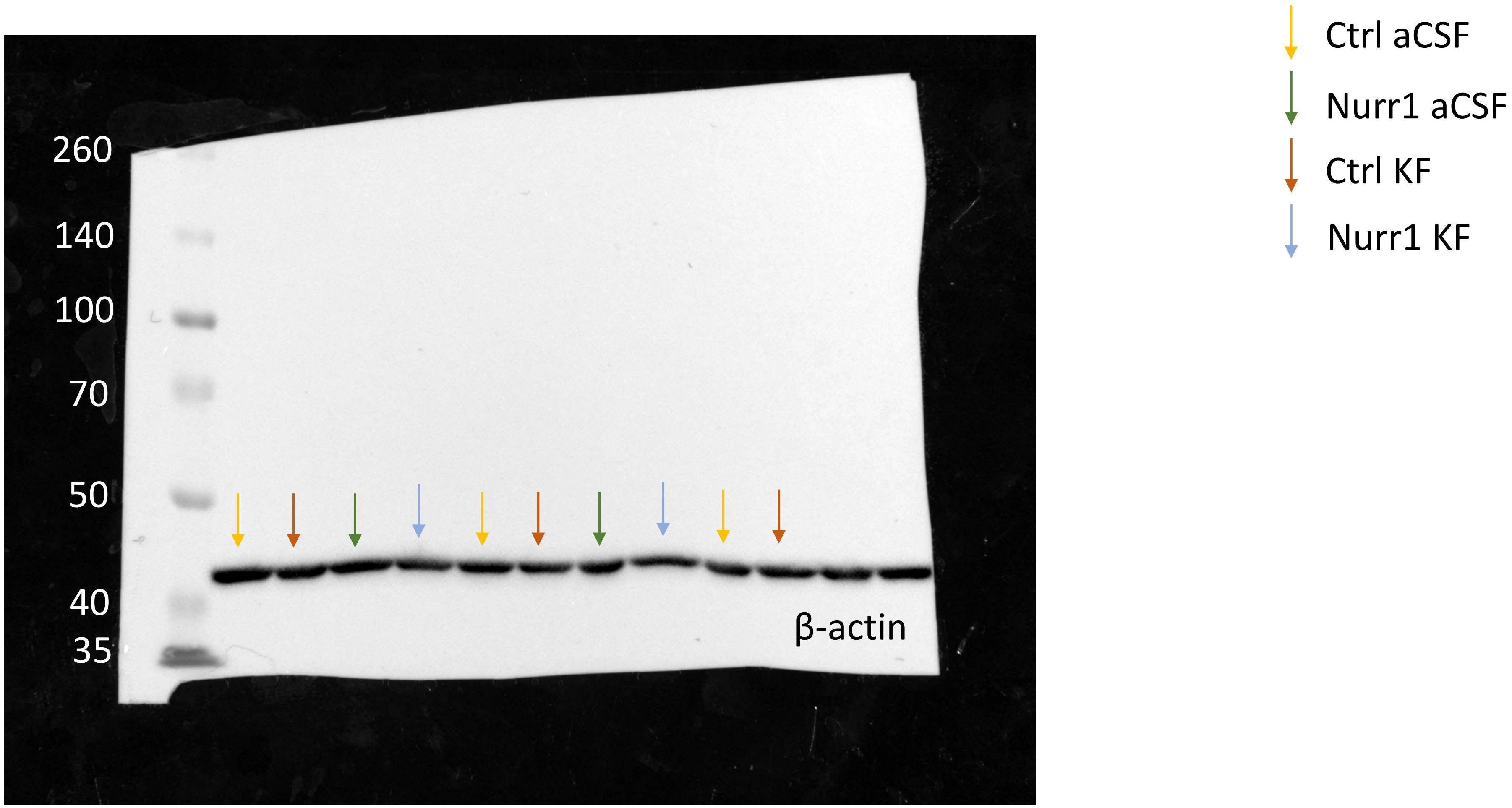

Nurr-1 Striatum Gel 4

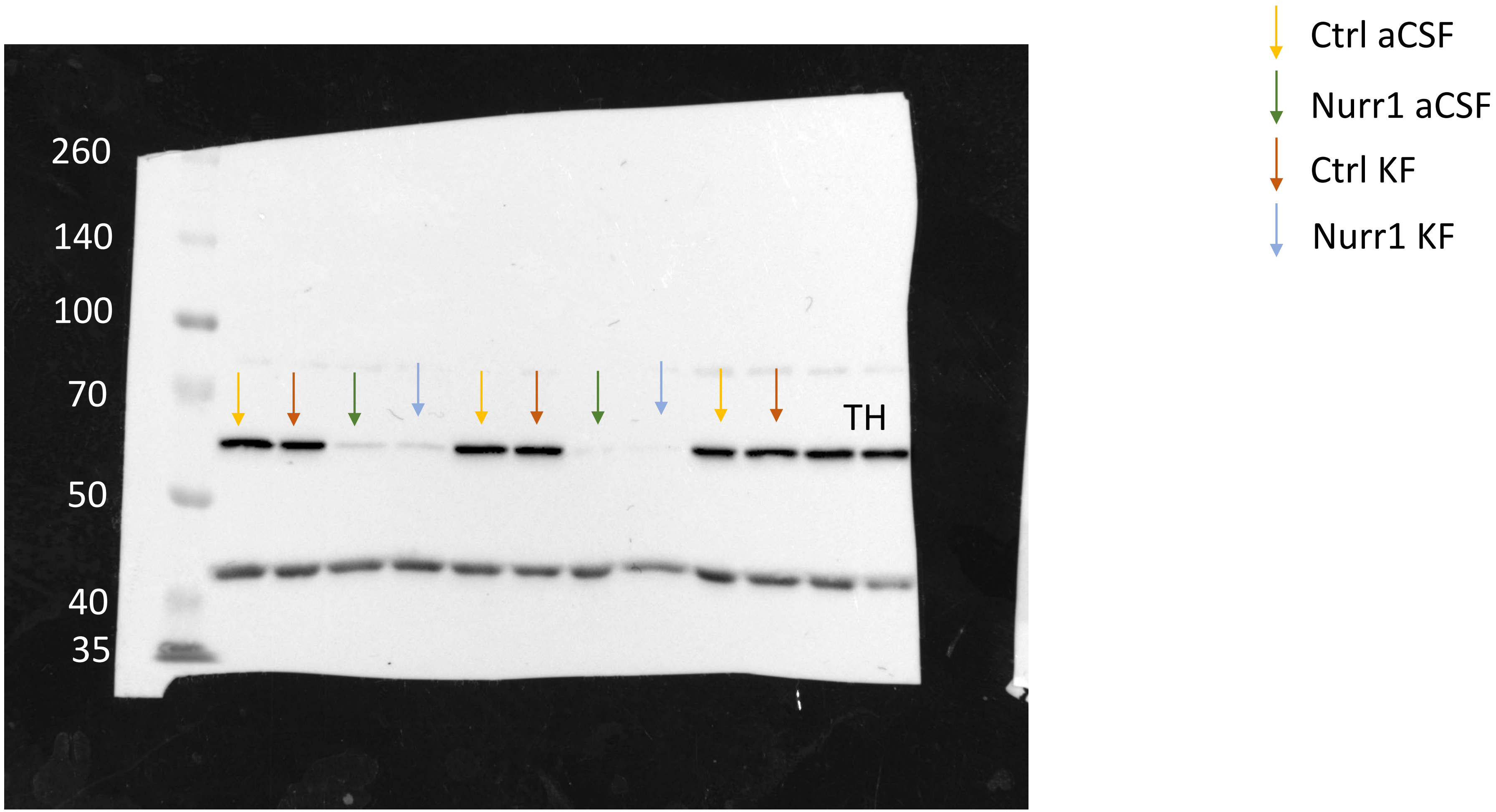

Nurr-1 Striatum Gel 4

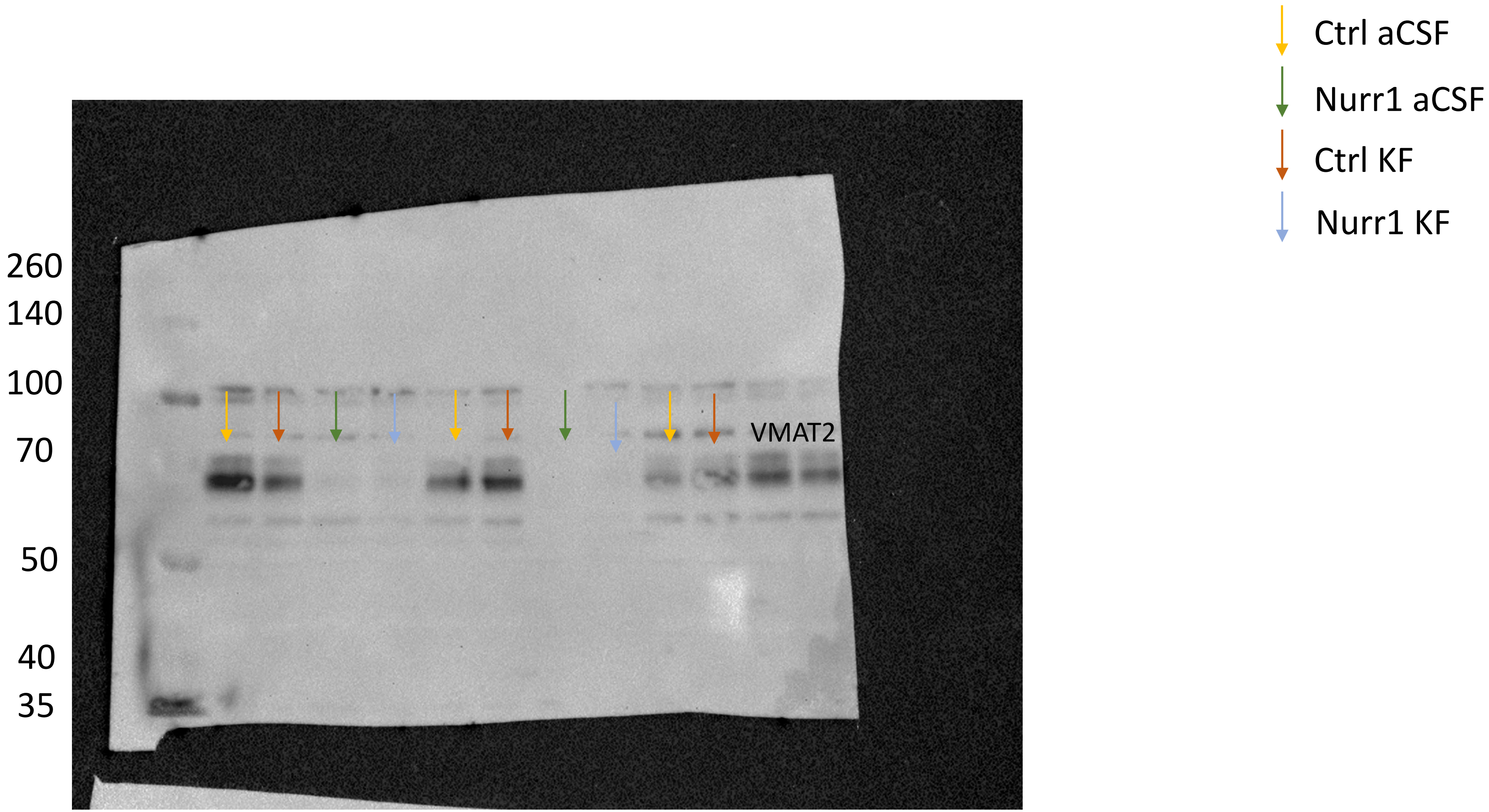

Nurr-1 Striatum Gel 6

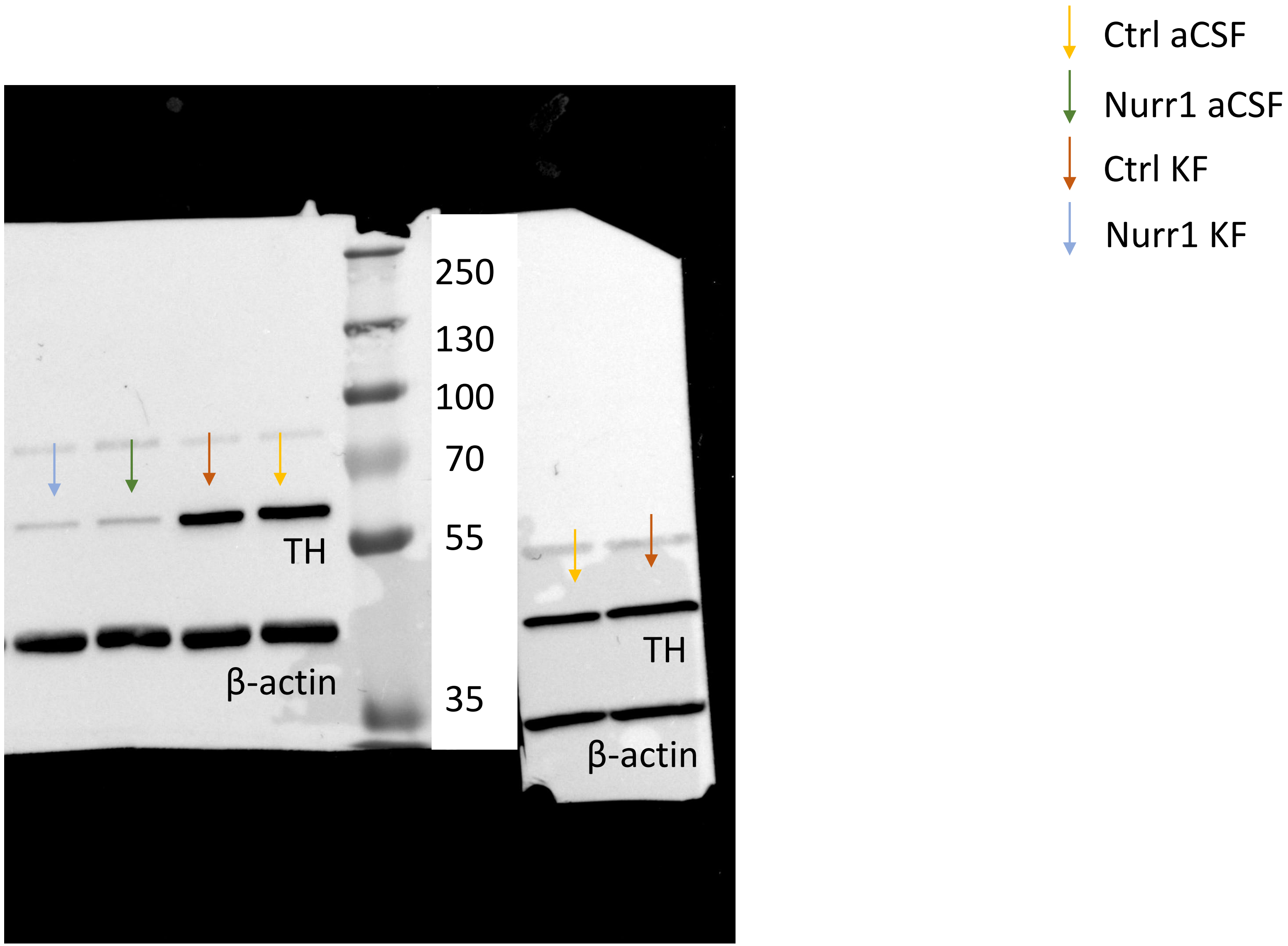

Nurr-1 Striatum Gel 6

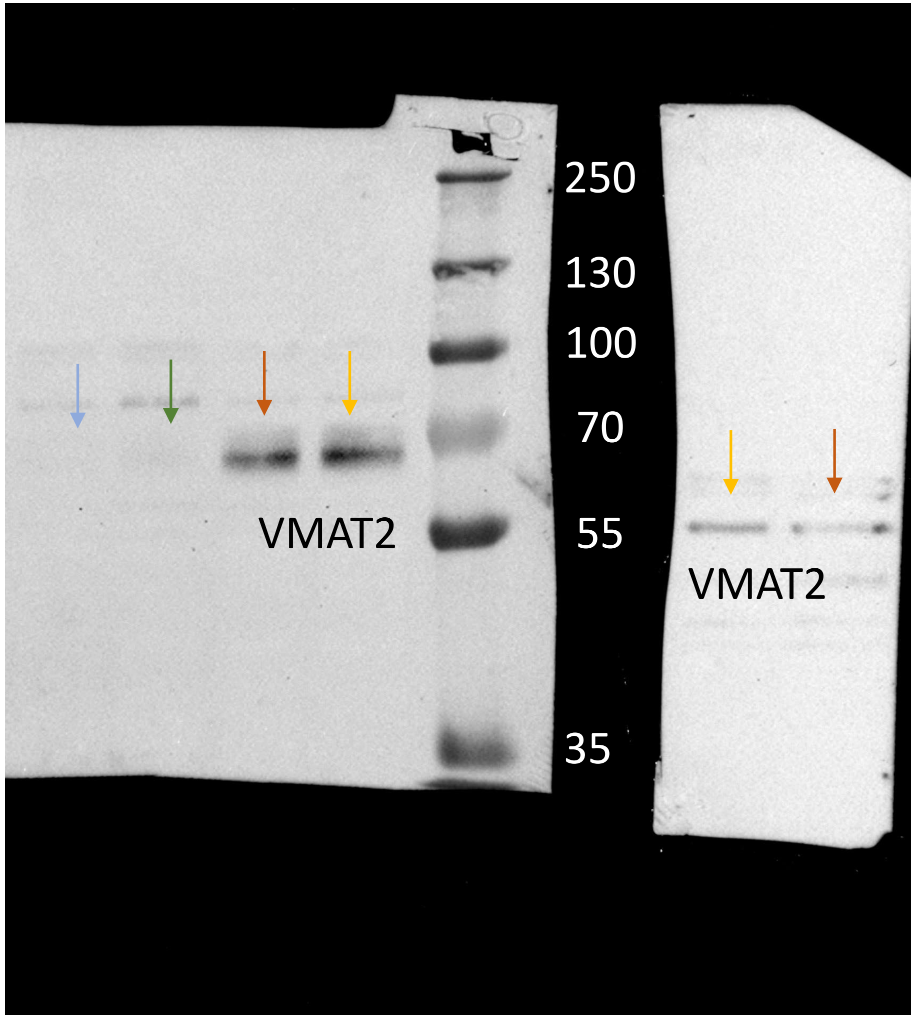

Nurr-1 Striatum Gel 7-10

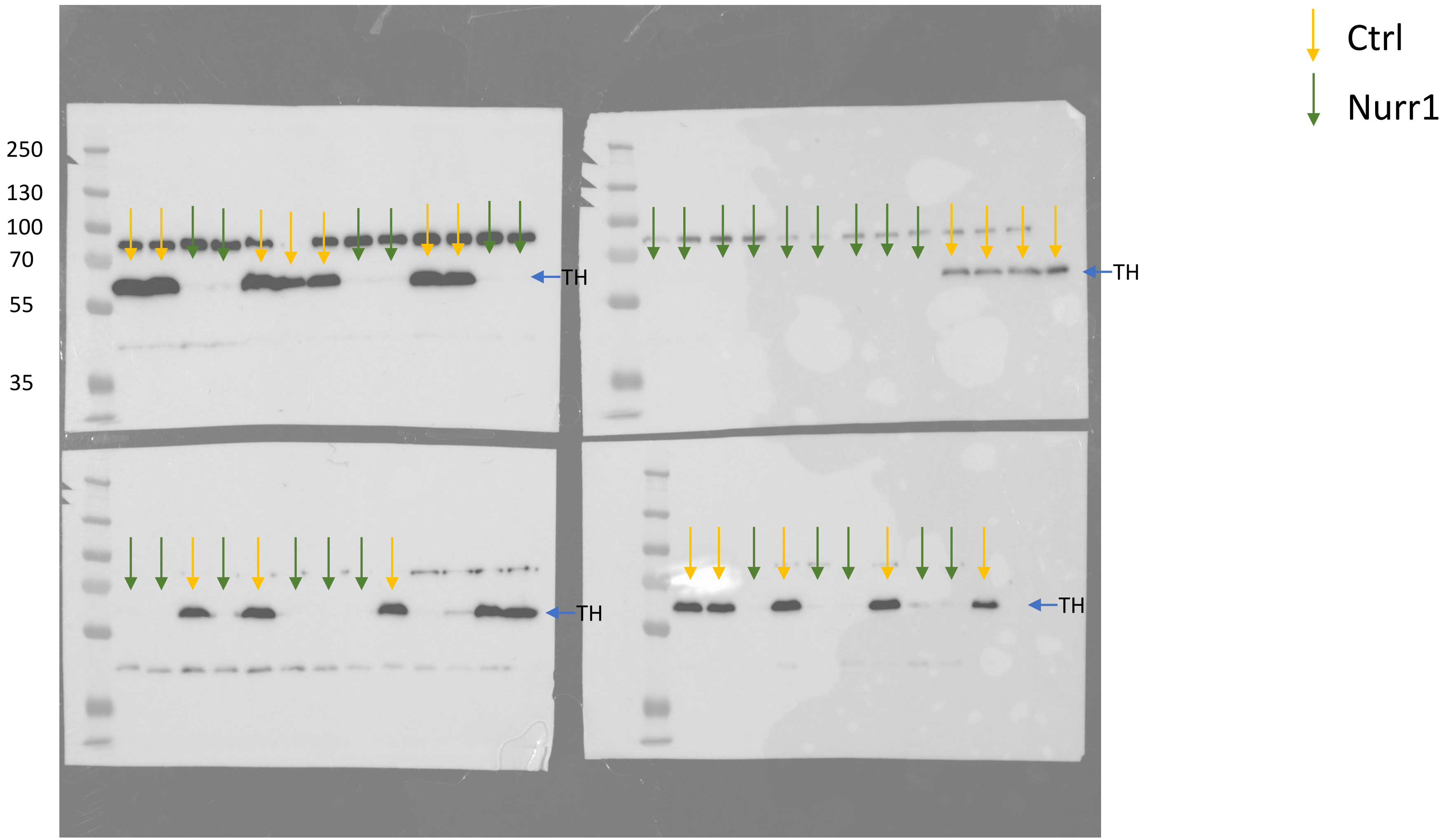

Nurr-1 Striatum Gel 7-10

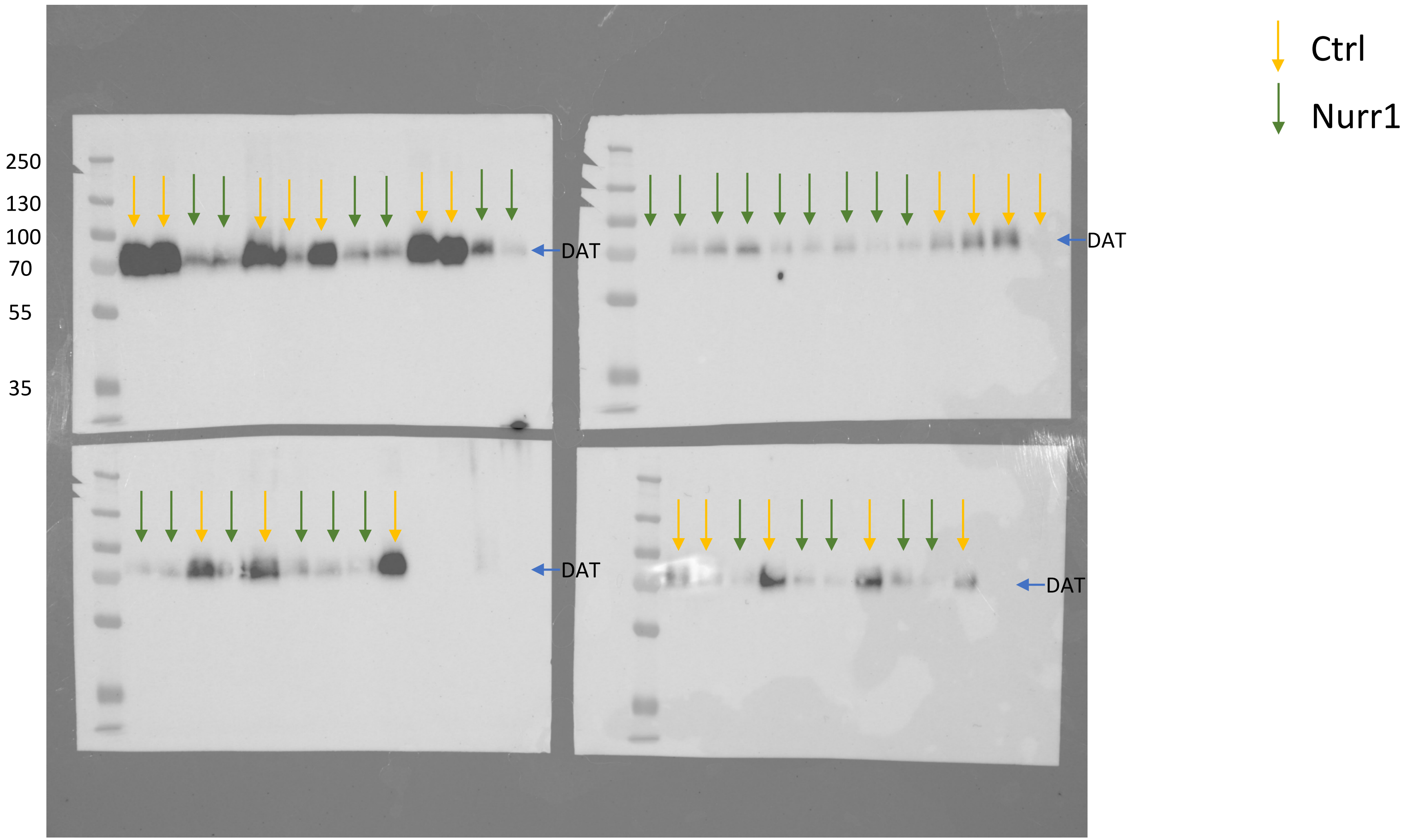

Nurr-1 Striatum Gel 7-10

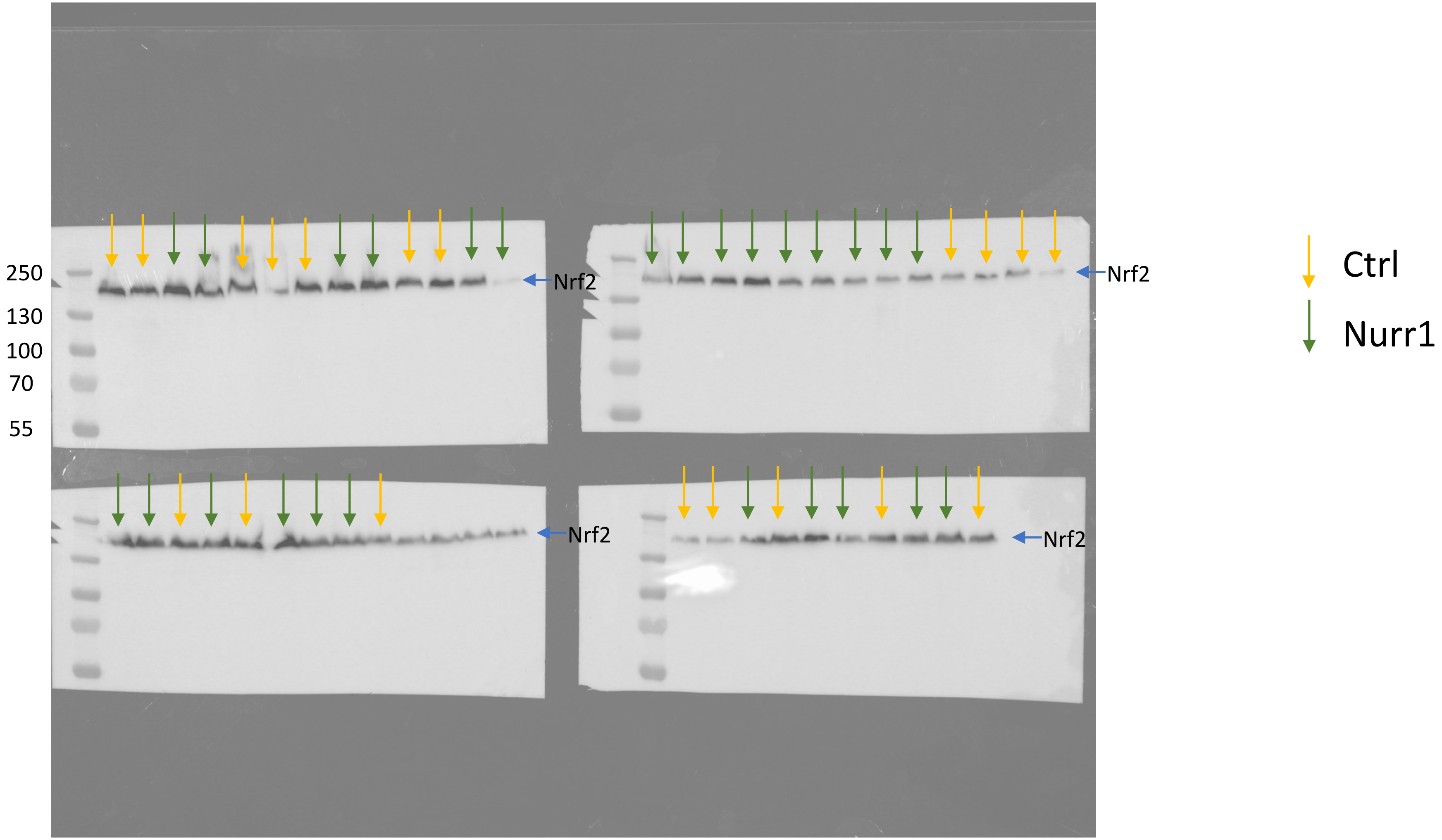

Nurr-1 Striatum Gel 7-10

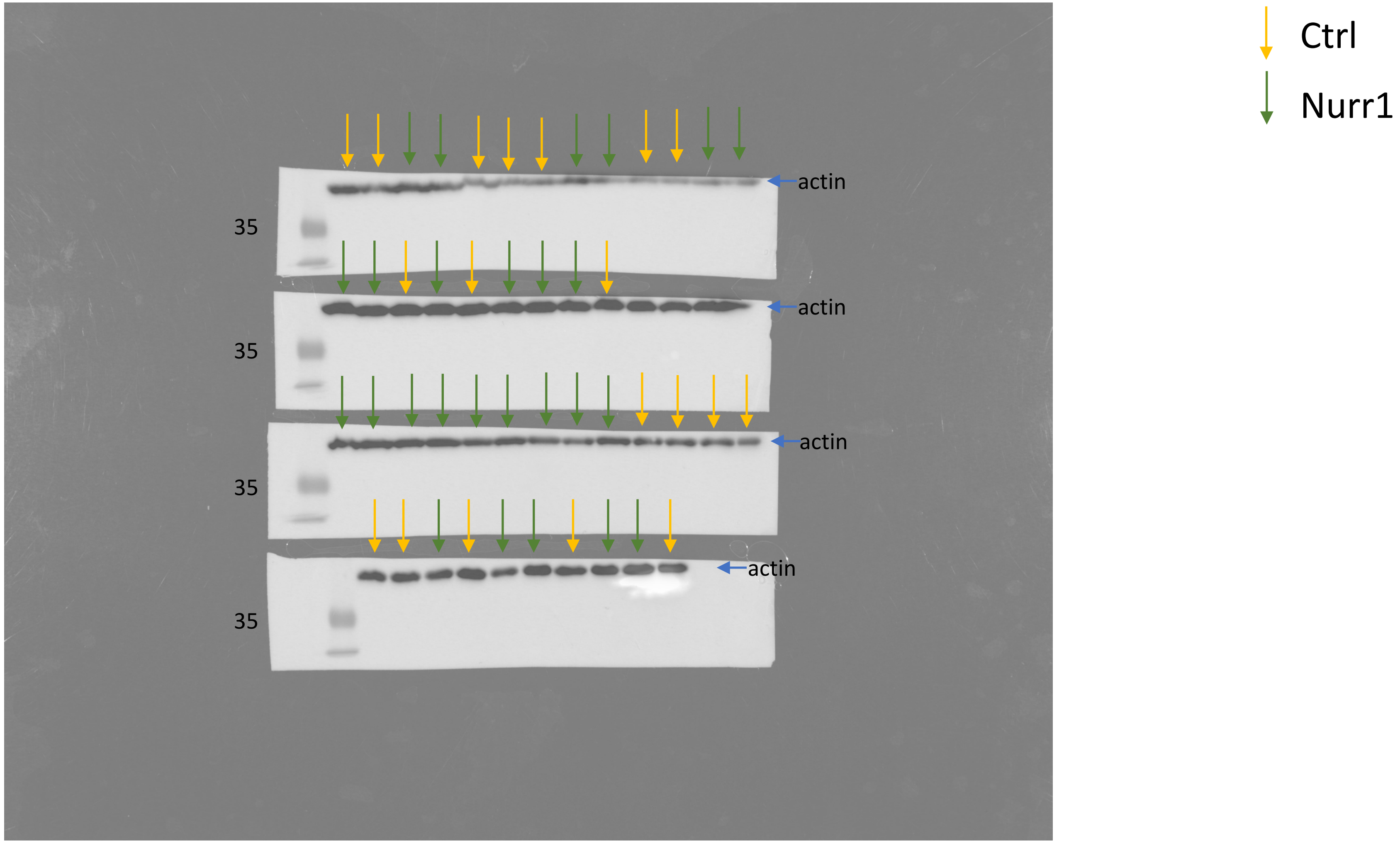

Nurr-1 SN Gel 8-11

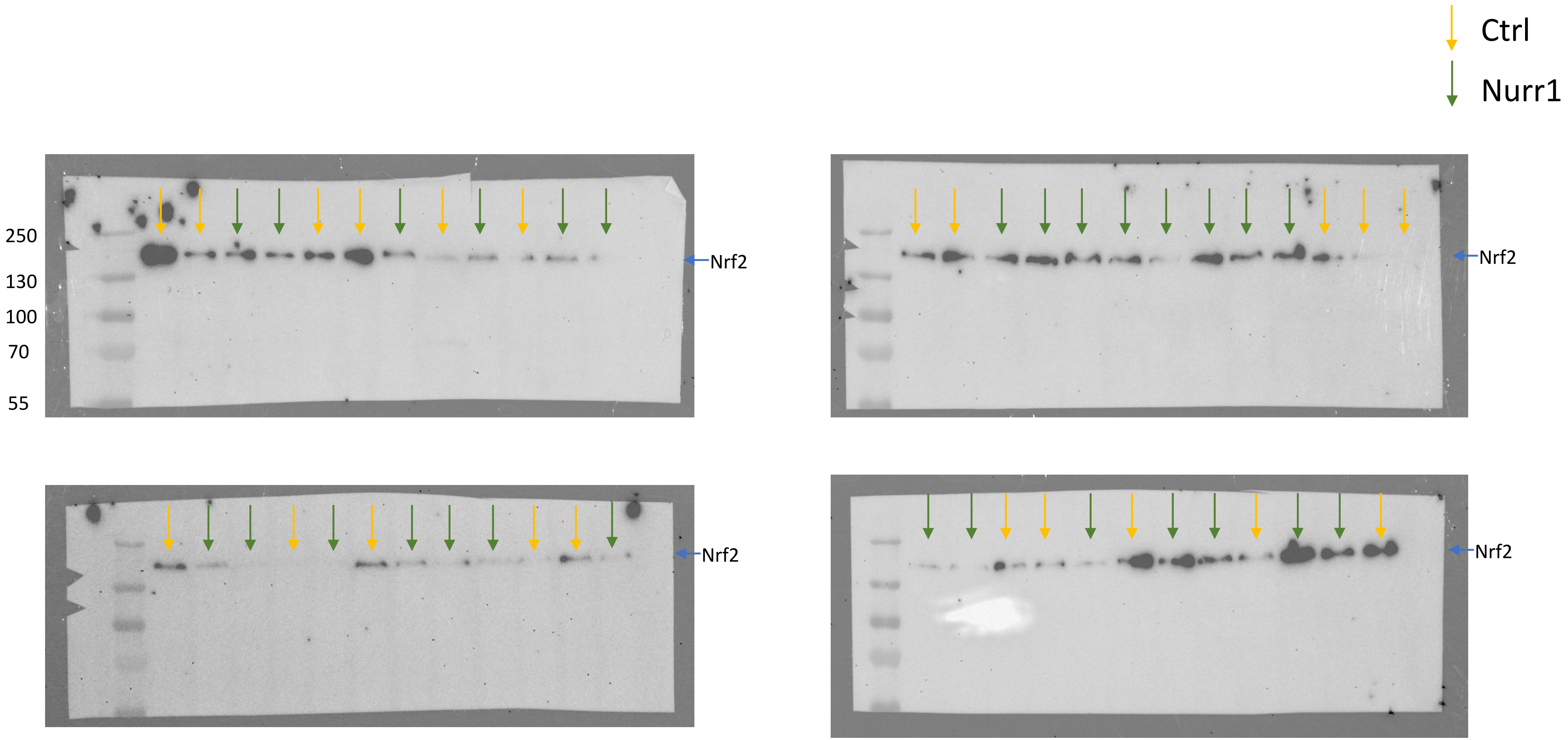

Nurr-1 SN Gel 8-11

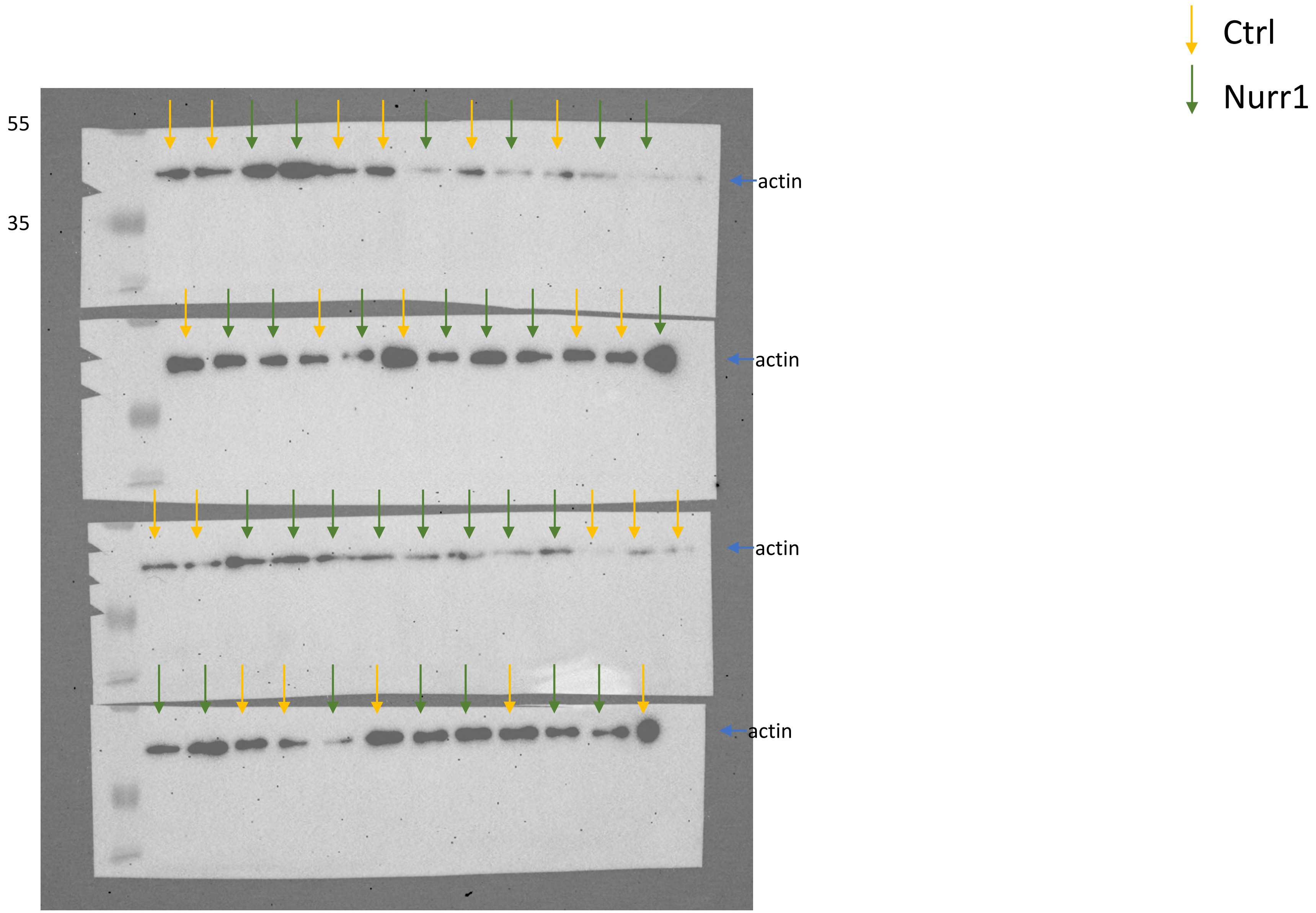

LRRK2 Substantia Nigra Gel 9-10

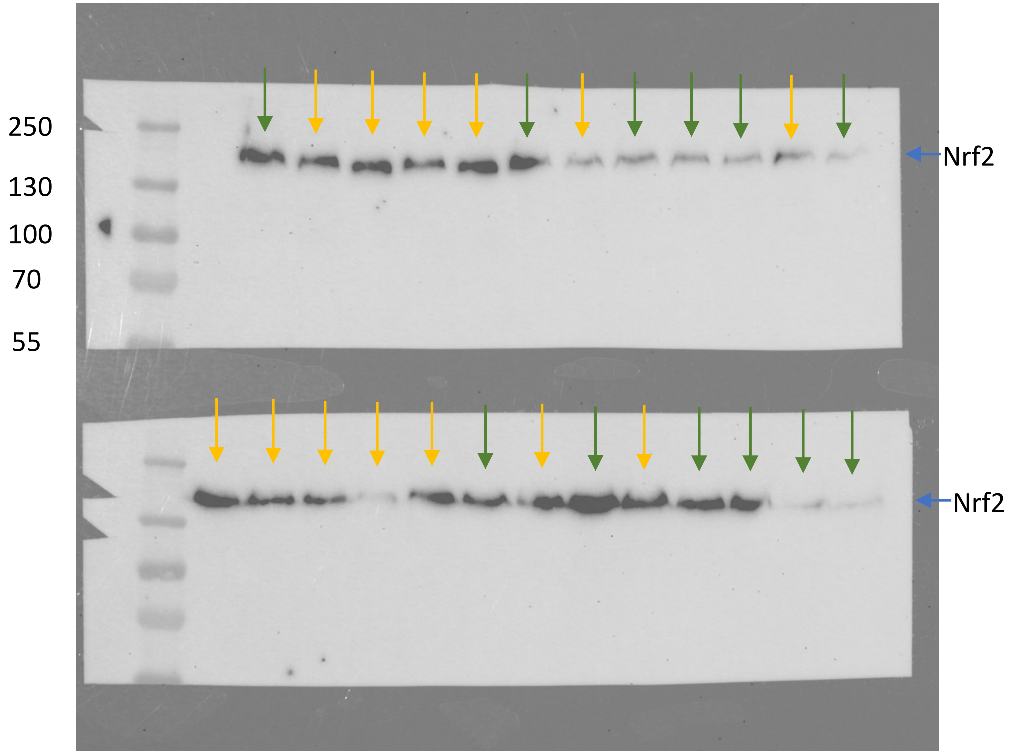

LRRK2 Striatum Gel 7-9

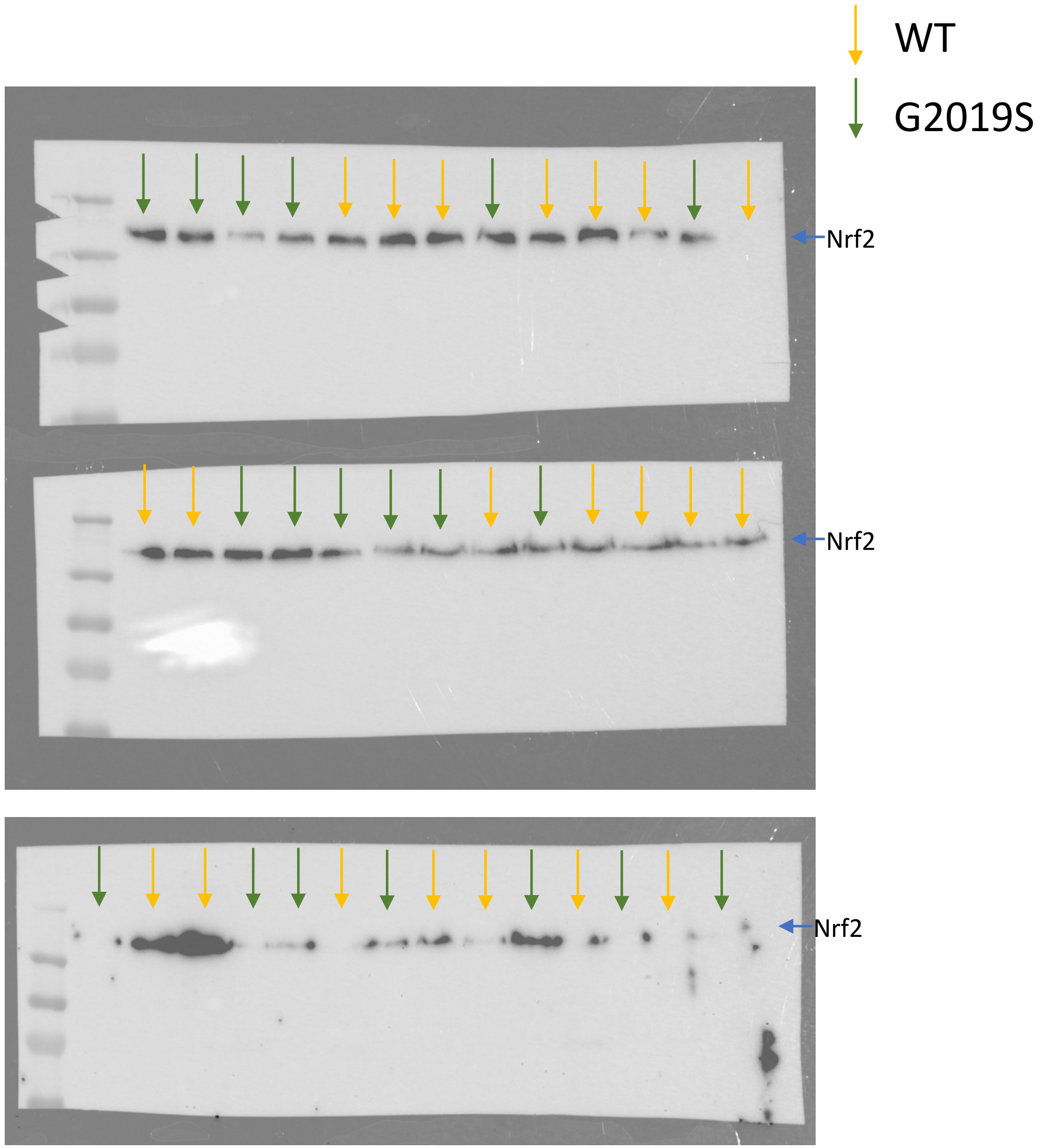

LRRK2 Substantia Nigra Gel 9-10

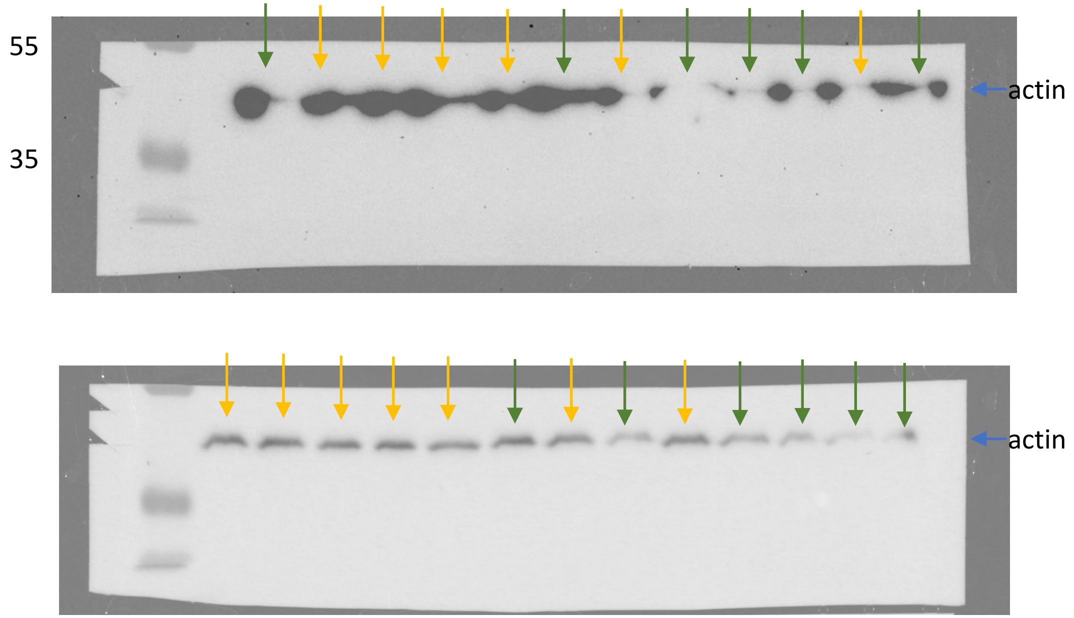

LRRK2 Striatum Gel 7-9

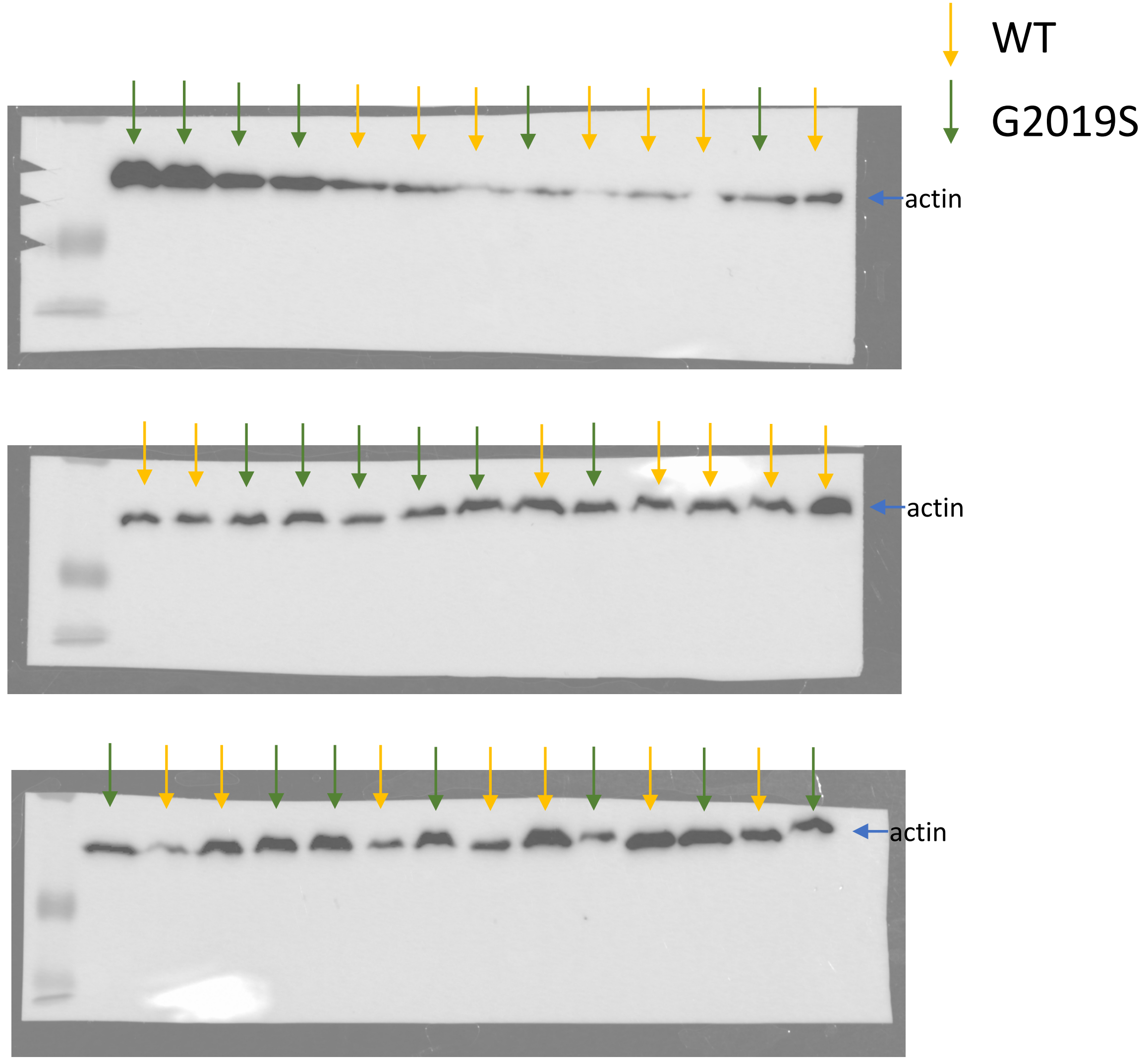

Supplement: Supplementary file 2 — Western blots [file 41531_2023_500_MOESM2_ESM.pdf]
