# Supplementary material for: Aberrant somatic calcium channel function in cNurr1 and LRRK2-G2019S mice
Source: NPJ Parkinsons Dis. 2023 Apr 7;9:56. doi: 10.1038/s41531-023-00500-5 (PMC10082048; doi:10.1038/s41531-023-00500-5)
Supplement: Supplementary file 3 — Reporting summary [file 41531_2023_500_MOESM3_ESM.pdf]

nature portfolio

Corresponding author(s): Karima Chergui

Last updated by author(s): Feb 22, 2023

Reporting Summary

Nature Portfolio wishes to improve the reproducibility of the work that we publish. This form provides structure for consistency and transparency in reporting. For further information on Nature Portfolio policies, see our Editorial Policies and the Editorial Policy Checklist.

Statistics

For all statistical analyses, confirm that the following items are present in the figure legend, table legend, main text, or Methods section.

n/a	Confirmed	
☐	☒	The exact sample size (*n*) for each experimental group/condition, given as a discrete number and unit of measurement
☐	☒	A statement on whether measurements were taken from distinct samples or whether the same sample was measured repeatedly
☐	☒	The statistical test(s) used AND whether they are one- or two-sided<br>*Only common tests should be described solely by name; describe more complex techniques in the Methods section.*
☐	☒	A description of all covariates tested
☐	☒	A description of any assumptions or corrections, such as tests of normality and adjustment for multiple comparisons
☐	☒	A full description of the statistical parameters including central tendency (e.g. means) or other basic estimates (e.g. regression coefficient) AND variation (e.g. standard deviation) or associated estimates of uncertainty (e.g. confidence intervals)
☐	☒	For null hypothesis testing, the test statistic (e.g. *F*, *t*, *r*) with confidence intervals, effect sizes, degrees of freedom and *P* value noted<br>*Give P values as exact values whenever suitable.*
☒	☐	For Bayesian analysis, information on the choice of priors and Markov chain Monte Carlo settings
☒	☐	For hierarchical and complex designs, identification of the appropriate level for tests and full reporting of outcomes
☒	☐	Estimates of effect sizes (e.g. Cohen's *d*, Pearson's *r*), indicating how they were calculated

Our web collection on statistics for biologists contains articles on many of the points above.

Software and code

Policy information about availability of computer code

Data collection	N.A.
Data analysis	N.A.

For manuscripts utilizing custom algorithms or software that are central to the research but not yet described in published literature, software must be made available to editors and reviewers. We strongly encourage code deposition in a community repository (e.g. GitHub). See the Nature Portfolio guidelines for submitting code & software for further information.

Data

Policy information about availability of data

All manuscripts must include a data availability statement. This statement should provide the following information, where applicable:

- Accession codes, unique identifiers, or web links for publicly available datasets
- A description of any restrictions on data availability
- For clinical datasets or third party data, please ensure that the statement adheres to our policy

The datasets generated during and/or analyzed during the current study are available from the corresponding author on reasonable request.

Human research participants

Policy information about studies involving human research participants and Sex and Gender in Research.

Reporting on sex and gender	N.A.
Population characteristics	N.A.
Recruitment	N.A.
Ethics oversight	N.A.

Note that full information on the approval of the study protocol must also be provided in the manuscript.

Field-specific reporting

Please select the one below that is the best fit for your research. If you are not sure, read the appropriate sections before making your selection.

☒ Life sciences ☐ Behavioural & social sciences ☐ Ecological, evolutionary & environmental sciences

For a reference copy of the document with all sections, see nature.com/documents/nr-reporting-summary-flat.pdf

Life sciences study design

All studies must disclose on these points even when the disclosure is negative.

Sample size	Sample sizes were based on our prior experience using the same paradigms.
Data exclusions	No data were excluded.
Replication	All experiments were performed several times and the data from these replications are pooled.
Randomization	Mice were allocated into specific groups based on their genotype, sex and age.
Blinding	The investigators were not blinded during the investigation or the data analysis.

Reporting for specific materials, systems and methods

We require information from authors about some types of materials, experimental systems and methods used in many studies. Here, indicate whether each material, system or method listed is relevant to your study. If you are not sure if a list item applies to your research, read the appropriate section before selecting a response.

Materials & experimental systems

n/a	Involved in the study
☐	☒ Antibodies
☒	☐ Eukaryotic cell lines
☒	☐ Palaeontology and archaeology
☐	☒ Animals and other organisms
☒	☐ Clinical data
☒	☐ Dual use research of concern

Methods

n/a	Involved in the study
☒	☐ ChIP-seq
☒	☐ Flow cytometry
☒	☐ MRI-based neuroimaging

Antibodies

Antibodies used

tyrosine hydroxylase (TH), Sigma-Aldrich, St Louis, USA, T2928, Mouse
β-actin, Sigma-Aldrich, St Louis, USA, A2228, Mouse
dopamine D2 receptor, Millipore, Temecula, USA, AB5084P, Rabbit
dopamine transporter (DAT), Millipore, Temecula, USA, MAB369, Rat
VMAT2, Abcam, Cambridge, UK, ab191121, Rabbit
NCS-1, Abcam, Cambridge, UK, ab129166, Rabbit
Nrf2, ThermoFisher, Rockford, USA, PA5-27882, Rabbit
tyrosine hydroxylase (TH), Millipore, Temecula, USA, AB152, Rabbit

Validation	tyrosine hydroxylase (TH), Sigma-Aldrich, St Louis, USA, T2928, Mouse, RRID (AB_2313844)<br>β-actin, Sigma-Aldrich, St Louis, USA, A2228, Mouse,RRID (AB_476697)<br>dopamine D2 receptor, Millipore, Temecula, USA, AB5084P, Rabbit, RRID (AB_2094980)<br>dopamine transporter (DAT), Millipore, Temecula, USA, MAB369, Rat, RRID (AB_2190413)<br>VMAT2, Abcam, Cambridge, UK, ab191121, Rabbit, RRID (Not found), Reference provided by Abcam (Nyarko JNK et al. Glycosylation States of Pre- and Post-synaptic Markers of 5-HT Neurons Differ With Sex and 5-HTTLPR Genotype in Cortical Autopsy Samples. Front Neurosci 12:545 (2018).)<br>NCS-1, Abcam, Cambridge, UK, ab129166, Rabbit, RRID (AB_11150438)<br>Nrf2, ThermoFisher, Rockford, USA, PA5-27882, Rabbit, RRID (AB_2545358)<br>tyrosine hydroxylase (TH), Millipore, Temecula, USA, AB152, Rabbit, RRID (AB_390204)

Animals and other research organisms

Policy information about studies involving animals; ARRIVE guidelines recommended for reporting animal research, and Sex and Gender in Research

Laboratory animals	BAC-DAT-CreERT2 mice, 6-8 months old and BAC hLRRK2-G2019S mice, 10-12 months old.
Wild animals	The study did not involve wild animals.
Reporting on sex	Male and female mice were used. No sex-related differences were observed and the data were pooled.
Field-collected samples	The study did not involve data from field-collected samples.
Ethics oversight	Animal experiments were approved by our local ethical committee (Stockholms norra djurförsöksetiska nämnd, 20464-2020).

Note that full information on the approval of the study protocol must also be provided in the manuscript.
